# Identification of autophagy‐related genes signature predicts chemotherapeutic and immunotherapeutic efficiency in bladder cancer (BLCA)

**DOI:** 10.1111/jcmm.16552

**Published:** 2021-05-07

**Authors:** Rui Cao, Bo Ma, Gang Wang, Yaoyi Xiong, Ye Tian, Lushun Yuan

**Affiliations:** ^1^ Department of Urology Beijing Friendship Hospital Capital Medical University Beijing China; ^2^ Department of Stomatology Beijing Shijitan Hospital Capital Medical University Beijing China; ^3^ Department of Biological Repositories Zhongnan Hospital of Wuhan University Wuhan China; ^4^ Department of Urology Zhongnan Hospital of Wuhan University Wuhan China; ^5^ Department of Internal Medicine Division of Nephrology Leiden University Medical Center Leiden The Netherlands

**Keywords:** autophagy, bladder cancer, chemotherapy, immune infiltration, immunotherapy

## Abstract

Autophagy maintains cellular homeostasis by degrading and recycling cytoplasmic components under stress conditions, which is identified to be involved in tumorigenesis and now has been recognized as novel target in cancer treatment. In present study, we gathered total autophagy‐related genes and established an autophagy‐related genes signature (ATGRS) through LASSO cox regression analysis in BLCA. Kaplan‐Meier survival and multivariate cox regression analyses both showed the ATGRS was a robust independent prognostic factor with high accuracy. Subsequently, integrated analyses indicated that ATGRS had a strong correlation with molecular subtypes, clinicopathological characteristics and somatic mutation alteration. Moreover, ATGRS was found to be positively correlated with the infiltration of immune cells in tumour microenvironment (TME) and immune checkpoint expression, indicating the potent role of autophagy by regulating the TME. In addition, ATGRS was proved to be efficient in predicting the clinical benefit of immune checkpoint inhibitors (ICIs) based immunotherapy and chemotherapy in BLCA. Furthermore, we observed abnormal expression levels of autophagy‐related genes and found the different behaviour of ATGRS in pancancer by LASSO cox regression analysis. Therefore, construction of ATGRS in BLCA could help us to interpret the underlying mechanism of autophagy and sheds a light on the clinical application for a combination of autophagy modification with targeted immunotherapy and chemotherapy in BLCA.

## 1 BACKGROUND

Bladder cancer (BLCA) is a life‐threatening malignancy with high mortality and morbidity, which is considered as one of the most expensive tumours in terms of treatment and medical care.^1^ In 2018, BLCA was recognized as the 10th most common cancer with estimated 549,393 new cases and 199 922 deaths around the globe.^2^ BLCA is comprised of several histological subtypes, but nearly >90% cases are transitional cell carcinoma.^3^ Among the new cases, 70% are non‐muscle invasive (NMIBC) and 30% are muscle‐invasive bladder cancer (MIBC). Even though receiving the standard treatment procedure of transurethral resection of bladder tumour (TURBT) and subsequent intra‐vesicular administration, not all the patients with NMIBC benefit from this, over 50% of the patients will finally relapse, and 20% continue to progress within 5 years.^4^ MIBC is such a complex disease that a multidisciplinary approach and systemic therapy, involving surgery combined with radiotherapy and chemotherapy were used to prevent its progression. Regretfully, we found that 50% overall survival (OS) at 5 years for MIBC hasn't relatively changed for 20 years. While management of other cancers has developed rapidly, the management of BLCA remains relatively stagnant.^5^, ^6^ Recently, immune checkpoint inhibitors (ICIs) targeting cytotoxic T‐lymphocyte‐associated protein 4 (CTLA‐4) and the programmed cell death‐1 (PD‐1)/programmed cell death‐ligand‐1 (PD‐L1) pathway were developed and showed a robust and durable responses in patients with various cancers,^7^, ^8^ including BLCA.^9^ However, not all patients with advanced cancer respond to ICIs. The response rate is only modestly above the historical 10% higher than that in traditional chemotherapies. The current staging and grade system, which only focuses on the anatomy of malignancy, could not guide the efficiency of precision treatment, especially for target immunotherapy treatment.^10^ Therefore, in order to improve clinical outcomes, understanding the underlying mechanism and biology of BLCA and development of novel biomarkers to predict the responses to current therapies is urgently required.

Autophagy is an evolutionary conserved catabolic process and plays a vital role in maintaining cellular homeostasis by degrading and recycling damaged cytoplasmic components, including macromolecules and organelles under various cellular stress conditions, for example nutrient deprivation, hypoxia, organelle damage, radiotherapy or chemotherapy to satisfy cellular needs and promote cell survival.^11^, ^12^, ^13^, ^14^, ^15^ Therefore, autophagy was reported to be involved in numerous biological and pathological processes, including neurodegenerative diseases, pathogenic inflammation, aging and cancer.^16^ However, autophagy is a double‐edged sword in oncogenesis which either suppresses or promotes tumour development in a context‐dependent manner which is associated with tumour type, clinical stage, genetic background and even therapeutic regimen.^17^ During the early stages of cancer, autophagy usually act as a tumour suppressor by maintaining chromosomal stability and preventing proliferation and inflammation ^18^ Nevertheless, once the cancer has been formed, autophagy allows tumour cells to recycle the remodelling components and refuel the energy supply to alleviate cellular damage and survive under cellular stresses, and thus promoting tumour progression.^19^, ^20^, ^21^ In addition, the role of autophagy in cancer therapy appears to be more paradoxical. Some studies demonstrated that anticancer therapy such as chemotherapy could induce cellular protective autophagy, which is one of the main causes of chemotherapy resistance.^22^ Therefore, autophagy inhibition gives us a new strategy to increase the cytotoxic effect of treatments, including chemotherapy and radiotherapy. On the contrary, excessive promotion of autophagy may induce type II programmed cell death, which is referred as autophagic cell death, similar to apoptosis and is defined as cell death in the presence of lysosomes.^23^

The role of autophagy for onset and progression of BLCA has been reported by Ojha et al. This group demonstrated that high‐grade BLCA tumours exhibited more autophagic vesicles when compared with low‐grade tumours and benign tissues. They also found that up‐regulation of Beclin‐1 and ATG7, as well as conversion of LC3‐I to LC3‐II in tumour specimens, were significantly associated with autophagosome biogenesis and autophagy induction. Furthermore, the autophagy inhibitors, such as wortmannin, 3‐methyladenine (3MA) and chloroquine, could remarkably increase cell death in high‐grade tumour samples compared with low‐grade tumour samples.^24^ Zhu et al revealed that ATG7 was notably overexpressed in invasive BLCA tissues and knockdown of ATG7 could clearly inhibit BLCA cells invasion, suggesting that ATG7 was involved in the regulation of BLCA development.^25^ Some studies also suggested that targeting autophagy could improve sensitivity of chemotherapy agents in treatment of BLCA.^26^, ^27^ Thus, exploring the appropriate molecular biomarkers based on autophagy could not only predict disease prognosis, but also provide clinicians with a rationale for autophagy targeting therapies in BLCA.

In the present study, we have established a scoring system, called autophagy‐related genes signature (ATGRS) through LASSO cox regression analysis. ATGRS was effective in predicting the overall survival (OS) and disease‐free survival (DFS) of BLCA patients in several independent cohorts. Integrated analyses indicated that ATGRS was strikingly correlated with clinicopathological characteristics, molecular subtypes, somatic mutational landscape and infiltration of immune cells. Moreover, we amazed to find that ATGRS could predict the clinical benefit of ICIs immunotherapy and chemotherapy efficiently. In summary, we developed a novel ATGRS, which exhibited a potential prognostic value for BLCA patients and might facilitate personalized counselling for immunotherapy and chemotherapy.

## 2 MATERIALS AND METHODS

### 2.1 Data collection and processing

The publicly available transcriptomic cohorts for BLCA were searched. Finally, three microarray cohorts (GSE13507, GSE32894 and GSE48075) and one RNA‐sequencing cohort (TCGA‐BLCA) were enrolled in our study. Samples without complete prognosis were removed from further evaluation. All raw transcriptomic data and prognostic information from microarray cohorts were downloaded from the Gene Expression Omnibus (GEO) database (https://www.ncbi.nlm.nih.gov/geo/) (Supplementary Table 1). Subsequently, the background correction of the relative signal of each probe was processed via the RMA algorithm and log2 transformation, quantile normalization and annotation by the package ‘*Affy*’ in R.^28^ Each gene was annotated as the highest expressed probe when several probes mapped to a single gene symbol. The transcriptomic data of the TCGA‐BLCA cohort were downloaded from the TCGA Genomic Data Commons (GDC) data portal (https://portal.gdc.cancer.gov/). Each gene was annotated with the highest expression when multiple ENSEMBL IDs mapped to a single gene symbol. Then the Level 3 RNA‐sequencing data fragments Per Kilobase per Million (FPKM) values were transformed into transcripts per kilobase million (TPM) values to represent the relative expression of each gene, which makes them more comparable between samples.^29^ Detailed clinical data and sample information for the TCGA‐BLCA cohort were obtained from UCSC Xena (https://tcga.xenahubs.net) or supplementary information from Robertson et al^30^ and can be found in our previous study^31^ or Supplementary Table 2. The somatic mutation data processed with the MuTect2 algorithm from the TCGA‐BLCA cohort was obtained from the Genomic Data Commons (https://portal.gdc.cancer.gov/) using the package ‘*TCGAbiolinks*’ in R.^32^ The tumour mutation burden (TMB) per megabase of each sample was calculated according to previous study.^33^ Data were analysed with the R (version 3.5.3) and Bioconductor packages. 32 pancancer TCGA transcriptomic data and clinical information were downloaded from UCSC Xena pancancer section. We only used samples with complete outcome information to perform further analysis.

### 2.2 Establishment and validation of prognostic ATGRS

Autophagy‐related genes (ATGs) were browsed from the Human Autophagy Database (HADb, http://www.autophagy.lu/) and MSigDB of the Broad Institute (http://software.broadinstitute.org/gsea/index.jsp). Then total ATGs were submitted to LASSO cox regression analysis based on package ‘*glmnet*’ in R for establishing an optimal prognostic autophagy‐related genes signature (ATGRS) in BLCA.^34^ The formula for the ATGRS risk score was calculated as $\sum_{i=1}^{n}$ (coef_i_ × Expr_i_), where Expr_i_ is the relative expression of candidate ATG in the signature for patient i, coef_i_ is the LASSO cox coefficient of the ATG i. Furthermore, all patients were divided into high‐risk or low‐risk groups at the median cut‐off based on the ATGRS risk score for subsequent study. Kaplan‐Meier survival curves were used to detect differences in OS and DFS between ATGRS high‐/low‐risk patients and indicated stratified clinical features by using package ‘*survminer*’ in R. The prediction accuracy of ATGRS risk score in OS and DFS were measured through time‐dependent receiver operating characteristic (ROC) curves using package ‘*survivalROC*’ in R.^35^ The area under curve (AUC) for 1‐year, 3‐year and 5‐year OS and DFS was assessed. Then the correlation of ATGRS risk score with clinicopathological characteristics, molecular subtypes and somatic mutational landscape were assessed by Student *t* test or one‐way ANOVA test and depicted by box plots.

### 2.3 Construction of a predictive nomogram

The ATGRS risk score, clinicopathological characteristics and TMB were integrated to find independent prognostic factors through univariate and multivariate cox regression analysis and visualized through package ‘*forestplot*’ in R. The selected independent prognostic factors were then combined to construct a nomogram through package ‘*rms*’, ‘*nomogramEx*’ and ‘*regplot*’ in R.^36^ Furthermore, the reliability and advantage of our nomogram were measured by decision curve analysis (DCA) and calibration curves.

### 2.4 Estimation of TME immune cells infiltration

The special gene sets representing different TME immune cell types were obtained from *Bindea* et al.^37^ At last, innate TME immune cells including dendritic cells (DCs), eosinophils, mast cells, macrophages, natural killer cells (NKs) and neutrophils, as well as adaptive TME immune cells including B cells, T cells, T helper cells, regulatory T (Treg) cells and cytotoxic cells were enrolled in our study. The relative abundance of each TME immune cell type in each sample was then calculated through single‐sample gene set enrichment analysis (ssGSEA) based on the aforementioned reference gene sets.^38^

### 2.5 Chemotherapeutic and immune checkpoint Inhibitors (ICIs) immunotherapeutic response prediction

The publicly available pharmacogenomics database Genomics of Drug Sensitivity in Cancer (GDSC), (https://www.cancerrxgene.org/) was used to predict the chemotherapeutic response of each sample based on the ATGRS risk score. The half‐maximal inhibitory concentration (IC50) was estimated by ridge regression in each sample and prediction accuracy was evaluated by 10‐fold cross‐validation based on the GDSC training set through package ‘pRRophetic’ in R. All parameters were set as default values, the batch effect was removed by using the package ‘combat’ in R, the tissue type of selected as ‘allSoldTumours’.^39^ Moreover, The Tumour Immune Dysfunction and Exclusion (TIDE) algorithm was used to predict the immune checkpoint inhibitors (ICIs) targeting the immunotherapeutic response using RNA‐Seq tumour expression profiles according to the suggestion of *Hoshida Y* et al^40^

### 2.6 Pancancer validation of ATGRS

To test the usage of ATGRS in different tumour types, we used 32 other tumour types from the TCGA database. Based on the formula for ATGRS risk score, we then calculated the risk score and all patients were divided into high‐risk and low‐risk groups at the optimal cut‐off for each cohort. Kaplan‐Meier survival curves were used to detect the differences in OS between ATGRS high‐/low‐risk patients by using package ‘survminer’ in R.

### 2.7 Abnormal protein level validation for autophagy‐related genes

To further study the role of autophagy‐related genes in our prediction model, we collected the immunohistochemistry results from the human protein atlas database (https://www.proteinatlas.org/). Subsequently, we compared the staining intensity and quantity of these genes between healthy bladder and abnormal bladder cancer tissue.

### 2.8 Statistical analyses

The statistical significance of variables between two groups and more was estimated by Student *t* tests or one‐way ANOVA tests respectively. The differences in OS and DFS between different groups were shown with Kaplan‐Meier survival curves and calculated with the log‐rank test using the package ‘*survminer*’ in R. The correlation distance between two parameters was detected with spearman correlation analyses. The differences in ICIs targeting the immunotherapeutic response between different groups was evaluated with the Two‐sided Fisher's exact test. The independent prognostic factors were determined with univariate and multivariate cox proportional‐hazard models and visualized with the package ‘*forestplot*’ in R. Nomogram incorporation with independent prognostic factors, as well as the calibration curve and DCA were constructed according to *Iasonos*' suggestion with the packages ‘*rms*’, ‘*nomogramEx*’ and ‘*regplot*’ in R.^36^ The somatic mutation landscape was presented with a waterfall plot via packages ‘*maftools*’^41^ and ‘*complexheatmap*’^42^ in R. The package ‘*survivalROC*’ in R was used to plot and visualize receiver operating characteristic (ROC) curves. The area under the curve (AUC) was used to evaluate the diagnostic accuracy of parameters. All statistical analyses were performed with R software 3.5.3. Statistical significance was set at probability value of *P* < .05.

## 3 RESULTS

### 3.1 Establishment of the Autophagy‐related Genes Signature (ATGRS)

**FIGURE 1:**
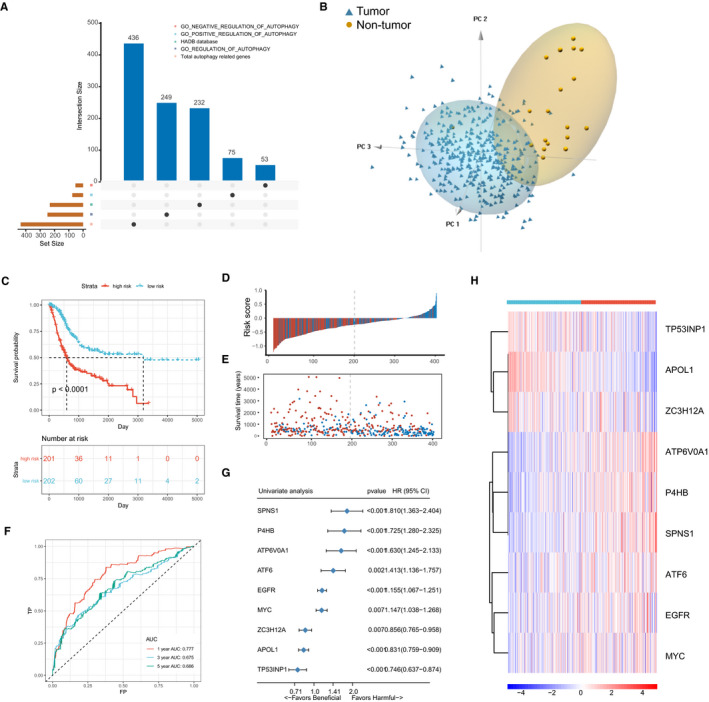
Establishment of autophagy‐related genes signature (ATGRS) in TCGA‐BLCA training cohort. A, Total candidate autophagy‐related genes (ATGs) were identified by merging the genes from HDAB and MSigDB and visualized via upset plot. B, PCA indicated that candidate ATGs were differential expressed in tumour and non‐tumour tissues. Blue triangles indicate tumour samples and brown circles indicate non‐tumour samples. C, Kaplan‐Meier survival curves show the difference in OS advantage between ATGRS high‐risk and low‐risk groups in the TCGA‐BLCA training cohort (Log‐rank test, *P* < .0001). D, Bar plot demonstrates that patients with high‐ATGRS risk scores have a higher mortality rate and patients with a low‐ATGRS risk scores were more inclined to alive. Blue bars indicate deceased patients and red bars indicate living patients. E, Dot plot indicates that survival time of high‐ATGRS risk score patients was less than patients with a low‐ATGRS risk score. Blue dots indicate the ATGRS high‐risk patients and red dots indicate ATGRS low‐risk patients. F, Time‐dependent ROC curves for ATGRS risk score in predicting OS at 1, 3, 5 years in TCGA‐BLCA training cohort. G, Forest plot summary of the univariate cox regression analyses of candidate ATGs. Blue diamond squares on the transverse, lines indicate the HR and black transverse lines indicate the 95% CI. H, Hierarchical clustering of 9 candidate ATGs for 403 patients according to the ATGRS high‐risk and low‐risk groups in TCGA‐BLCA cohort. Rows represent relative expression of each gene, and columns represent BLCA samples. Red refers to relative up‐regulated expression and blue refers to the relative down‐regulated expression of each gene. The ATGRS high‐risk and low‐risk groups were used as sample annotations

A flow diagram and design of the study can be found in Supplementary Figure 1. After merging the ATGs from the HDAB and MSigDB, we finally got 436 candidate ATGs for subsequent study (Figure 1A). We found that the 436 candidate ATGs were differentially expressed in BLCA samples and non‐tumour samples through principal components analysis (PCA) (Figure 1B). Furthermore, we submitted the 436 candidate ATGs to a LASSO cox regression analysis for dimension reduction and established an autophagy‐related genes signature (ATGRS) consisting of 9 genes in a TCGA‐BLCA training cohort. The formula for the ATGRS risk score was calculated as follows: expression of APOL1 × (−0.10598264) + expression of ATF6 × (0.03011272) + expression of ATP6V0A1 × (0.22773617) + expression of EGFR × (0.02662074) + expression of MYC × (0.04437603) + expression of P4HB × (0.18843412) + expression of SPNS1 × (0.20199096) + expression of TP53INP1 × (−0.03585352) + expression of ZC3H12A × (−0.01792276). All patients in the TCGA‐BLCA training cohort were separated into ATGRS high‐risk and low‐risk groups at the median cut‐off. The ssGSEA showed that the ATGRS high‐risk group was remarkably enriched for all autophagy‐related gene sets compared with the low‐risk group, which indicated that our established ATGRS was good at representing the autophagy status in BLCA (Supplementary Figure 2). Moreover, Kaplan‐Meier survival curves demonstrated that patients in the ATGRS high‐risk group exhibited poorer OS, while the low‐risk group was associated with a better clinical outcome in TCGA‐BLCA training cohort (Log‐rank test, *P* < .0001, Figure 1C and E). Time‐dependent ROC curves showed that ATGRS displayed a high accuracy for OS prediction in TCGA‐BLCA cohort (Figure 1F). We also found that genes ATF6, ATP6V0A1, EGFR, MYC, P4HB and SPNS1were up‐regulated in the high‐risk group, which are all harmful prognostic factors, while APOL1, TP53INP1 and ZC3H12A were up‐regulated in the low‐risk group and significantly correlated with better prognosis in BLCA (Figure 1G,H).

### 3.2 Validation of ATGRS in independent cohorts

**FIGURE 2:**
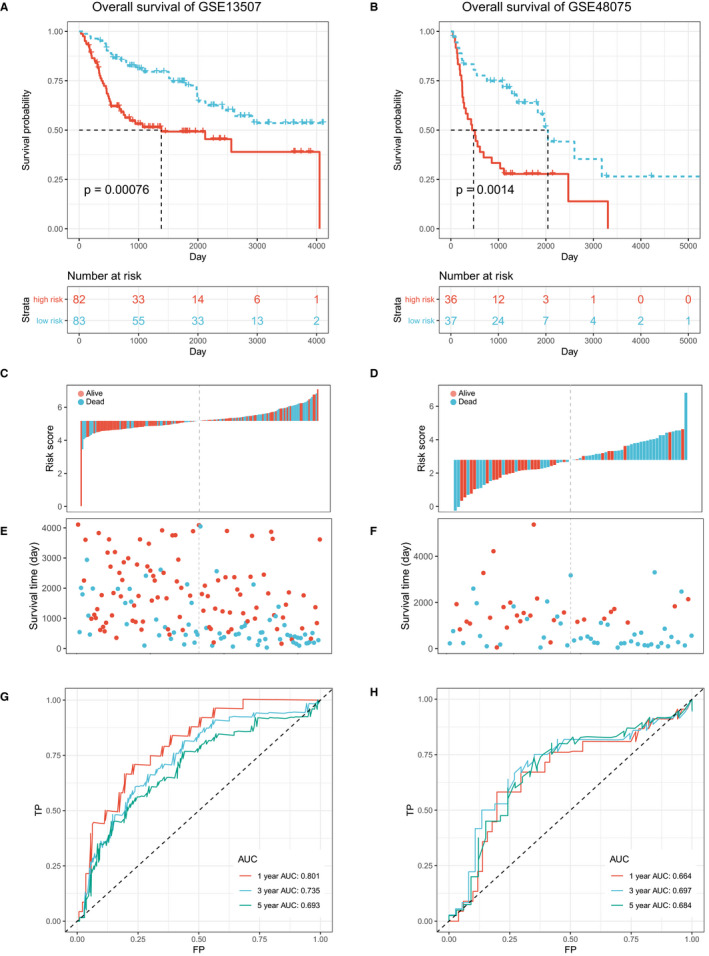
ATGRS is a prognostic biomarker for overall survival (OS) prediction. A‐B, Kaplan‐Meier survival curves show the differences in OS advantage between ATGRS high‐risk and low‐risk groups in GSE13507 (A) and GSE48075 (B) cohorts (Log‐rank test, *P* = .00076, Figure 2A; *P* = .0014, Figure 2B). C‐D, Bar plot demonstrates that patients with high‐ATGRS risk scores have a higher mortality rate and patients with a low‐ATGRS risk scores have a higher survival rate in GSE13507 (C) and GSE48075 (D) cohorts. Blue indicates deceased patients and red indicates surviving patients. E‐F, Dot plot indicating that survival time of high‐ATGRS risk score patients is decreased compared to low‐ATGRS risk score patients in GSE13507 (E) and GSE48075 (F) cohorts. Blue indicates the ATGRS high‐risk patients and red indicates ATGRS low‐risk patients. G‐H, Time‐dependent ROC curves for ATGRS risk score in predicting OS at 1, 3, 5 y in GSE13507 (G) and GSE48075 (H) cohorts

**FIGURE 3:**
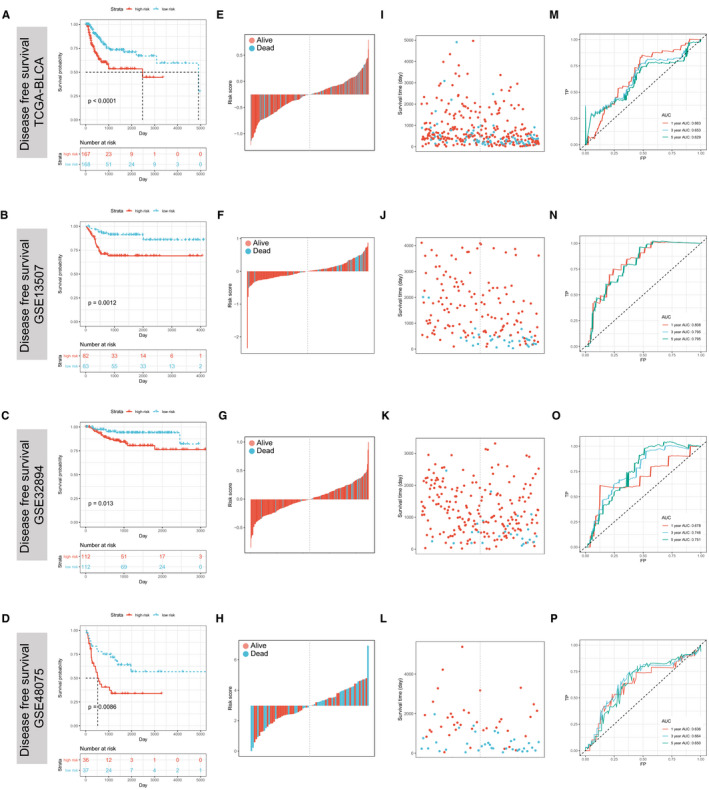
ATGRS is a prognostic biomarker for disease‐free survival (DFS) prediction. A‐D, Kaplan‐Meier survival curves show differences in DFS advantage between ATGRS high‐risk and low‐risk groups in TCGA‐BLCA (A), GSE13507 (B), GSE32894 (C) and GSE48075 (D) cohorts (Log‐rank test, *P* < .0001, Figure 3A; *P* = .0012, Figure 3B, *P* = .013, Figure 3C; *P* = .0086, Figure 3D). E‐H, Bar plot demonstrates that patients with high‐ATGRS risk scores have a higher mortality rate and patients with a low‐ATGRS risk score patients have a higher survival rate in TCGA‐BLCA (E), GSE13507 (F), GSE32894 (G) and GSE48075 (H) cohorts. Blue bars indicate deceased patients and red bars indicate surviving patients. I‐L, Dot plot indicating that survival time of high‐ATGRS risk score patients is decreased compared to low‐ATGRS risk score patients in TCGA‐BLCA (I), GSE13507 (J), GSE32894 (K) and GSE48075 (L) cohorts. Blue dots indicate the ATGRS high‐risk patients and red dots indicate ATGRS low‐risk patients. M‐P, Time‐dependent ROC curves for ATGRS risk score in prediction DFS at 1, 3, 5 y in TCGA‐BLCA (M), GSE13507 (N), GSE32894 (O) and GSE48075 (P) cohorts

To further assess the clinical utility of ATGRS, its performance was assessed in several independent cohorts, including 2 external cohorts (GSE13507 and GSE48075) for OS validation, as well as 1 internal cohort (TCGA‐BLCA) and 3 external cohorts (GSE13507, GSE32548 and GSE48075) for DFS validation. Fortunately, the performance of ATGRS worked well in all validation cohorts showing that all patients with a high‐ATGRS risk score were associated with a poorer OS (Log‐rank test, GSE13507, *P* = .00076, Figure 2A and GSE48075 cohort, *P* = .0014, Figure 2B) and DFS (Log‐rank test, TCGA‐BLCA, *P* < .0001, Figure 3A; GSE13507, *P* = .0012, Figure 3B; GSE32894, *P* = .013, Figure 3C; GSE48075, *P* = .0086, Figure 3D) when compared with low‐risk groups. The high‐risk ATGRS patient group had an increased mortality with a poorer OS and DFS outcome), while in the ATGRS low‐risk group a lower mortality and longer survival was observed(GSE13507, Figure 2C,E; GSE48075 cohort, Figure 2D,F; TCGA‐BLCA, Figure 3E,I; GSE13507, Figure 3F,J; GSE32894, Figure 3G,K; and GSE48075, Figure 3H,L). Consistent with the outcome of the TCGA‐BLCA training cohort, we found that the ATGRS risk score also exhibited a high accuracy in OS and DFS prediction in each cohort (GSE13507, Figure 2G; GSE48075 cohort, Figure 2H; TCGA‐BLCA, Figure 3M; GSE13507, Figure 3N; GSE32894, Figure 3O and GSE48075, Figure 3P). Moreover, we compared our ATGRS with a different autophagy model constructed by Wang et al in BLCA.^43^ Kaplan‐Meier survival curves showed that Wang's model could only be used for OS prediction in the TCGA‐BLCA cohort (Log‐rank test, *P* = .0025, Supplementary Figure 3A) and the DFS prediction in GSE13507 cohort (Log‐rank test, *P* = .027, Supplementary Figure 3B), but was not suitable for any other cohort in prediction of OS (Log‐rank test, GSE13507, *P* = .076 and GSE48075 cohort, *P* = .0014, Supplementary Figure 3A) and DFS (Log‐rank test, TCGA‐BLCA, *P* = .15; GSE32894, *P* = .1; GSE48075, Log‐rank test, *P* = .25, Supplementary Figure 3B). Moreover, time‐dependent ROC indicated that our ATGRS was significantly superior to that of Wang's model showing that all AUCs were remarkably higher in ATGRS when compared with their model (Supplementary Figure 3C).

### 3.3 Correlations between ATGRS and molecular classification as well as clinicopathological characteristics

**FIGURE 4:**
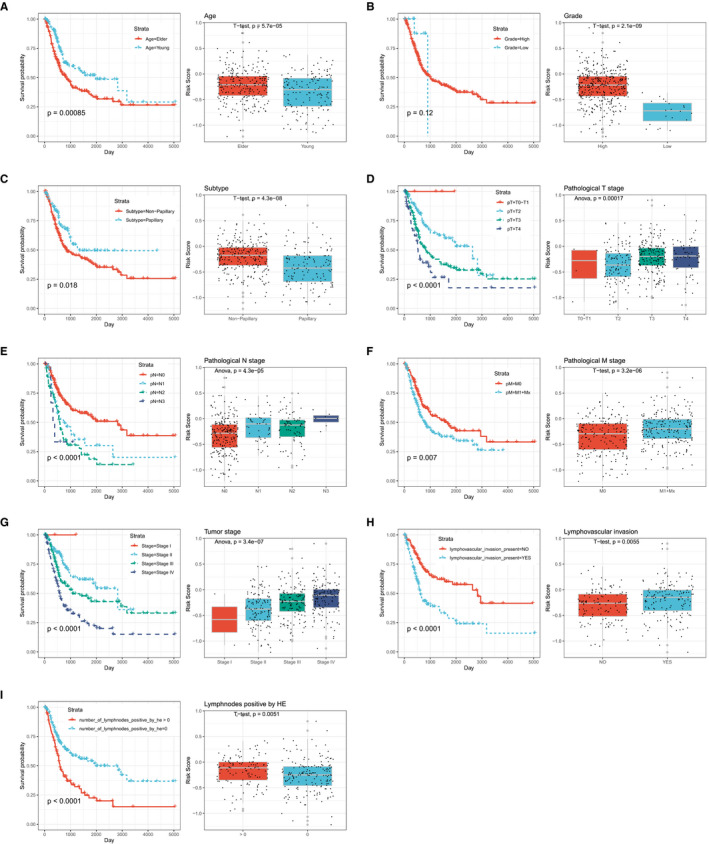
Association between ATGRS and clinicopathological characteristics. The survival rate of depicted subgroups in different clinicopathological characteristics was measured. Boxplots indicate the correlation between the ATGRS risk score and the subtype of each clinicopathological characteristic by *t* test or one‐way ANOVA test. The patients were stratified into different subtypes based on (A) age: elder: age>65, younger: age≤65; (B) tumour grade: high and low; (C) histological subtype: non‐papillary and papillary; (D) pathological T stage: T0 + T1 stage, T2 stage, T3 stage and T4 stage; (E) pathological N stage: N0 stage, N1 stage, N2 stage and N3 stage; (F) pathological M stage: M0 stage, M1+Mx stage; (G) pathological tumour stage: stage I, stage II, stage III and stage IV; (H) lymphovascular invasion status: lymphovascular invasion ‐ and lymphovascular invasion +; (I) number of positive lymph nodes by HE: number of positive lymph nodes by HE = 0 and number of positive lymphnodes by HE > 0

Recently, distinct molecular subtype classification systems based on genomic characterization were comprehensively investigated in multi‐platforms by The Cancer Genome Atlas (TCGA) Network and other independent cohorts in BLCA. Compared with traditional anatomic tumour staging and grade, molecular subtypes classify a tumour closer to the native biology of BLCA. So far, several classification systems have been widely accept and described.^44^, ^45^, ^46^, ^47^, ^48^ We found that distinct molecular subtypes established by different groups based on special criteria and algorithm, exhibited unique characteristics and overlap between each other. All of this further demonstrated the extreme complexity of BLCA. Subsequently, we detected the association between ATGRS and molecular subtypes. The samples within TCGA‐BLCA cohort were dichotomized into ATGRS high‐risk and low‐risk groups at median cut‐off. Surprisingly, we found that patients with a high‐ATGRS risk score were more likely to have a molecular subtypes associated with high malignancy and poorer prognosis including basal, basal squamous, TP53‐like, TCGA III/IV, CC1/3 and basal/SCClike. However, luminal, luminal papillary, TCGA I/II, CC2 and uroA molecular subtypes, characterized by low malignancy and better survival, were significantly accumulated in the low‐ATGRS risk score group (Supplementary Figure 4A‐G). Furthermore, the correlation of ATGRS with clinicopathological characteristics was further assessed. The boxplots showed that the ATGRS risk score remarkably increased in patients with elderly, non‐papillary, lymphovascular and lympho‐nodes invasion, and higher pathological stages and grades, while the patients with a low‐ATGRS risk score displayed the opposite distribution (Figure 4A‐I). Due to the strong association between the above clinical parameters, we tried to decipher whether ATGRS was responsible for prognosis independency of these clinicopathological characteristics. We divided all patients into subgroups showed in Supplementary Figure 5. Stratification survival analyses revealed that the ATGRS risk score could efficiently predict the prognosis of BLCA patients in practically all subgroups from the aforementioned clinical features (Supplementary Figure 5A‐R).

### 3.4 ATGRS is highly negative correlated with TMB

**FIGURE 5:**
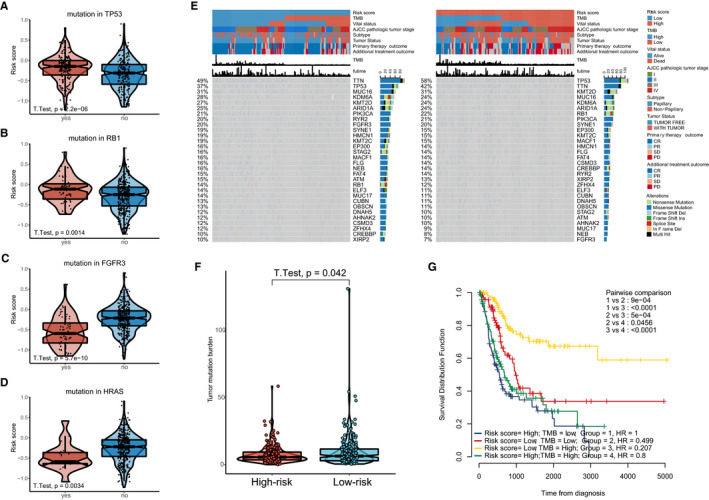
Association between ATGRS and somatic genetic alteration. A‐D, Differences in ATGRS risk score between mutation and wild‐type TP53 (A), RB1 (B), FGFR3 (C) and H‐RAS (D). The upper and lower ends of the boxes represent the interquartile range of values. The lines in the boxes represent the median value. Students *t* test was used to compare the statistical differences. E, The waterfall plot of tumour somatic mutation displays the distribution of the top 30 highly variant mutated genes that correlate with ATGRS. The mutational type includes frame shift del, frame shift ins, in frame del, in frame ins, missense mutation, multi hit, nonsense mutation and splice site. Each column represents individual patient. The upper bar plots show the ATGRS risk score, TMB and OStime, The number on the left indicates the mutation frequency in each gene. The right bar plot shows the proportion of each variant type. The ATGRS, TMB, vital status, AJCC pathological tumour stage, subtypes, primary therapy outcome and additional treatment outcome were used as patient annotations. F, Box plot showing the correlation of TMB with ATGRS (Students *t* test, *P* = .042). G, Kaplan‐Meier curves for four groups stratified by combining ATGRS with TMB in TCGA‐BLCA cohort. Log‐rank test shows a significant survival difference among different groups

Given that BLCA is a disease characterized with high‐somatic genetic alterations, we next investigated whether there was a correlation between the ATGRS and mutation alterations. As a result, we found that patients with a mutation in TP53 and RB1, which is often mutated in a later stage of BLCA (MIBC) and recognized as a key element in BLCA development, exhibited higher ATGRS risk scores compared with the ATGRS low‐risk group (Figure 5A,B and E). Moreover, mutations in FGFR3 and H‐RAS, which appear in an early stage of BLCA (NMIBC), were more likely to occur in ATGRS low‐risk patients (Figure 5C,D and E). Above findings showed that our ATGRS profile was positive with the tumour malignancy from a different point of view. Furthermore, the waterfall plot revealed that the mutation load was increased in the ATGRS low‐risk group (Figure 5E) and a negative correlation between the ATGRS and TMB (Figure 5F) was observed. All the patients were then equally stratified into four groups based on ATGRS and TMB at median cut‐offs. Kaplan‐Meier survival curves showed that patients with a high‐TMB/low‐ATGRS risk score displayed the highest survival advantage, while patients with a low‐TMB/high‐ATGRS risk score had prognosed the worst outcome. As TMB was robustly negative to the clinical response of immunotherapy, we inferred that the ATGRS could also be valuable for predicting immunotherapy response.

### 3.5 The ATGRS is an independent prognostic factor in BLCA

**FIGURE 6:**
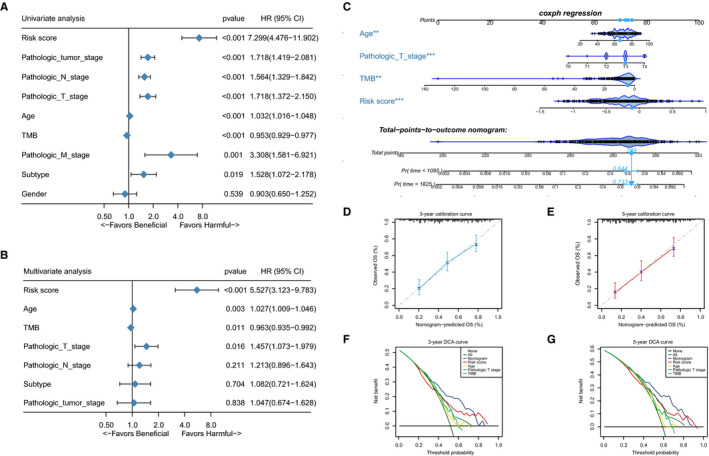
ATGRS is an independent prognosis factor in the nomogram. A‐B, Forest plot summary of the univariate (A) and multivariable (B) cox analyses of the ATGRS risk score, clinicopathological characteristics and TMB. The results indicate four independent prognosis factors which include age, pathologic T stage, TMB and ATGRS risk score. The blue diamond on the transverse lines represent the HR, and the black transverse lines represent the 95% CI. The p value and 95% CI for each clinical feature are displayed in detail. C, Nomograms predicting the probability of patient mortality at 3‐ or 5‐y OS based on four independent prognosis factors. D‐E, Calibration curves of the nomogram for predicting probability of OS at 3, and 5 y. F‐G, Decision curve analyses (DCA) of the nomograms based on four independent prognosis factors for 3‐y and 5‐y

Next, we combined the ATGRS risk score, clinicopathological characteristics and TMB to determine whether the ATGRS risk score is an independent prognostic factor in BLCA. The univariate cox regression analysis showed that ATGRS risk score was a significantly harmful prognostic factor for BLCA (Figure 6A). The multivariate cox regression analysis revealed that the ATGRS risk score, TMB, age and pathological T stage were independent factors in predicting prognosis of BLCA patients (Figure 6B). Furthermore, the scoring system nomogram was established combining the four independent prognostic factors (Figure 6C). The total scoring points of each patient in the nomogram was calculated by adding the points for each clinical parameter. A higher number indicates a worse prognosis. We observed that every patient will get a total point by plus every point of each clinical parameter in nomogram. The patients with higher total points will get a worse prognosis. Calibration curves revealed that the accuracy of our established nomogram in predicting OS was similar to the ideal model (Figure 6D,E). DCA curves showed that the nomogram exhibited more net benefit compared with ATGRS risk score and other independent prognostic factors alone and was highly potent for clinical utility (Figure 6F,G).

### 3.6 ATGRS could predict the clinical response of immunotherapy and chemotherapy

**FIGURE 7:**
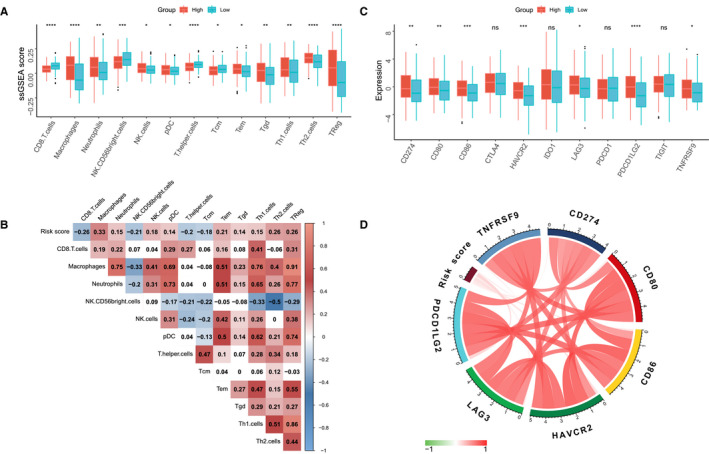
ATGRS is associated with TME immune cells infiltration. A, Differences in infiltration of TME immune cells between high‐ and low‐risk ATGRS groups. The upper and lower ends of the boxes represent interquartile range of values. Boxlines represent median values and black dots show outliers. Statistical difference was tested by students *t* test and statistical differences in immune cell amounts were shown in Figure 7A‐B. The asterisks represent statistical p value (*P < .05; **P < .01; ***P < .001; ****P < .0001). B, Correlation between ATGRS risk score and infiltration of TME immune cells. Negative correlation is marked in blue and positive correlation is marked in red. C, Differences in expression of immune checkpoints between high‐ and low‐risk ATGRS groups. The upper and lower ends of the boxes represent interquartile range of values. Boxlines represent median values and black dots show outliers. The statistical difference was tested by the students *t* test. The asterisks represent the statistical p value (ns no significance; *P < .05; **P < .01; ***P < .001; ****P < .0001). D, Correlation chord chart shows the mutual correlation between ATGRS risk score and several prominent immune checkpoint‐relevant genes

**FIGURE 8:**
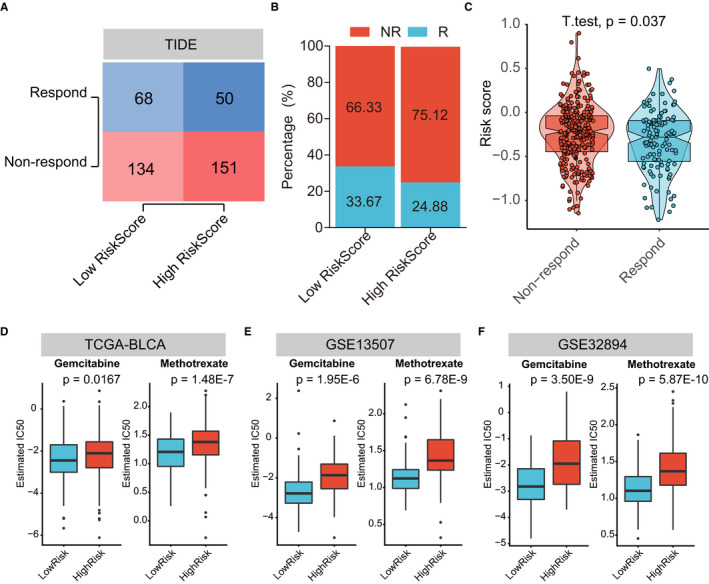
ATGRS are efficient in predicting the immunotherapeutic and chemotherapeutic benefit in BLCA. A, Number of patients with response to immunotherapy between low‐ and high‐ATGRS groups in TCGA‐BLCA cohort. The size of the colourshade represents the number. Red indicate responders; blue represents non‐responders. Two‐sided Fisher's exact tests were used to analyse contingency tables for ICIs responders (*P* = .0626). B, The proportion of patients which respond to immunotherapy between low‐ and high‐ATGRS groups in TCGA‐BLCA cohort. Red indicate responders; blue represents non‐responders. C, The distribution of immunotherapeutic response between high‐ and low‐risk groups stratified by ATGRS risk score in TCGA‐BLCA cohort based on the TIDE algorithm (students *t* test, *P* = .037). D‐F, Differential putative chemotherapeutic response. The box plots show differences in the estimated IC50 for gemcitabine and methotrexate between ATGRS high‐risk and low‐risk groups in TCGA‐BLCA (D), GSE13507 (E) and GSE32894 (F) cohorts

Nowadays, tumour microenvironment (TME) was identified as an essential element in tumorigenesis and has been recognized as a new target for tumour therapy. Many clinical trials demonstrated that TME immune cells infiltration in situ is an essential factor in predicting clinical outcome for advance cancer patients receiving ICIs.^49^, ^50^ We comprehensively evaluated the TME immune cells infiltration landscape through the ssGSEA algorithm and the association with ATGRS was measured. Strikingly we found that BLCA tumours with the ATGRS low‐risk profile were infiltrated with effector immune cells including CD8+ T cells and NK CD56bright cells, while tumours from the ATGRS high‐risk group were filled with immunosuppressive cells such as Treg and macrophages (Figure 7A, Supplementary Table 3, and Supplementary Figure 6). Moreover, spearman correlation analyses showed that the ATGRS risk score significantly negative correlated with CD8+ T cells and NK CD56bright cells and positive correlated with Treg and macrophages (Figure 7B). We postulate that expression of immune checkpoints causes the current TME immune cells infiltration status. Therefore, immune checkpoints, such as CD274 (PD‐L1), CD80, CD86, CTLA4, HAVCR2 (TIM‐3), IDO1, LAG3, PDCD1 (PD‐1), PDCD1LG2 (PD‐L2), TIGHT, TNFRSF9 were selected. We found that nearly all immune checkpoints (CD274, CD80, CD86, HAVCR2, LAG‐3, PDCD1LG2 and TNFRSF9) were up‐regulated in the ATGRS high‐risk group when compared with the ATGRS low‐risk group (Figure 7C). Moreover, the correlation matrix in the chord chart revealed that the ATGRS risk score exhibited a significant positive correlation with the above mentioned immune checkpoint relevant genes (Figure 7D). We introduced the TIDE algorithm to predict the likelihood of response to ICIs by utilizing transcriptomic data. The detailed output of TIDE can be found in Supplementary Table 4. The results showed that the number and percentage of responders to ICIs was significantly higher in the ATGRS low‐risk group (68/202, 33.67%) than the ATGRS high‐risk group (50/201, 24.88%) (two‐sided Fisher's exact test, *P* = .0626, Figure 8A,B). Moreover, the non‐responders were more likely to be patients with higher ATGRS risk score while responders displayed a lower ATGRS risk score (Figure 8C). Subsequently, we assessed the differences in chemotherapy response between the high‐ and low‐ATGRS groups. A predictive model on the GDSC cell line data set by ridge regression was trained and a predictive accuracy was evaluated by 10‐fold cross‐validation. Then we estimated the IC50 for each patient in three independent cohorts including TCGA‐BLCA, GSE13507 and GSE32894 based on the predictive model of gemcitabine and methotrexate. A significant difference was observed in the estimated IC50 between ATGRS high‐risk and low‐risk groups. For these two chemo drugs, patients with low‐risk ATGRS were more sensitive to commonly administered chemotherapies (Figure 8D,F) then high‐risk ATGRS patients.

### 3.7 The ATGRS behaves differently in different cancer types

To further assess the utility use of ATGRS in different cancer types, 32 different cancer types from the TCGA database were analysed. In 12 out of 32 cancer types we observed that the ATGRS can be used as an independent factor for prognosis. In the additional cancer types, ATGRS was not able to distinguish a specific cluster of patients with higher/lower survival rate (Supplementary Figures 7 and 8). The ATGRS profile could be a risk factor in cancer types as ACC, CESC, HNSC, MESO, SARC, SKCM and THCA, while in CHOL, LGG, LUAD, PAAD and READ ATGRS profile can be used as a beneficial marker.

### 3.8 Abnormal expression of autophagy‐related genes between normal bladder and bladder cancer tissues

Although based on limited normal bladder tissues from the human protein atlas database, and not able to draw strong conclusions, we still observed trends that autophagy‐related genes in our prediction model show dysregulation between normal bladder and bladder cancer tissues (Supplementary Figure [Link], [Link], [Link], [Link], [Link], [Link], [Link], [Link], [Link]). Tumour suppressor genes expression levels (APOL1, TP53INP1 and ZC3H12A) were low in BLCA tissues comparing with normal bladder. The other oncogenes (ATP6V0A1, P4HB, SPNS1, ATF6, EGFR and MYC) showed higher expression levels in BLCA tissues than normal bladder.

## 4 DISCUSSION

Currently, many non‐invasive biomarkers including UroVysion fluorescent in situ hybridization (FISH), and nuclear matrix protein 22 (NMP‐22), etc have been approved by the Food and Drug Administration (FDA), but their unsatisfactory specificity and sensitivity could not replace the golden standard of pathological examination after cystoscopy.^51^ Emerging ICIs targeting immunotherapy exhibited significant success in the treatment of diverse cancers including advanced BLCA, taking into account that several limitations also exist, including low‐response rate, subsequent acquired resistance and severe side effects, which can lead to unfavourable outcomes.^52^ Furthermore, some research groups reported that traditional chemotherapy and/or radiotherapy could affect the TME atmosphere in situ and induce synergistic effects with ICIs, resulting in improvement in efficiency of immunotherapies, as well as reducing side effects,^53^ but definite clinical outcome advantage had to be identified by clinical trials.

Autophagy is a biological phenomenon associated with lysosomal degradation, which is widely occurring in eukaryotic cells. Under certain stress conditions, autophagosomes are formed by swallowing cytoplasmic organelles and proteins through double‐membrane autophagic vesicles.^54^ Fusion of the autophagosome and lysosome results in the formation of the autolysosome, which provides an acidic environment allowing hydrolytic enzymes to degrade the internalized cellular components.^55^ A fascinating process which is regulated by multiple autophagy‐related proteins, for example beclin‐1, ATG‐8, LC3, etc, as well as diverse signalling pathways, such as the mTOR signalling pathway, PI3K‐AKT signalling pathway, p53 signalling pathway, etc.^56^, ^57^, ^58^ These complex regulatory networks demonstrate a controversial role of autophagy in tumorigenesis. On one hand, inhibition of autophagy is potent to increase the sensitivity of cancer cells to treatment including chemotherapy and/or radiotherapy, a process known as protective autophagy. On the other hand, autophagy over‐activation might result in autophagic cell death, similar to apoptosis.^59^ Therefore, as a potentially novel therapeutic target, it is crucial to determine the role of autophagy and understand the mechanism and process of autophagy in distinct cancer cells so that we can develop novel therapeutic agents associated with autophagy in the treatment of cancer.

In the present study, we comprehensively gathered the total autophagy‐related genes (ATGs) and found that they are differentially expressed in tumour and pancancer samples, indicating the role of autophagy in the development of BLCA. Subsequently, we identified several prognostic candidate ATGs to establish an autophagy‐related genes signature (ATGRS), which could reflect the autophagy status in BLCA. A potential candidate ATG, epidermal growth factor receptor (EGFR), binds and leads to the multisite tyrosine phosphorylation of Beclin 1, decreasing Beclin 1‐associated VPS34 kinase activity. Furthermore, EGFR tyrosine kinase inhibitor (TKI) therapy could disrupt Beclin 1 tyrosine phosphorylation and restores autophagy in non‐small‐cell lung carcinoma (NSCLC) cells.^60^ Tumour protein 53‐induced nuclear protein 1 (TP53INP1) is a tumour suppressor, whose expression is down‐regulated in diverse types of cancers. TP53INP1 is able to interact with ATG8‐family proteins and can induce caspase‐dependent autophagy, leading to cell death.^61^ We also observed that SPNS1 and ATP6V0A1 made a great contribution to our model. SPNS1 is a putative lysosomal H+‐carbohydrate transporter, which could induce aberrant autolysosome formation at a late stage of autophagy.^62^ P4HB is an ATG, which is significantly increased in clear cell renal cell carcinoma (ccRCC) at the mRNA and protein level, showing a high ability of diagnosis and prognosis.^63^ However, the specific mechanism of P4HB in regulation in autophagy is rarely known and is worthy of further study.

In this study, we equally divided all patients into ATGRS high‐risk and low‐risk groups in several independent cohorts. Kaplan‐Meier survival curves showed that high‐ATGRS risk score patients were strikingly correlated with poorer OS and DFS while the low‐risk group was associated with better prognosis. Moreover, ATGRS was significantly superior in predicting the OS and DFS with good reproducibility and high accuracy compared with previous Wang's model. Consistent with the prognosis prediction, we found that ATGRS risk score was strongly correlated with high‐malignancy molecular subtypes including basal, basal squamous, TP53‐like, TCGA III/IV, CC1/3 and basal/SCC like, which were characterized by poor survival. Moreover, patients with clinicopathological characteristics such as elderly, non‐papillary, lymphovascular and lympho‐nodes invasion, and higher pathological stages and grades were also more likely to have a higher ATGRS risk score. Furthermore, stratification survival analyses showed that ATGRS could clearly distinguish patients from all subgroups of aforementioned clinicopathological characteristics, demonstrating that ATGRS was responsible for prognosis independent of them. Additionally, there is a clear etiological link between mutations in genes that control autophagy and human disease, especially in neurodegenerative, inflammatory disorders and cancer.^64^ Somatic mutations are common in BLCA; therefore, we questioned if ATGRS was correlated with mutation alteration in BLCA. TP53 and RB1, which are always mutated in MIBC, were highly altered in ATGRS high‐risk patients, while FGFR3 and H‐RAS, which are always mutated in NMIBC, were highly altered in ATGRS low‐risk patients. Moreover, patients with low‐risk ATGRS exhibited higher TMB than high‐risk patients, indicating the negative correlation between ATGRS and TMB. We also observed that a combined strategy analyses could stratify patients more accurately than ATGRS and TMB alone. Furthermore, ATGRS remained as an independent prognostic factor and its contribution to the nomogram was strengthened by a combination of clinicopathological characteristics and TMB. These findings suggest that ATGRS might represent the protective autophagy status which can serve as an oncogenic prognostic predictor in BLCA.

Currently, autophagy is reported to contribute to trained immunity induced by Bacillus Calmette‐Guérin (BCG), which is the most widely used vaccine in NMIBC patients. Moreover, pharmacologic inhibition of autophagy could block trained immunity induced by BCG.^65^ In addition, Zhu et al reported that ATG7 overexpression elevates PD‐L1 protein levels through promoting autophagy‐mediated degradation of FOXO3a, thereby inhibiting its initiated miR‐145 transcription. A lower expression of miR‐145 increases pd‐l1 mRNA stability due to the reduction of its direct binding to 3'‐UTR of pd‐l1 mRNA, subsequently leading to increased pd‐l1 mRNA expression, and finally enhancing stem‐like property and invasion of BLCA cells.^66^ These findings indicate the significant association between autophagy and TME immune infiltration, which plays a vital role in tumorigenesis and now has been recognized as indicator for predicting clinical outcome of immunotherapy with ICIs.^49^, ^50^ After a comprehensive analysis of TME immune infiltration landscape, we found that effector immune cells including CD8+ T cells and NK CD56bright cells were highly infiltrated in the ATGRS low‐risk group, while the ATGRS high‐risk group was elevated with immunosuppressive cells such as Treg and macrophages. Moreover, ATGRS risk score was found to be significantly positive correlated with the expression of immune checkpoints, which act as rheostat in immune response regulation by inhibiting the priming of effector immune cells and immune surveillance.^67^, ^68^ All of these indicates the poor prognosis of patients with a high‐risk ATGRS that might be induced by up‐regulation of immune checkpoints and formation of immunosuppressive TME.

We have noticed that ATGRS was remarkably positive correlated with immunosuppressive TME and negatively correlated with TMB, both of which were responsible for prediction of the clinical response to ICIs targeting immunotherapy. With the use of the TIDE algorithm, ATGRS was proved to be efficient in predicting the likelihood of response to ICIs immunotherapy in BLCA. Therefore, ATGRS was negative correlated with the immunotherapy response and there were more immunotherapeutic responders in ATGRS low‐risk groups (68/202, 33.67%) than high‐risk groups (50/201, 24.88%), indicating that ATGRS could be a potent biomarker for predicting the immunotherapy response. Considering that chemotherapy is the routine way in treatment of BLCA, we introduced the GDSC database to assess the role of ATGRS in chemotherapeutic response prediction. We found that the IC50 of gemcitabine and methotrexate in ATGRS high‐risk patients were significantly higher than that in ATGRS low‐risk patients. These findings may help us to interpret the underlying mechanism and process of autophagy and sheds a light on the clinical application for a combination of autophagy modification with targeted immunotherapies and chemotherapy for BLCA.

## 5 CONCLUSIONS

We have established and comprehensive analysed a prognostic autophagy‐related genes signature (ATGRS), which could efficiently predict the response to ICIs immunotherapy and chemotherapy in BLCA, which has broaden our view on an autophagy modification strategy and may provide a useful scoring system for clinical utilities.

## CONFLICT OF INTEREST

Authors declare that they have no conflict of interest.

## AUTHOR CONTRIBUTION

**Rui Cao:** Conceptualization (equal); Data curation (equal); Formal analysis (equal); Funding acquisition (supporting); Software (equal); Writing‐original draft (equal). **Bo Ma:** Conceptualization (equal); Data curation (equal); Investigation (equal). **Gang Wang:** Conceptualization (equal); Data curation (equal); Investigation (equal). **Yaoyi Xiong:** Investigation (equal). **Ye** **Tian:** Supervision (equal); Writing‐review & editing (equal). **Lushun Yuan:** Data curation (equal); Formal analysis (equal); Methodology (equal); Software (equal); Writing‐original draft (equal); Writing‐review & editing (equal).

## ETHICAL APPROVAL

Since this was a retrospective study and all the data were collected from a public database TCGA, GEO or GDSC database, therefore, ethical approval was not required.

## INFORMED CONSENT

All the analysed data used in our research were collected from a public database, such as TCGA, GEO and GDSC; therefore, informed consent was not required for this analysis.

## CONSENT FOR PUBLICATION

All authors read and approved the final manuscript.

## Supporting information

Fig S1

Fig S2

Fig S3

Fig S4

Fig S5

Fig S6

Fig S7

Fig S8

Fig S9

Fig S10

Fig S11

Fig S12

Fig S13

Fig S14

Fig S15

Fig S16

Fig S17

Table S1

Table S2

Table S3

Table S4

Supplementary Material

## Data Availability

All data generated or analysed during this study are included in this published article and its Additional files.
