# Supplementary figures and images for: Identification of autophagy‐related genes signature predicts chemotherapeutic and immunotherapeutic efficiency in bladder cancer (BLCA)
Source: J Cell Mol Med. 2021 May 7;25(12):5417–33. doi: 10.1111/jcmm.16552 (PMC8184684; doi:10.1111/jcmm.16552)

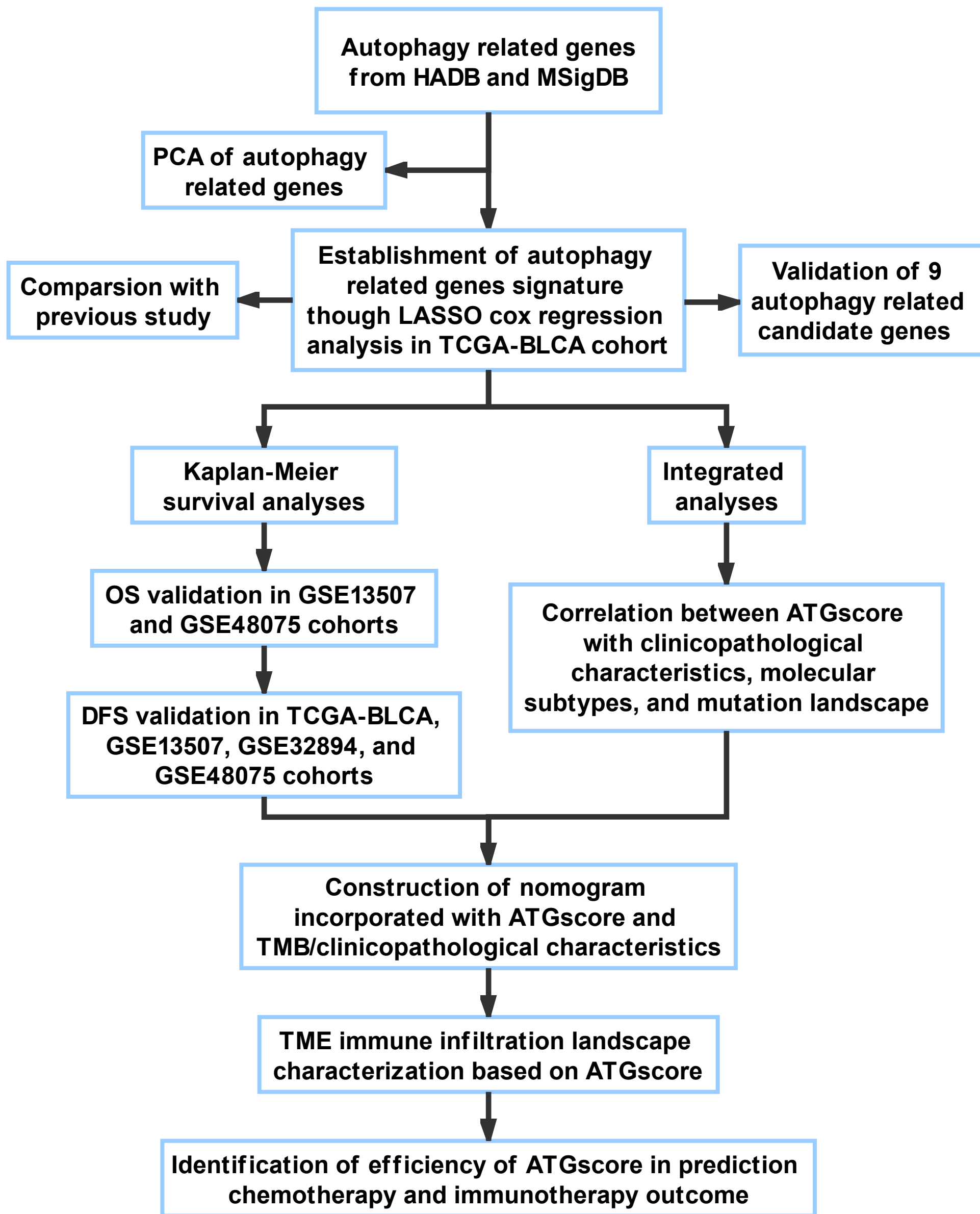

Supplement: Supplementary file 1 — Fig S1 [file JCMM-25-5417-s019.pdf]

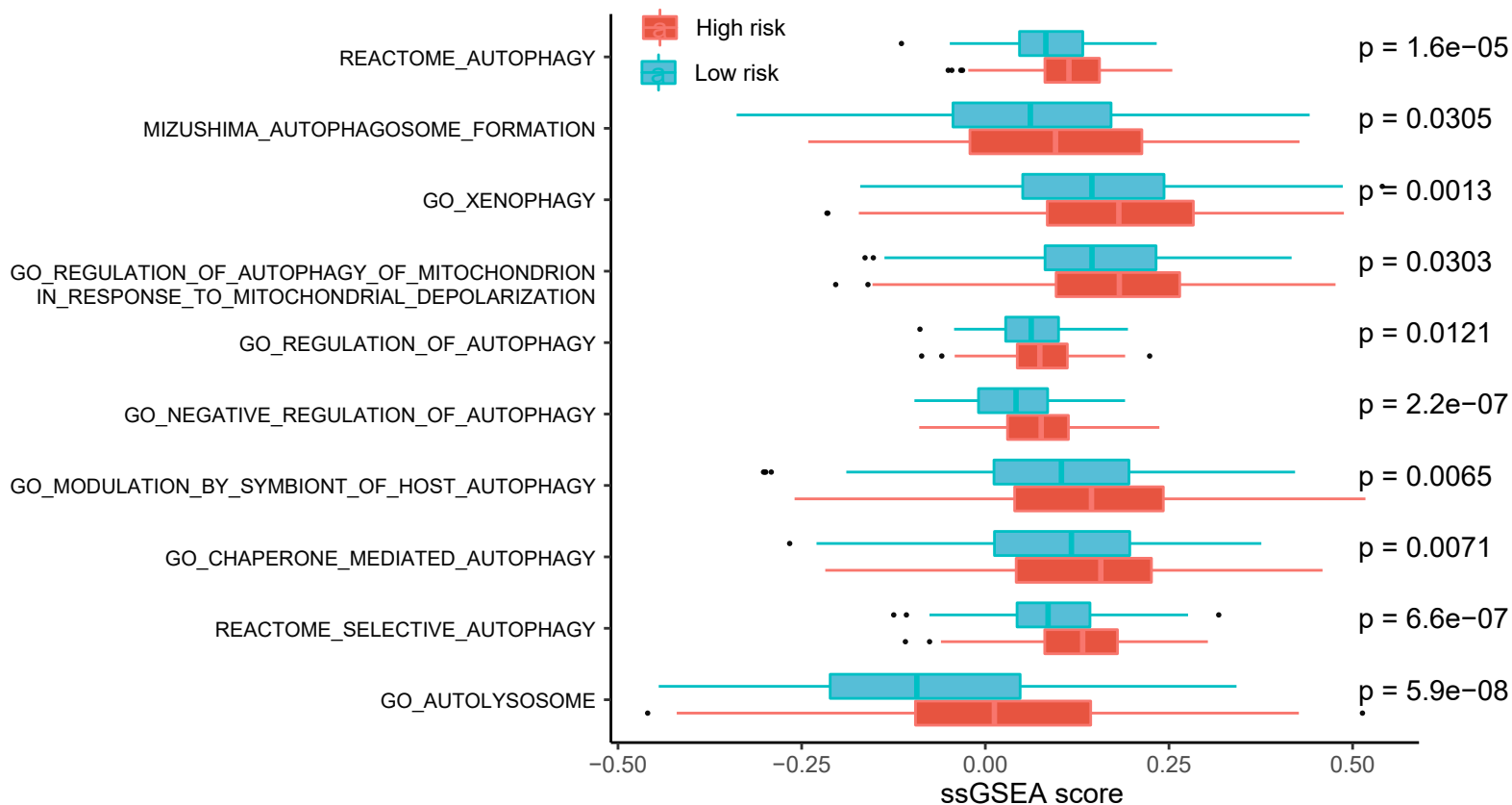

Supplement: Supplementary file 2 — Fig S2 [file JCMM-25-5417-s015.pdf]

A

Wang et al.

Overall survival

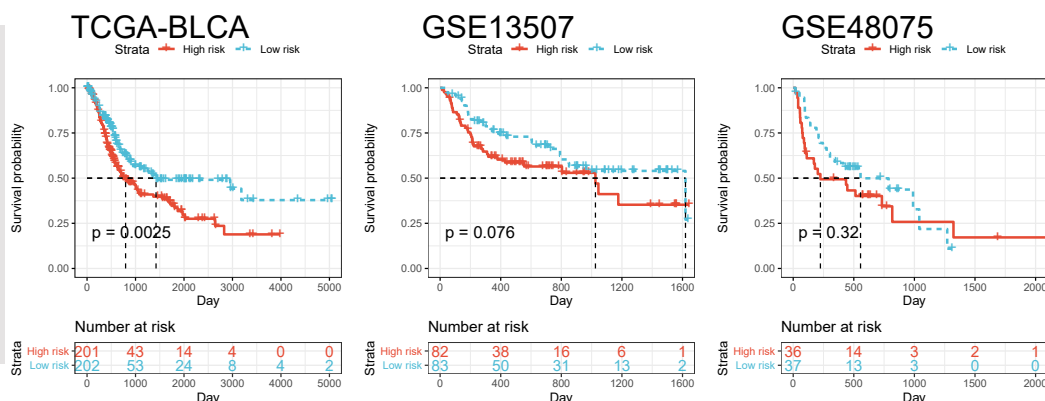

B

Wang et al.

Disease free survival

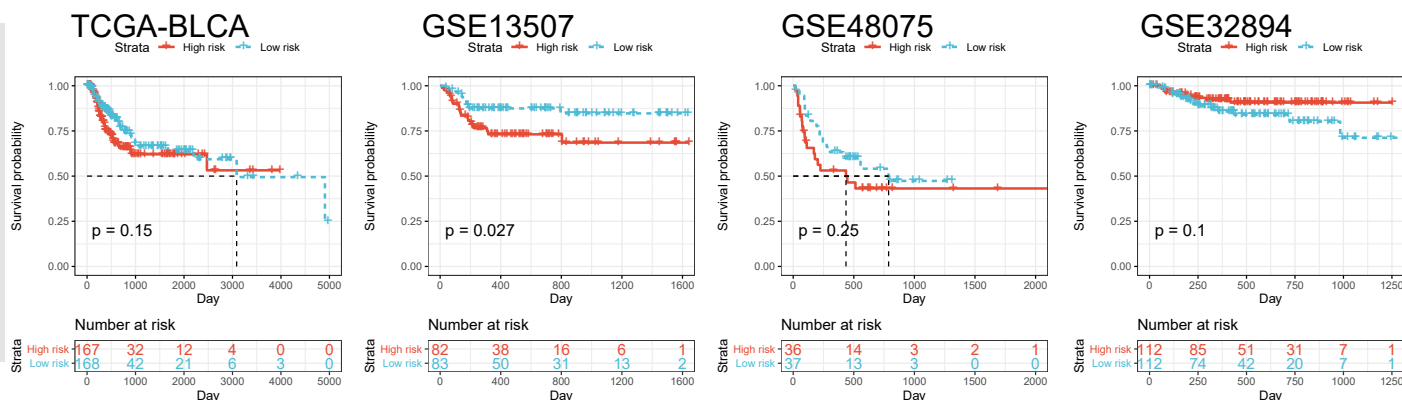

C

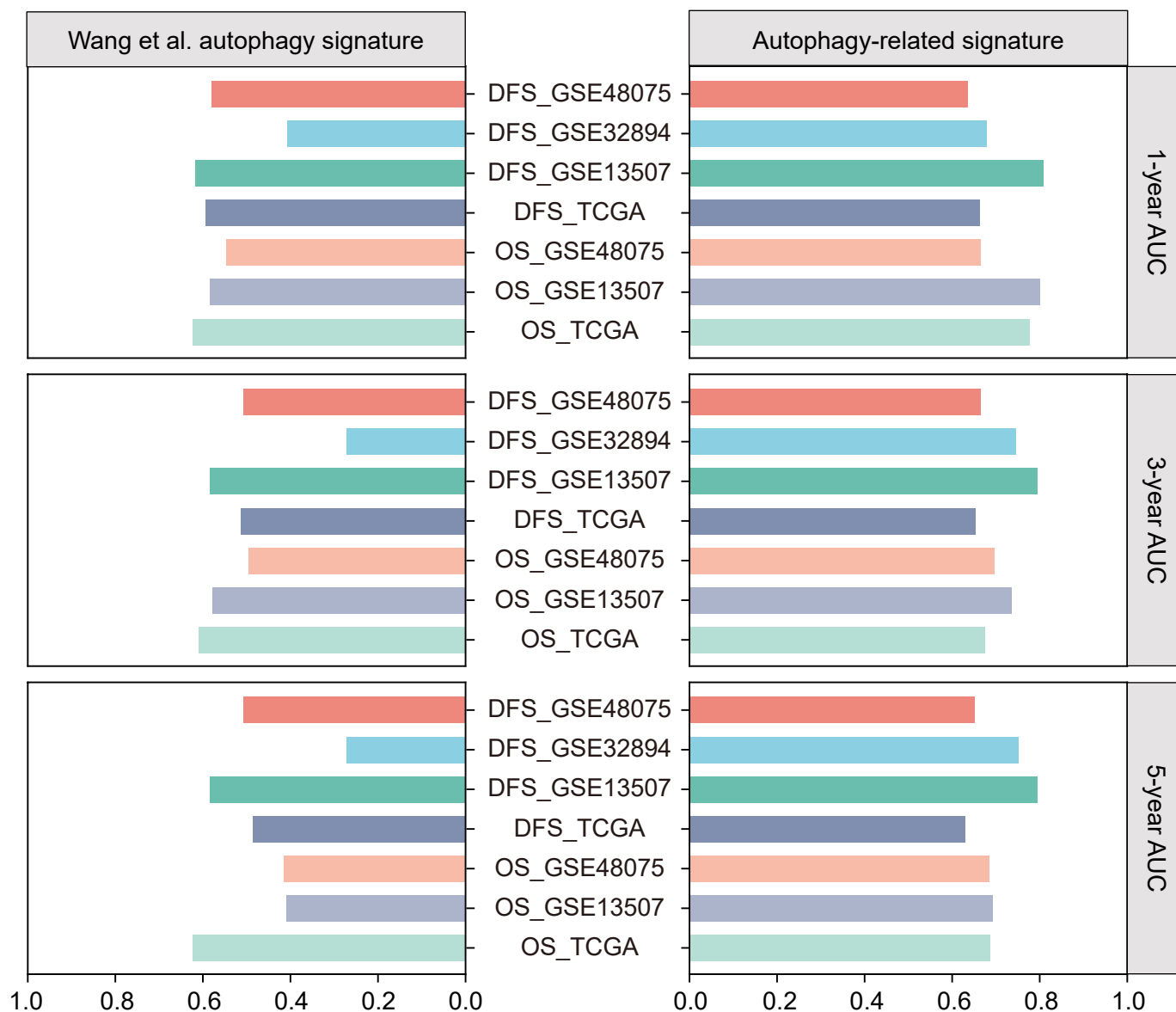

Supplement: Supplementary file 3 — Fig S3 [file JCMM-25-5417-s017.pdf]

**A**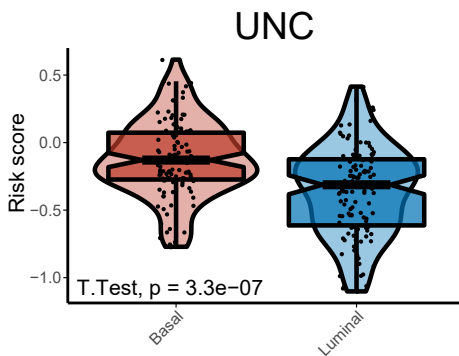**B**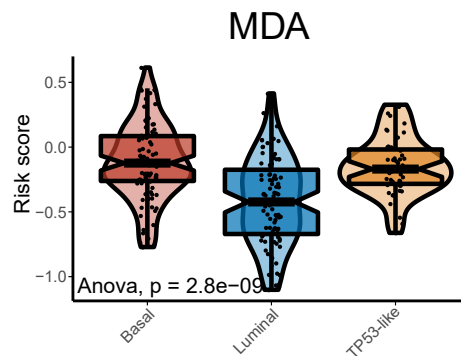**C**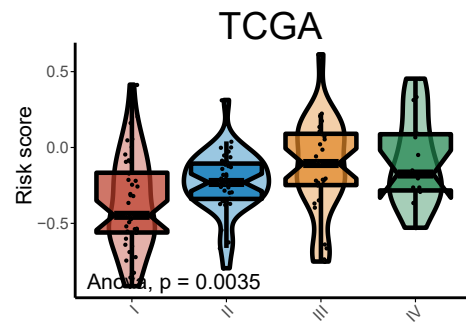**D**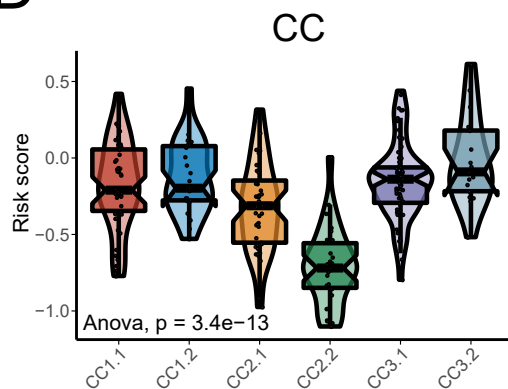**E**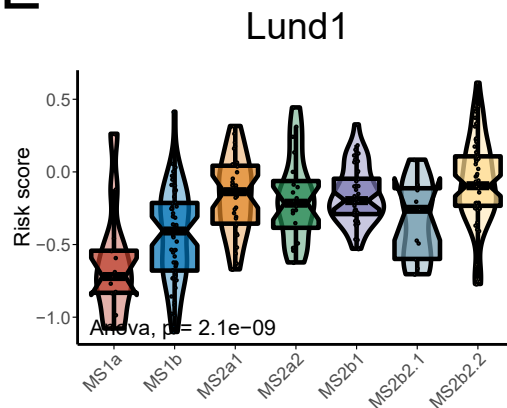**F**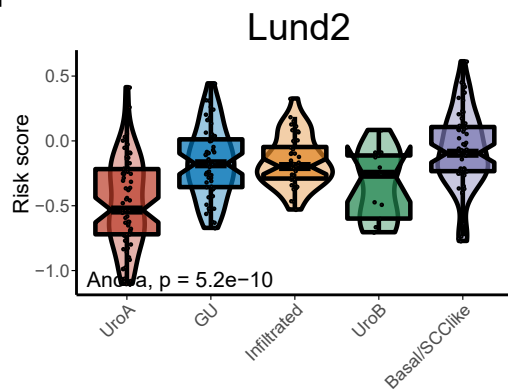**G**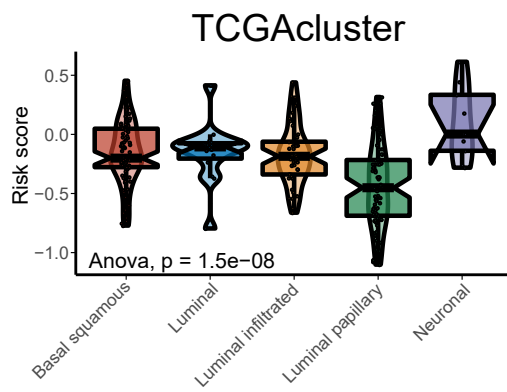

Supplement: Supplementary file 4 — Fig S4 [file JCMM-25-5417-s006.pdf]

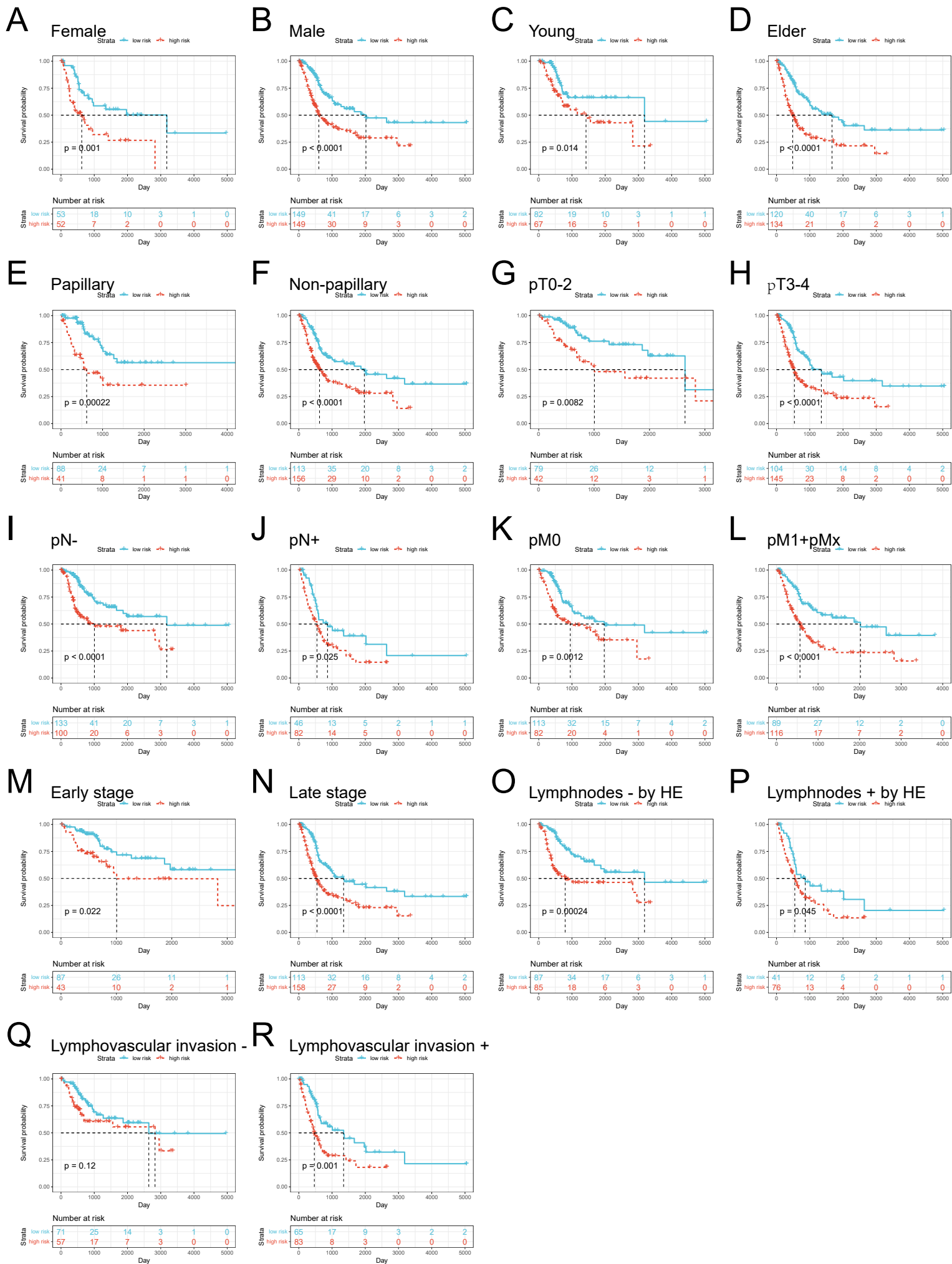

Supplement: Supplementary file 5 — Fig S5 [file JCMM-25-5417-s001.pdf]

Group High Low

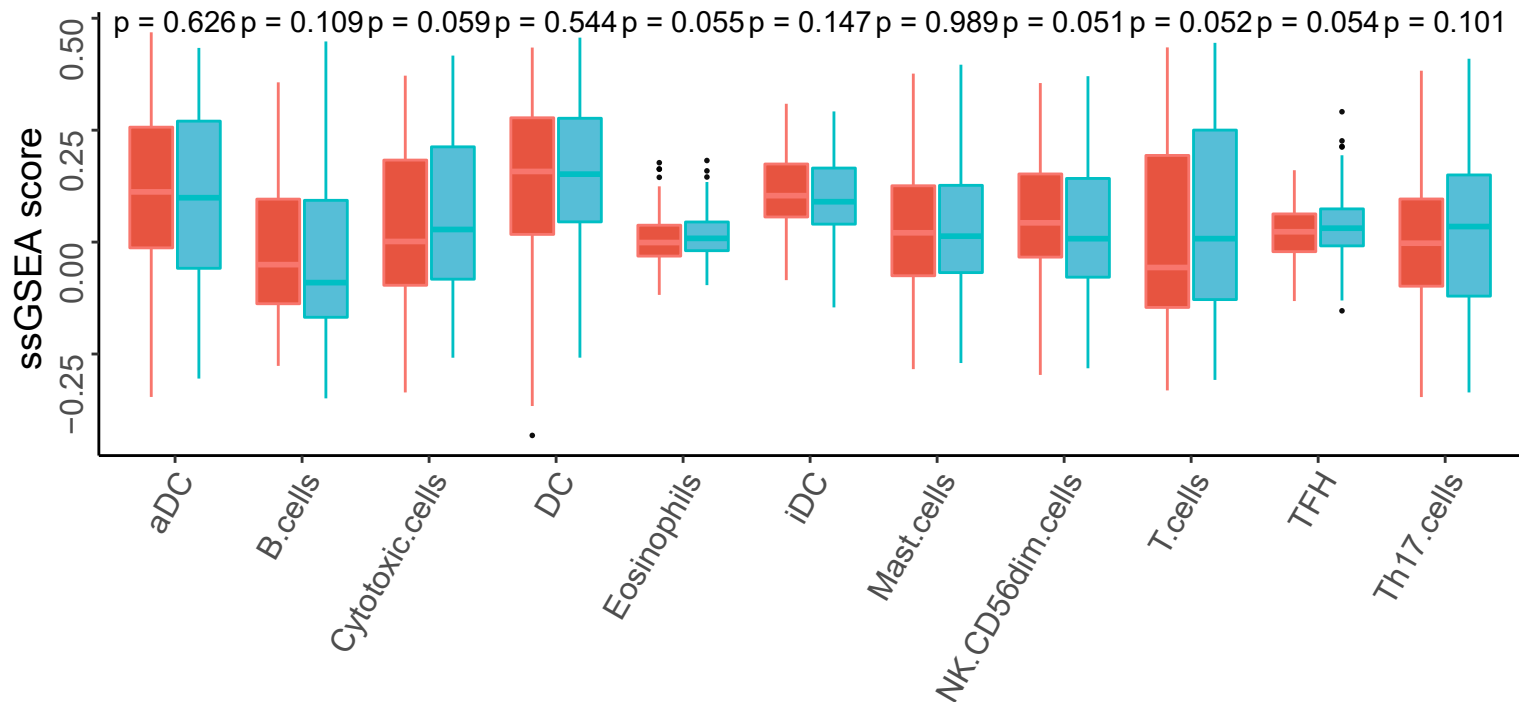

Supplement: Supplementary file 6 — Fig S6 [file JCMM-25-5417-s009.pdf]

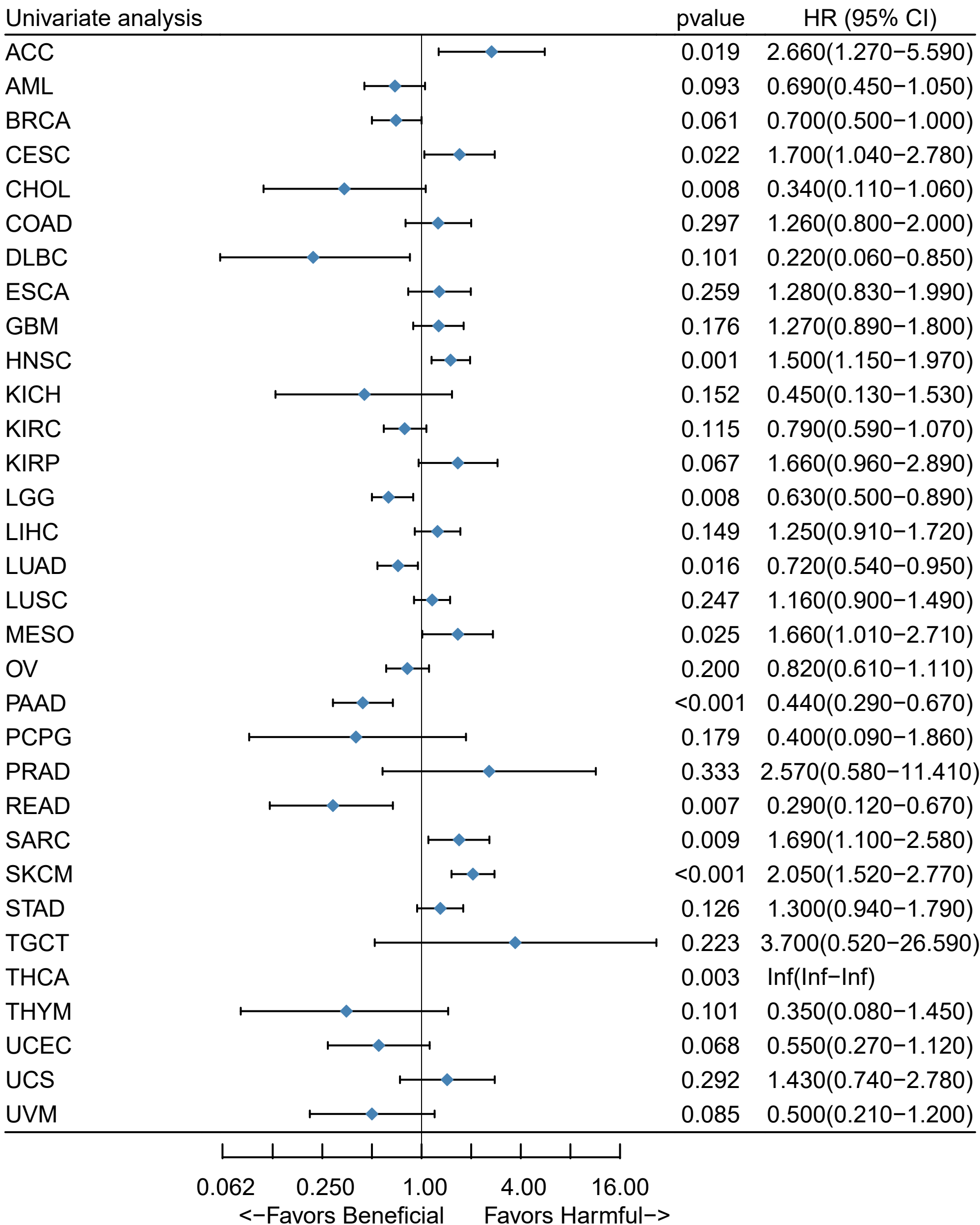

Supplement: Supplementary file 7 — Fig S7 [file JCMM-25-5417-s020.pdf]

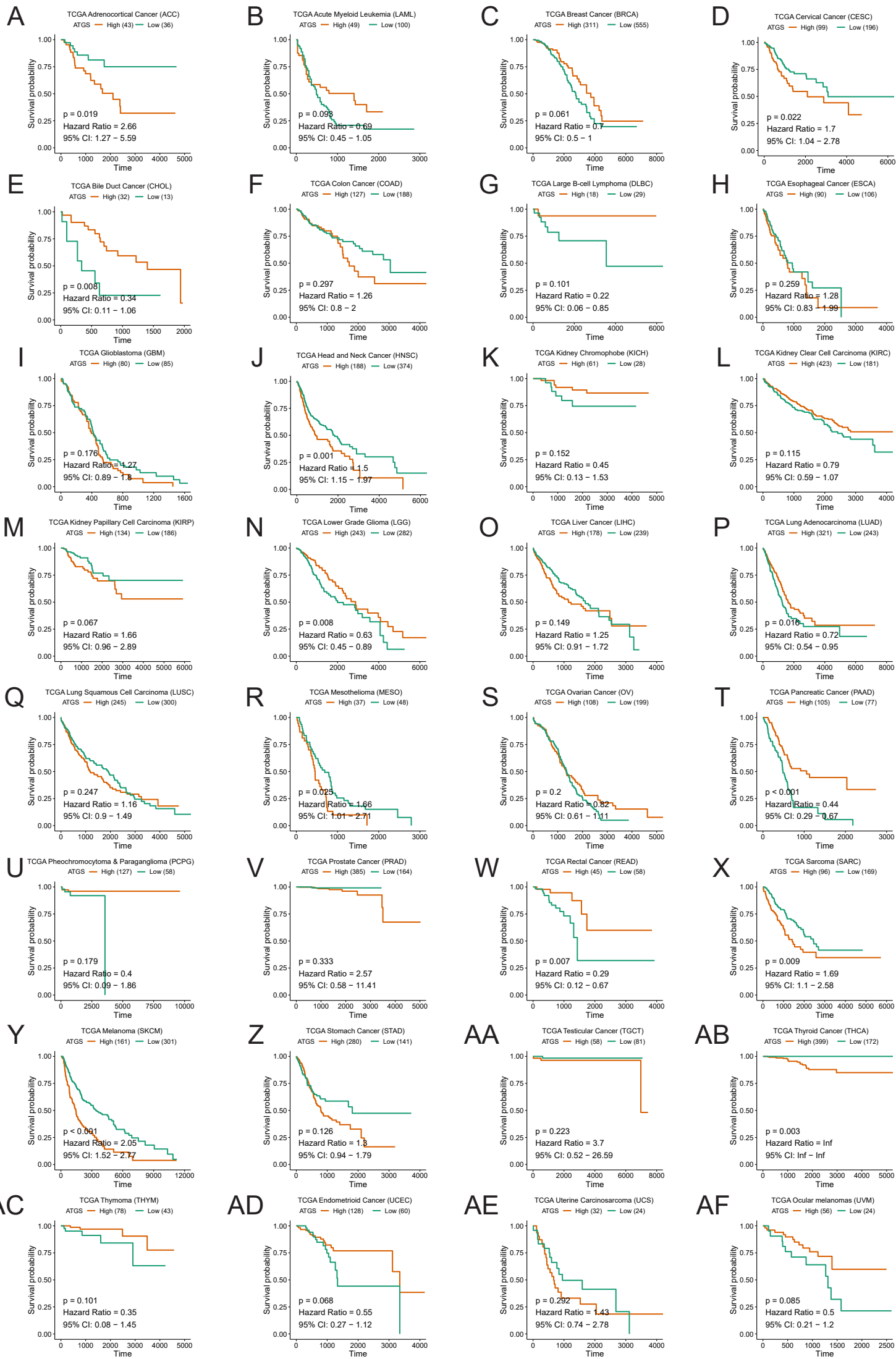

Supplement: Supplementary file 8 — Fig S8 [file JCMM-25-5417-s003.pdf]

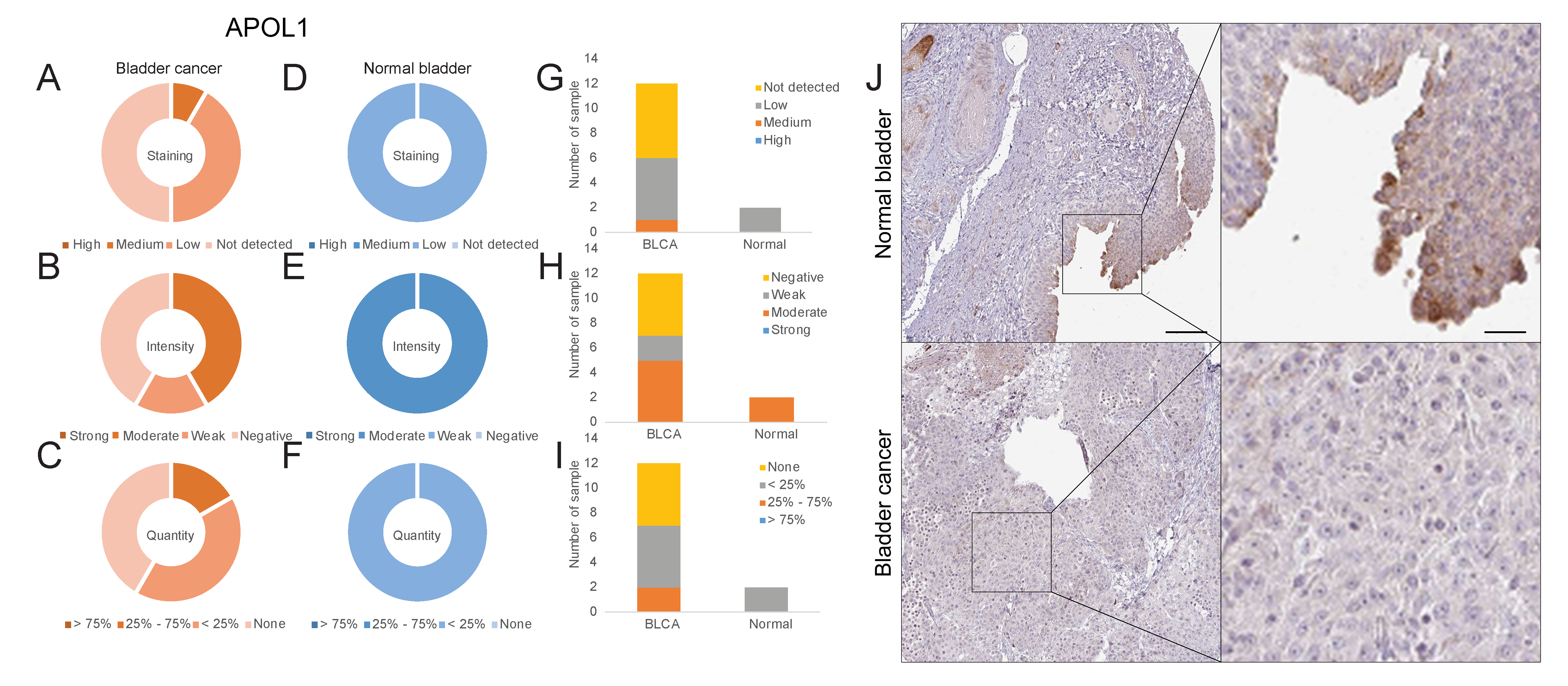

Supplement: Supplementary file 9 — Fig S9 [file JCMM-25-5417-s018.tif]

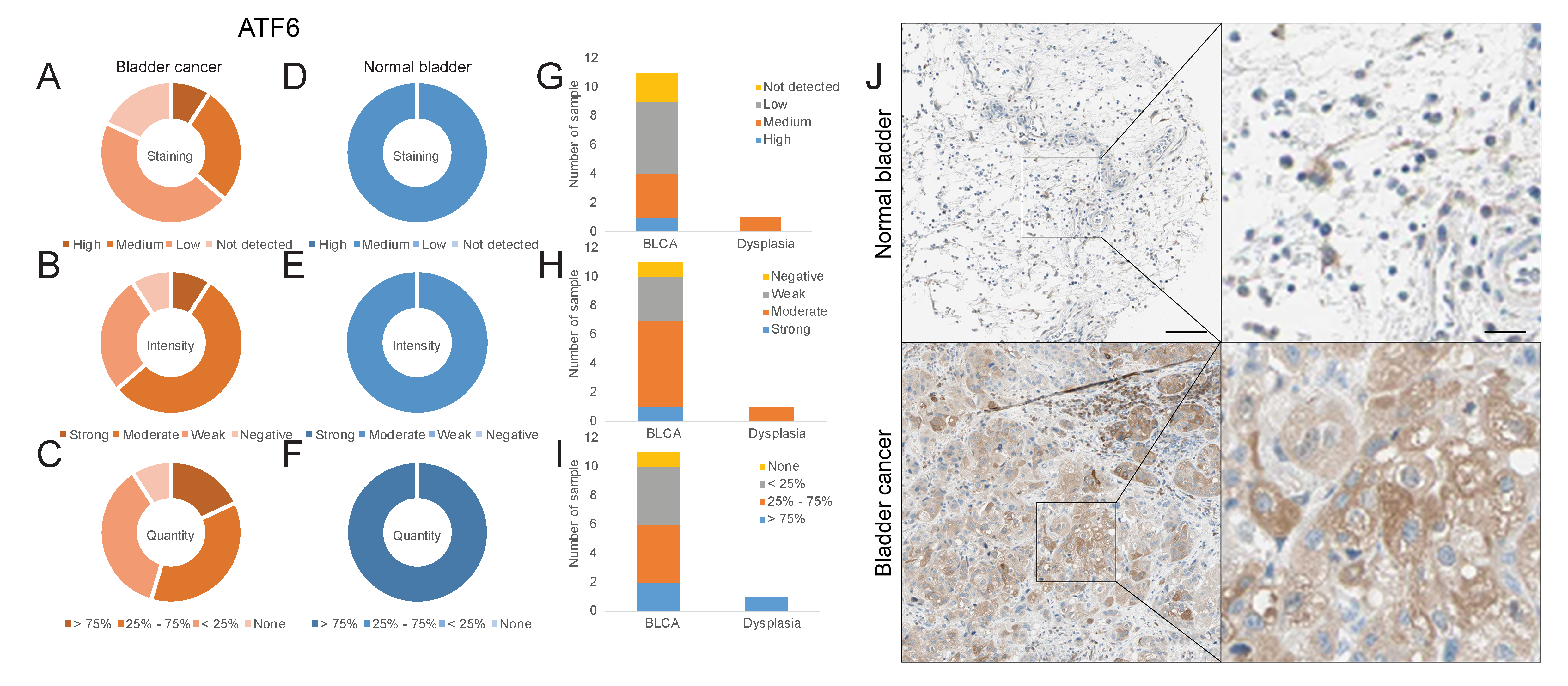

Supplement: Supplementary file 10 — Fig S10 [file JCMM-25-5417-s004.tif]

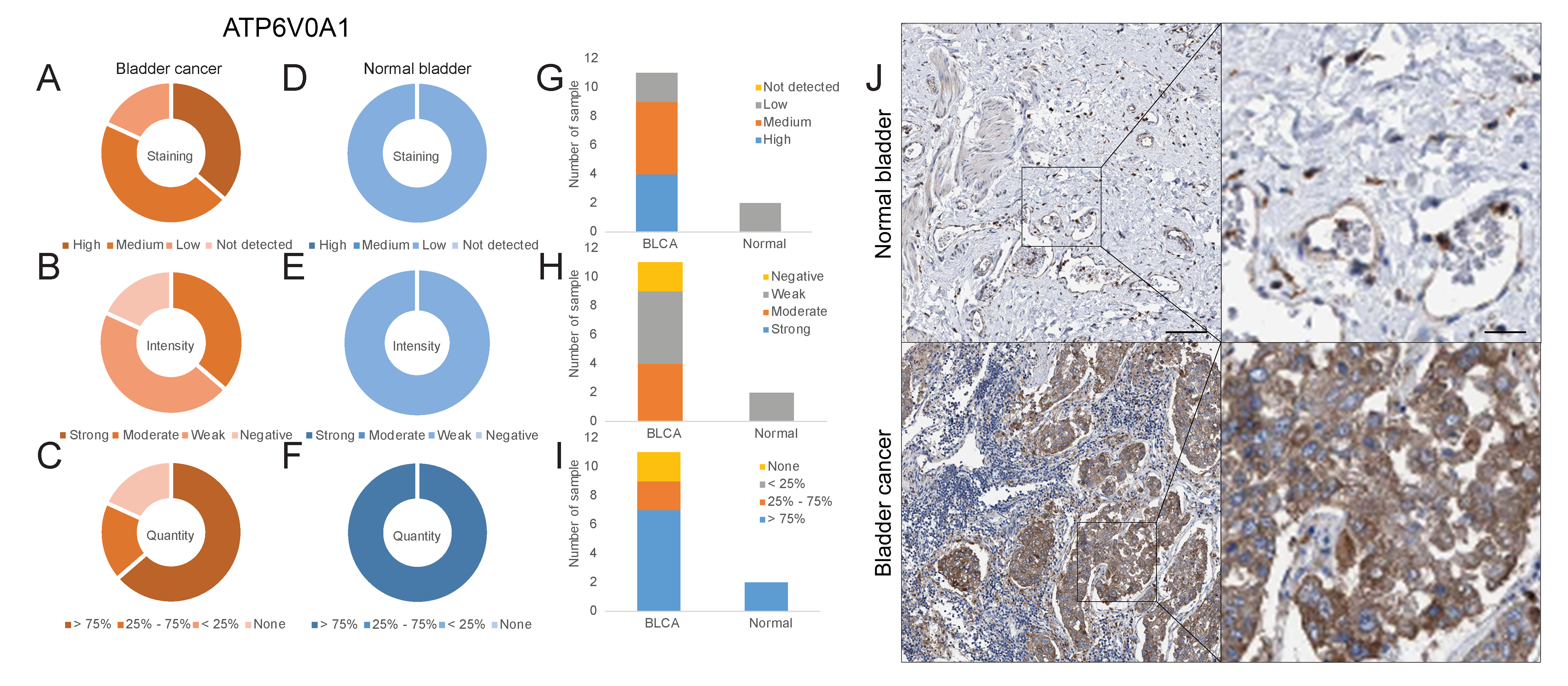

Supplement: Supplementary file 11 — Fig S11 [file JCMM-25-5417-s014.tif]

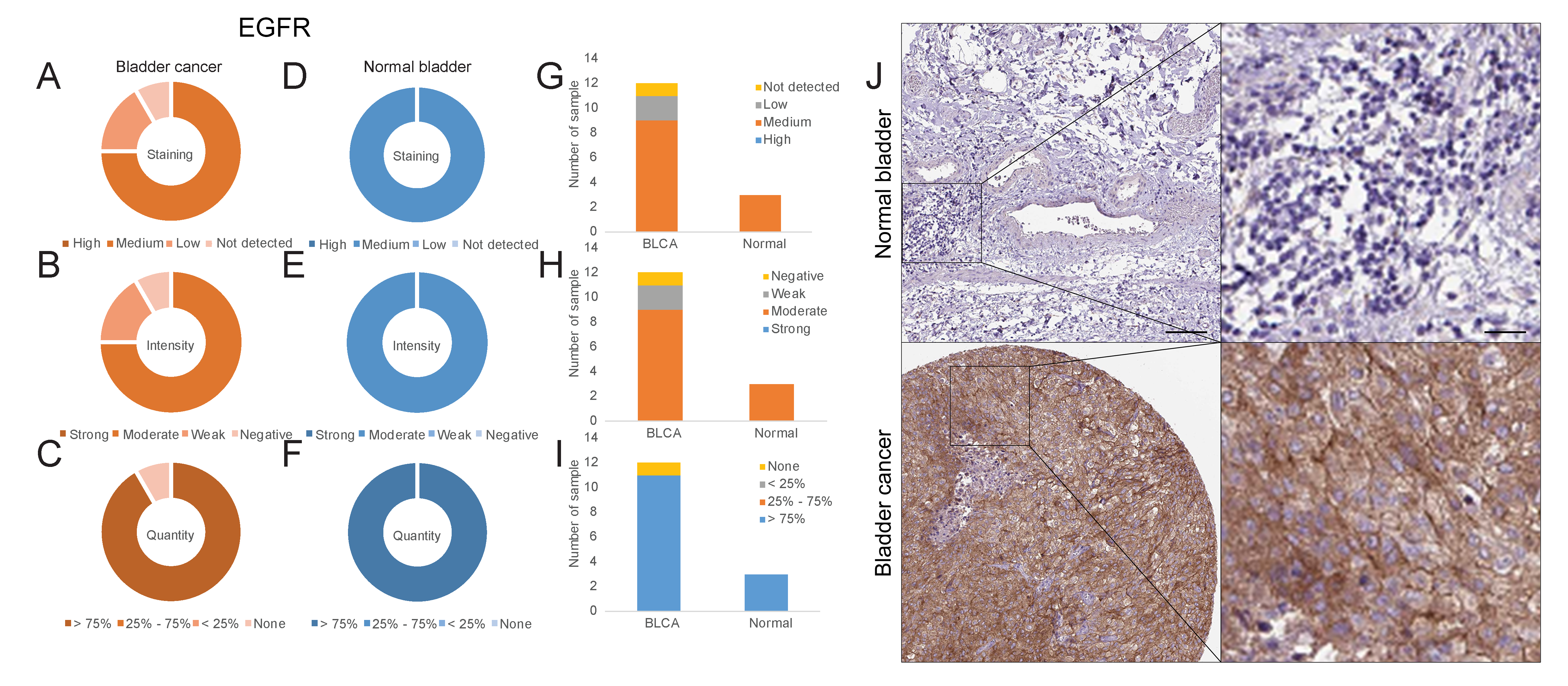

Supplement: Supplementary file 12 — Fig S12 [file JCMM-25-5417-s010.tif]

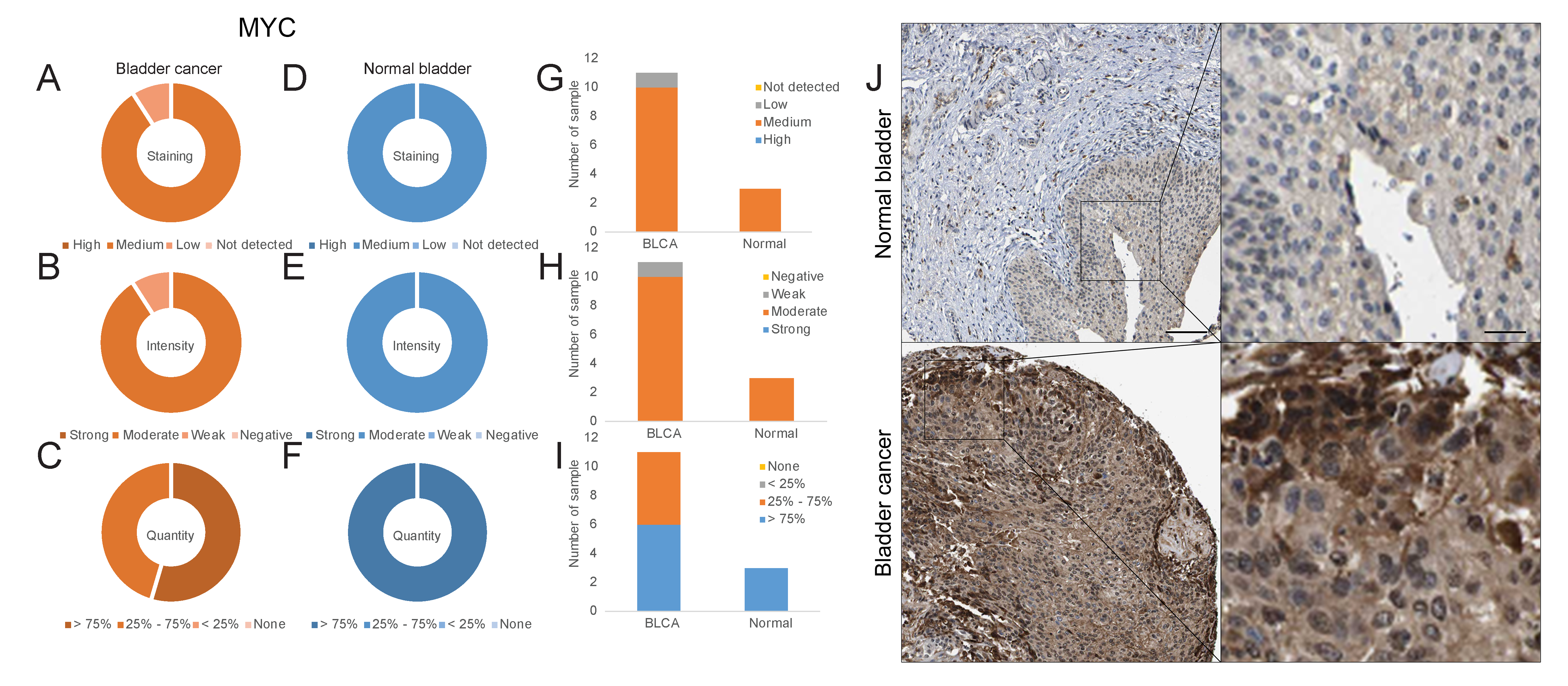

Supplement: Supplementary file 13 — Fig S13 [file JCMM-25-5417-s002.tif]

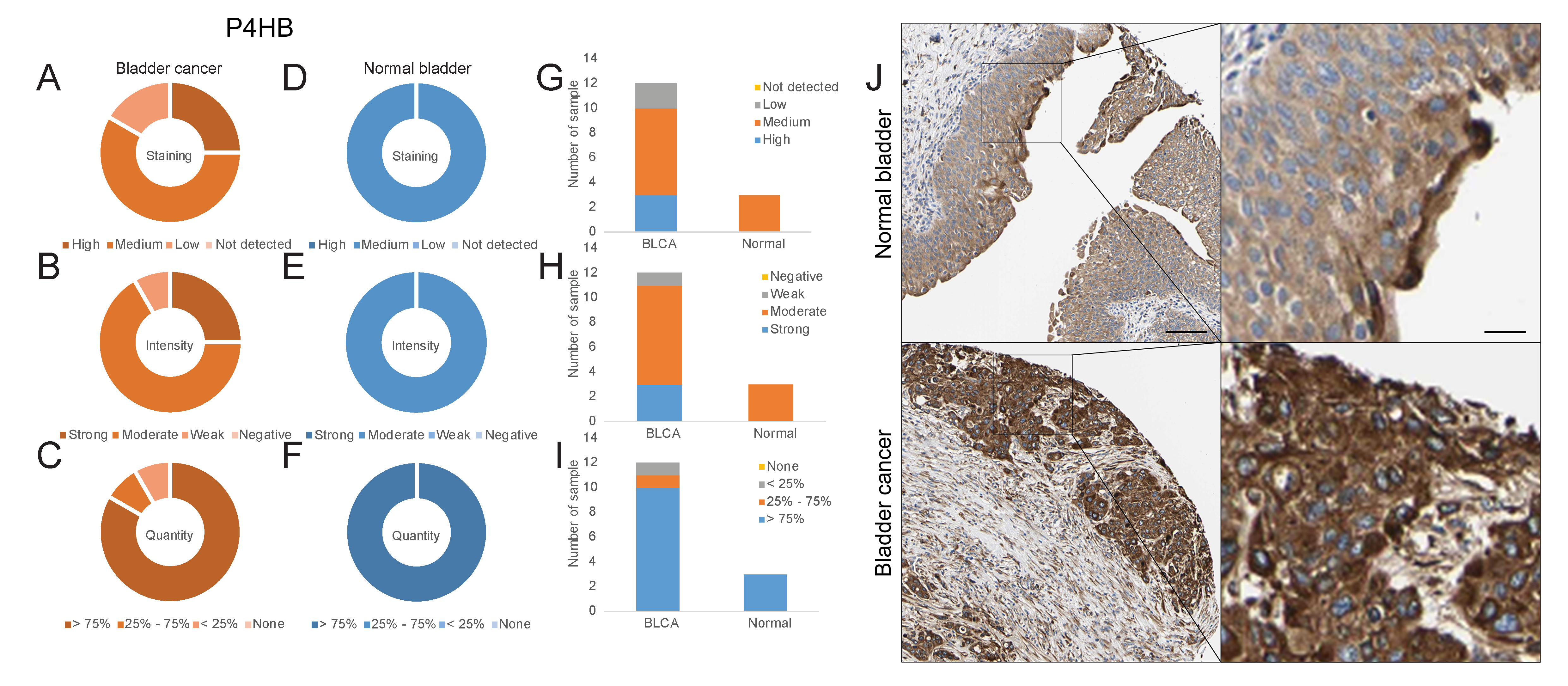

Supplement: Supplementary file 14 — Fig S14 [file JCMM-25-5417-s008.tif]

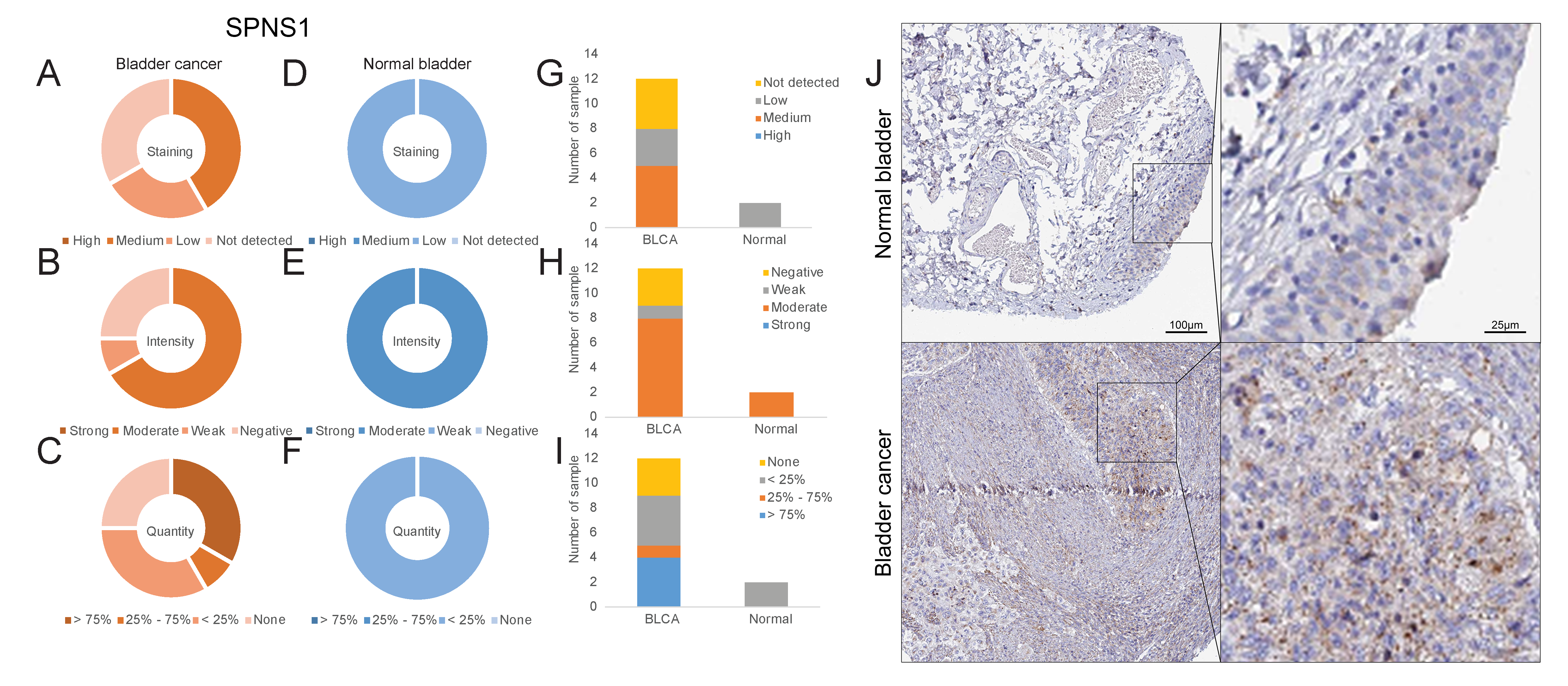

Supplement: Supplementary file 15 — Fig S15 [file JCMM-25-5417-s016.tif]

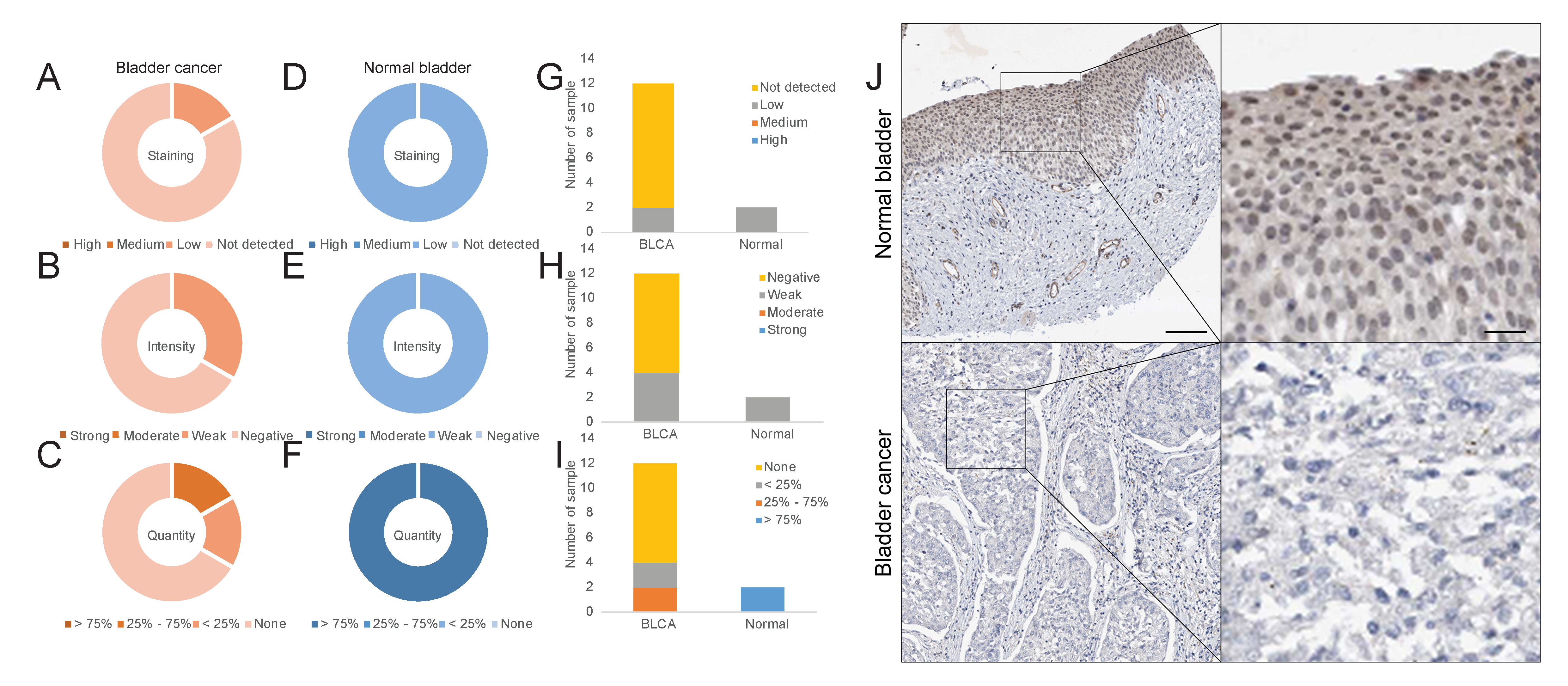

Supplement: Supplementary file 16 — Fig S16 [file JCMM-25-5417-s022.tif]

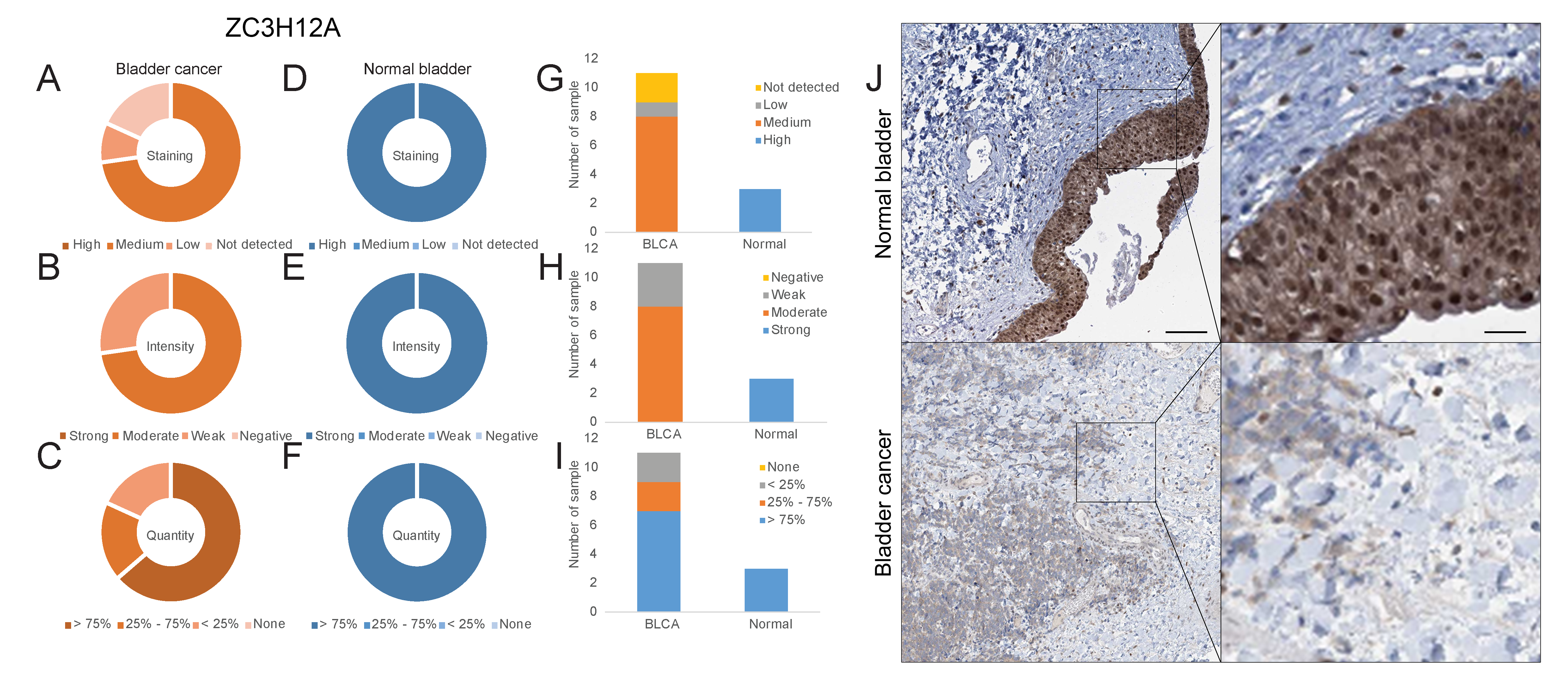

Supplement: Supplementary file 17 — Fig S17 [file JCMM-25-5417-s005.tif]
