# Supplementary material for: Identification of autophagy‐related genes signature predicts chemotherapeutic and immunotherapeutic efficiency in bladder cancer (BLCA)
Source: J Cell Mol Med. 2021 May 7;25(12):5417–33. doi: 10.1111/jcmm.16552 (PMC8184684; doi:10.1111/jcmm.16552)
Supplement: Supplementary file 18 — Table S1 [file JCMM-25-5417-s012.docx]

Additional file 1:Table S1 Brief information of GEO datasets in the study

| GEO number | Platform | Sample | | | |
| --- | --- | --- | --- | --- | --- |
|  |  | Tumor | Tumor with prognosis information | | Non-tumor |
| GSE13507 | GPL6102 Illumina human-6 v2.0 expression beadchip | 165 | 165 | 68 | |
| GSE32894 | GPL6947 Illumina HumanHT-12 V3.0 expression beadchip | 308 | 224 | - | |
| GSE48075 | GPL6947 Illumina HumanHT-12 V3.0 expression beadchip | 142 | 73 | - | |
