# Supplementary material for: Identification of autophagy‐related genes signature predicts chemotherapeutic and immunotherapeutic efficiency in bladder cancer (BLCA)
Source: J Cell Mol Med. 2021 May 7;25(12):5417–33. doi: 10.1111/jcmm.16552 (PMC8184684; doi:10.1111/jcmm.16552)
Supplement: Supplementary file 19 — Table S2 [file JCMM-25-5417-s011.docx]

**Additional file 2: Table S2. Summary of detailed clinical information of TCGA-BLCA cohort.**

| **TCGA-BLCA** | **Low(n=202)** | **High(n=201)** | **Total(n=403)** |
| --- | --- | --- | --- |
| **Age*** |  |  |  |
| <=65 | 92 (45.5%) | 67 (33.3%) | 159 (39.5%) |
| >65 | 110 (54.5%) | 134 (66.7%) | 244 (60.5%) |
| **Gender** |  |  |  |
| FEMALE | 49 (24.3%) | 56 (27.9%) | 105 (26.1%) |
| MALE | 153 (75.7%) | 145 (72.1%) | 298 (73.9%) |
| **Vital status***** |  |  |  |
| Alive | 143 (70.8%) | 83 (41.3%) | 226 (56.1%) |
| Dead | 59 (29.2%) | 118 (58.7%) | 177 (43.9%) |
| **Primary therapy outcome***** |  |  |  |
| CR | 143 (70.8%) | 82 (40.8%) | 225 (55.8%) |
| PR | 9 (4.5%) | 12 (6.0%) | 21 (5.2%) |
| SD | 9 (4.5%) | 20 (10.0%) | 29 (7.2%) |
| PD | 24 (11.9%) | 45 (22.4%) | 69 (17.1%) |
| **Additional treatment outcome***** |  |  |  |
| CR | 114 (56.4%) | 60 (29.9%) | 174 (43.2%) |
| PR | 1 (0.5%) | 5 (2.5%) | 6 (1.5%) |
| SD | 5 (2.5%) | 9 (4.5%) | 14 (3.5%) |
| PD | 40 (19.8%) | 58 (28.9%) | 98 (24.3%) |
| **Subtype***** |  |  |  |
| Non-Papillary | 111 (55.0%) | 158 (78.6%) | 269 (66.7%) |
| Papillary | 89 (44.1%) | 40 (19.9%) | 129 (32.0%) |
| **Lymphnodes positive by he**** |  |  |  |
| >0 | 42 (20.8%) | 75 (37.3%) | 117 (29.0%) |
| 0 | 91 (45.0%) | 81 (40.3%) | 172 (42.7%) |
| **Lymphovascular invasion**** |  |  |  |
| NO | 75 (37.1%) | 53 (26.4%) | 128 (31.8%) |
| YES | 58 (28.7%) | 90 (44.8%) | 148 (36.7%) |
| **AJCC pathologic T stage***** |  |  |  |
| T1 | 0(0%) | 1 (0.5%) | 1 (0.2%) |
| T2 | 76 (37.6%) | 44 (21.9%) | 120 (29.8%) |
| T3 | 86 (42.6%) | 105 (52.2%) | 191 (47.4%) |
| T4 | 20 (9.9%) | 38 (18.9%) | 58 (14.4%) |
| **AJCC pathologic N stage***** |  |  |  |
| N0 | 136 (67.3%) | 96 (47.8%) | 232 (57.6%) |
| N1 | 17 (8.4%) | 29 (14.4%) | 46 (11.4%) |
| N2 | 27 (13.4%) | 48 (23.9%) | 75 (18.6%) |
| N3 | 2 (1.0%) | 6 (3.0%) | 8 (2.0%) |
| **AJCC pathologic M stage** |  |  |  |
| M0 | 113 (55.9%) | 82 (40.8%) | 195 (48.4%) |
| M1 | 4 (2.0%) | 7 (3.5%) | 11 (2.7%) |
| **AJCC pathologic tumor stage***** |  |  |  |
| I | 0(0%) | 1 (0.5%) | 1 (0.2%) |
| II | 86 (42.6%) | 43 (21.4%) | 129 (32.0%) |
| III | 68 (33.7%) | 70 (34.8%) | 138 (34.2%) |
| IV | 46 (22.8%) | 87 (43.3%) | 133 (33.0%) |
| **Grade***** |  |  |  |
| High Grade | 181 (89.6%) | 199 (99.0%) | 380 (94.3%) |
| Low Grade | 20 (9.9%) | 0(0%) | 20 (5.0%) |
| **Tumor Status***** |  |  |  |
| TUMOR FREE | 125 (61.9%) | 77 (38.3%) | 202 (50.1%) |
| WITH TUMOR | 60 (29.7%) | 99 (49.3%) | 159 (39.5%) |
| **TCGA cluster***** |  |  |  |
| Basal squamous | 29 (14.4%) | 54 (26.9%) | 83 (20.6%) |
| Luminal | 4 (2.0%) | 11 (5.5%) | 15 (3.7%) |
| Luminal infiltrated | 19 (9.4%) | 26 (12.9%) | 45 (11.2%) |
| Luminal papillary | 59 (29.2%) | 21 (10.4%) | 80 (19.9%) |
| Neuronal | 2 (1.0%) | 7 (3.5%) | 9 (2.2%) |
| **Nature 2014 TCGA131*** |  |  |  |
| Cluster I | 27 (13.4%) | 14 (7.0%) | 41 (10.2%) |
| Cluster II | 20 (9.9%) | 21 (10.4%) | 41 (10.2%) |
| Cluster III | 9 (4.5%) | 20 (10.0%) | 29 (7.2%) |
| Cluster IV | 6 (3.0%) | 8 (4.0%) | 14 (3.5%) |
| **Scientific Reports 2015 CC***** |  |  |  |
| CC1.1 | 20 (9.9%) | 33 (16.4%) | 53 (13.2%) |
| CC1.2 | 9 (4.5%) | 14 (7.0%) | 23 (5.7%) |
| CC2.1 | 27 (13.4%) | 13 (6.5%) | 40 (9.9%) |
| CC2.2 | 29 (14.4%) | 1 (0.5%) | 30 (7.4%) |
| CC3.1 | 24 (11.9%) | 45 (22.4%) | 69 (17.1%) |
| CC3.2 | 4 (2.0%) | 13 (6.5%) | 17 (4.2%) |
| **Scientific Reports 2015 UNC***** |  |  |  |
| Basal | 37 (18.3%) | 73 (36.3%) | 110 (27.3%) |
| Luminal | 76 (37.6%) | 46 (22.9%) | 122 (30.3%) |
| **Scientific Reports 2015 MDA***** |  |  |  |
| Basal | 30 (14.9%) | 61 (30.3%) | 91 (22.6%) |
| Luminal | 65 (32.2%) | 28 (13.9%) | 93 (23.1%) |
| TP53 like | 18 (8.9%) | 30 (14.9%) | 48 (11.9%) |
| **Lund1***** |  |  |  |
| MS1a | 11 (5.4%) | 2 (1.0%) | 13 (3.2%) |
| MS1b | 40 (19.8%) | 16 (8.0%) | 56 (13.9%) |
| MS2a1 | 14 (6.9%) | 18 (9.0%) | 32 (7.9%) |
| MS2a2 | 10 (5.0%) | 10 (5.0%) | 20 (5.0%) |
| MS2b1 | 16 (7.9%) | 25 (12.4%) | 41 (10.2%) |
| MS2b2.1 | 7 (3.5%) | 6 (3.0%) | 13 (3.2%) |
| MS2b2.2 | 15 (7.4%) | 42 (20.9%) | 57 (14.1%) |
| **Lund2***** |  |  |  |
| Basal/SCClike | 15 (7.4%) | 42 (20.9%) | 57 (14.1%) |
| Genomically unstable | 24 (11.9%) | 28 (13.9%) | 52 (12.9%) |
| Infiltrated | 16 (7.9%) | 25 (12.4%) | 41 (10.2%) |
| UroA | 51 (25.2%) | 18 (9.0%) | 69 (17.1%) |
| UroB | 7 (3.5%) | 6 (3.0%) | 13 (3.2%) |
| **Mutation in TP53***** |  |  |  |
| no | 126 (62.4%) | 82 (40.8%) | 208 (51.6%) |
| yes | 76 (37.6%) | 119 (59.2%) | 195 (48.4%) |
| **Mutation in FGFR3***** |  |  |  |
| no | 159 (78.7%) | 188 (93.5%) | 347 (86.1%) |
| yes | 43 (21.3%) | 13 (6.5%) | 56 (13.9%) |
| **Mutation in RB1*** |  |  |  |
| no | 175 (86.6%) | 158 (78.6%) | 333 (82.6%) |
| yes | 27 (13.4%) | 43 (21.4%) | 70 (17.4%) |

***χ*2 test, * p < 0.05, ** p < 0.01, *** p <0.001**
