# Supplementary material for: Identification of autophagy‐related genes signature predicts chemotherapeutic and immunotherapeutic efficiency in bladder cancer (BLCA)
Source: J Cell Mol Med. 2021 May 7;25(12):5417–33. doi: 10.1111/jcmm.16552 (PMC8184684; doi:10.1111/jcmm.16552)
Supplement: Supplementary file 21 — Table S4 [file JCMM-25-5417-s021.docx]

Additional file 10: Table S4. The detailed information of immunotherapy response based on TIDE algorithm in TCGA-BLCA cohort.

| Patient | No benefits | Responder | TIDE | IFNG | MSI Score | CTL.flag | Dysfunction | Exclusion | MDSC | CAF | TAM M2 |
| --- | --- | --- | --- | --- | --- | --- | --- | --- | --- | --- | --- |
| TCGA-BL-A5ZZ-01 | TRUE | FALSE | 2.17 | -157.73 | 0 | FALSE | 0.14 | 2.17 | 0.02 | 0.32 | -0.01 |
| TCGA-XF-A9SP-01 | FALSE | FALSE | 1.87 | 333.62 | 0 | FALSE | 0.25 | 1.87 | 0 | 0.28 | 0.01 |
| TCGA-K4-A3WS-01 | FALSE | FALSE | 1.87 | 2994.53 | 0 | FALSE | 0.24 | 1.87 | -0.01 | 0.3 | -0.01 |
| TCGA-SY-A9G0-01 | TRUE | FALSE | 1.85 | -1168.51 | 1 | FALSE | 0.16 | 1.85 | 0 | 0.27 | 0.02 |
| TCGA-FD-A5BZ-01 | TRUE | FALSE | 1.82 | -1119.27 | 0 | FALSE | 0.14 | 1.82 | 0.01 | 0.27 | 0 |
| TCGA-GD-A3OS-01 | TRUE | FALSE | 1.82 | -1582.07 | 0 | FALSE | 0.11 | 1.82 | 0.02 | 0.26 | 0 |
| TCGA-XF-AAMJ-01 | TRUE | FALSE | 1.82 | -1261.29 | 0 | FALSE | 0.13 | 1.82 | 0 | 0.27 | 0 |
| TCGA-FD-A6TF-01 | TRUE | FALSE | 1.81 | -1230.51 | 0 | FALSE | 0.13 | 1.81 | 0.05 | 0.21 | 0.01 |
| TCGA-DK-A3IQ-01 | TRUE | FALSE | 1.79 | -755.31 | 0 | FALSE | 0.12 | 1.79 | 0.01 | 0.27 | 0 |
| TCGA-XF-AAN7-01 | TRUE | FALSE | 1.79 | -1314.25 | 0 | FALSE | -0.18 | 1.79 | 0.06 | 0.21 | 0.01 |
| TCGA-UY-A8OC-01 | TRUE | FALSE | 1.77 | -1950.71 | 1 | FALSE | 0.09 | 1.77 | 0.06 | 0.21 | 0.01 |
| TCGA-XF-AAME-01 | FALSE | FALSE | 1.75 | 6661.14 | 0 | FALSE | 0.26 | 1.75 | -0.02 | 0.29 | 0 |
| TCGA-FD-A62S-01 | FALSE | FALSE | 1.73 | 7004.88 | 0 | FALSE | 0.2 | 1.73 | 0.01 | 0.27 | -0.01 |
| TCGA-GC-A3YS-01 | FALSE | FALSE | 1.72 | 2398.14 | 0 | FALSE | 0.04 | 1.72 | 0.02 | 0.26 | -0.01 |
| TCGA-ZF-A9R9-01 | TRUE | FALSE | 1.7 | -1227.99 | 0 | FALSE | 0.16 | 1.7 | 0 | 0.24 | 0.02 |
| TCGA-BT-A20R-01 | TRUE | FALSE | 1.67 | -79.6 | 0 | FALSE | 0.06 | 1.67 | 0 | 0.26 | 0 |
| TCGA-FJ-A871-01 | TRUE | FALSE | 1.67 | -3132.31 | nan | FALSE | 0.04 | 1.67 | 0.07 | 0.17 | 0.01 |
| TCGA-FD-A3SQ-01 | FALSE | FALSE | 1.62 | 3748.4 | 0 | FALSE | 0.3 | 1.62 | -0.03 | 0.29 | -0.01 |
| TCGA-G2-A2EC-01 | TRUE | FALSE | 1.61 | -978.96 | 0 | FALSE | 0.21 | 1.61 | -0.02 | 0.23 | 0.03 |
| TCGA-ZF-AA52-01 | FALSE | FALSE | 1.61 | 1041.8 | 0 | FALSE | 0.22 | 1.61 | 0 | 0.25 | 0 |
| TCGA-XF-AAMT-01 | FALSE | FALSE | 1.6 | 1295.23 | 0 | FALSE | 0.23 | 1.6 | 0.02 | 0.24 | -0.01 |
| TCGA-DK-AA6S-01 | FALSE | FALSE | 1.6 | 5897.69 | 0 | FALSE | 0.26 | 1.6 | 0 | 0.25 | -0.01 |
| TCGA-XF-A9SW-01 | FALSE | FALSE | 1.6 | 2857.35 | 1 | FALSE | 0.15 | 1.6 | 0 | 0.24 | 0 |
| TCGA-ZF-AA4R-01 | FALSE | FALSE | 1.53 | 817.96 | 0 | FALSE | 0.01 | 1.53 | 0 | 0.24 | -0.01 |
| TCGA-FD-A3SN-01 | FALSE | FALSE | 1.52 | 1467.14 | 0 | FALSE | 0.16 | 1.52 | 0.06 | 0.17 | 0 |
| TCGA-FD-A6TC-01 | TRUE | FALSE | 1.51 | -1718.99 | 0 | FALSE | 0.14 | 1.51 | 0.02 | 0.23 | -0.02 |
| TCGA-XF-A9T3-01 | FALSE | FALSE | 1.5 | 5940.36 | 0 | FALSE | 0.46 | 1.5 | 0.03 | 0.23 | -0.03 |
| TCGA-DK-A3IN-01 | FALSE | FALSE | 1.47 | 3545.84 | 0 | FALSE | 0.12 | 1.47 | 0.03 | 0.2 | 0 |
| TCGA-XF-A9SL-01 | FALSE | FALSE | 1.46 | 6314.71 | nan | FALSE | 0.33 | 1.46 | -0.01 | 0.22 | 0.01 |
| TCGA-FD-A3SL-01 | FALSE | FALSE | 1.44 | 4832.15 | 0 | FALSE | 0.2 | 1.44 | -0.02 | 0.24 | 0 |
| TCGA-ZF-A9RC-01 | TRUE | FALSE | 1.43 | -2858.59 | 0 | FALSE | 0.13 | 1.43 | 0.02 | 0.19 | 0.01 |
| TCGA-DK-A1A5-01 | FALSE | FALSE | 1.41 | 7642.44 | 0 | FALSE | 0.04 | 1.41 | 0.01 | 0.23 | -0.02 |
| TCGA-XF-A9SZ-01 | FALSE | FALSE | 1.38 | 3755.86 | 0 | FALSE | 0.17 | 1.38 | 0.01 | 0.23 | -0.02 |
| TCGA-DK-A2I1-01 | FALSE | FALSE | 1.38 | 3751.25 | 0 | FALSE | -0.08 | 1.38 | 0.03 | 0.16 | 0.02 |
| TCGA-DK-A1AB-01 | FALSE | FALSE | 1.36 | 14445.89 | 0 | FALSE | 0.15 | 1.36 | 0.01 | 0.22 | -0.02 |
| TCGA-XF-A9SK-01 | FALSE | FALSE | 1.35 | 4355.38 | 0 | FALSE | 0.35 | 1.35 | -0.01 | 0.25 | -0.02 |
| TCGA-DK-A2HX-01 | FALSE | FALSE | 1.33 | 30.59 | 0 | FALSE | 0.23 | 1.33 | 0 | 0.21 | -0.01 |
| TCGA-XF-A9SV-01 | TRUE | FALSE | 1.33 | -2024.52 | 0 | FALSE | -0.01 | 1.33 | 0.04 | 0.16 | 0 |
| TCGA-K4-A4AB-01 | TRUE | FALSE | 1.3 | -404.99 | 0 | FALSE | 0.36 | 1.3 | 0.01 | 0.21 | -0.01 |
| TCGA-UY-A78M-01 | TRUE | FALSE | 1.29 | -2966.31 | 1 | FALSE | -0.12 | 1.29 | 0.06 | 0.11 | 0.03 |
| TCGA-XF-A9T2-01 | TRUE | FALSE | 1.28 | -2608.41 | nan | FALSE | 0.12 | 1.28 | 0.06 | 0.13 | 0.01 |
| TCGA-KQ-A41S-01 | FALSE | FALSE | 1.23 | 2029.99 | 0 | FALSE | 0.09 | 1.23 | -0.01 | 0.22 | -0.01 |
| TCGA-FD-A6TG-01 | FALSE | FALSE | 1.22 | 1942.93 | 0 | FALSE | 0.37 | 1.22 | -0.02 | 0.2 | 0.01 |
| TCGA-XF-A9T4-01 | FALSE | FALSE | 1.19 | 8331.65 | 0 | FALSE | 0.2 | 1.19 | 0.07 | 0.15 | -0.03 |
| TCGA-GU-AATP-01 | FALSE | FALSE | 1.18 | 853.03 | 0 | FALSE | 0.28 | 1.18 | 0 | 0.2 | -0.02 |
| TCGA-ZF-AA54-01 | FALSE | FALSE | 1.03 | 7400.82 | 0 | FALSE | 0.05 | 1.03 | 0.02 | 0.16 | -0.02 |
| TCGA-YC-A8S6-01 | FALSE | FALSE | 1.01 | 574.84 | 0 | FALSE | 0.52 | 1.01 | 0.02 | 0.15 | -0.01 |
| TCGA-FD-A6TA-01 | FALSE | FALSE | 0.99 | 3350.67 | 0 | FALSE | 0.23 | 0.99 | 0 | 0.17 | -0.01 |
| TCGA-FD-A3SO-01 | FALSE | FALSE | 0.98 | 7518.51 | 1 | FALSE | 0.01 | 0.98 | 0.01 | 0.17 | -0.02 |
| TCGA-BL-A13J-01 | FALSE | FALSE | 0.98 | 354.73 | nan | FALSE | -0.25 | 0.98 | 0.02 | 0.15 | -0.02 |
| TCGA-GV-A40E-01 | FALSE | FALSE | 0.98 | 903 | 0 | FALSE | 0.05 | 0.98 | 0.06 | 0.13 | -0.04 |
| TCGA-ZF-AA5P-01 | FALSE | FALSE | 0.95 | 266.22 | 0 | FALSE | 0.16 | 0.95 | 0 | 0.15 | 0 |
| TCGA-BT-A3PK-01 | FALSE | FALSE | 0.92 | 2489.33 | 0 | FALSE | 0.09 | 0.92 | 0.02 | 0.16 | -0.05 |
| TCGA-DK-A2I6-01 | TRUE | FALSE | 0.92 | -449.13 | 1 | FALSE | -0.28 | 0.92 | 0.08 | 0.04 | 0.02 |
| TCGA-4Z-AA82-01 | FALSE | FALSE | 0.91 | 1778.98 | 0 | FALSE | 0.09 | 0.91 | 0.02 | 0.14 | -0.02 |
| TCGA-FD-A6TD-01 | FALSE | FALSE | 0.9 | 8007.12 | 0 | FALSE | 0.21 | 0.9 | 0.03 | 0.14 | -0.04 |
| TCGA-HQ-A5NE-01 | FALSE | FALSE | 0.9 | 19.81 | 0 | FALSE | 0.21 | 0.9 | 0.03 | 0.16 | -0.05 |
| TCGA-XF-AAMR-01 | FALSE | FALSE | 0.89 | 1146.22 | 0 | FALSE | 0.19 | 0.89 | -0.03 | 0.15 | 0.01 |
| TCGA-FT-A3EE-01 | TRUE | FALSE | 0.88 | -2021.92 | nan | FALSE | -0.31 | 0.88 | 0.06 | 0.06 | 0.01 |
| TCGA-XF-AAMW-01 | FALSE | FALSE | 0.88 | 4717.66 | 0 | FALSE | 0.13 | 0.88 | 0.05 | 0.1 | -0.02 |
| TCGA-C4-A0F6-01 | TRUE | FALSE | 0.87 | -948.9 | 0 | FALSE | -0.07 | 0.87 | 0.02 | 0.08 | 0.03 |
| TCGA-XF-AAMH-01 | TRUE | FALSE | 0.86 | -2531.17 | nan | FALSE | 0.06 | 0.86 | 0.06 | 0.04 | 0.02 |
| TCGA-FD-A5BY-01 | FALSE | FALSE | 0.86 | 1034.71 | 0 | FALSE | 0.14 | 0.86 | 0.01 | 0.13 | 0 |
| TCGA-K4-AAQO-01 | FALSE | FALSE | 0.86 | 910.94 | 1 | FALSE | 0.08 | 0.86 | 0 | 0.11 | 0.02 |
| TCGA-UY-A9PD-01 | TRUE | FALSE | 0.86 | -1332.68 | 0 | FALSE | -0.19 | 0.86 | 0.04 | 0.06 | 0.03 |
| TCGA-DK-A2I2-01 | FALSE | FALSE | 0.85 | 7001.97 | 0 | FALSE | -0.05 | 0.85 | 0.03 | 0.1 | -0.01 |
| TCGA-GD-A3OQ-01 | TRUE | FALSE | 0.85 | -1193.91 | nan | FALSE | 0.05 | 0.85 | 0.06 | 0.09 | -0.02 |
| TCGA-KQ-A41P-01 | TRUE | FALSE | 0.84 | -1110.5 | 1 | FALSE | 0.17 | 0.84 | 0.02 | 0.07 | 0.03 |
| TCGA-GC-A3OO-01 | TRUE | FALSE | 0.83 | -351.43 | 0 | FALSE | 0.21 | 0.83 | -0.02 | 0.15 | 0 |
| TCGA-BT-A20N-01 | TRUE | FALSE | 0.82 | -1565.12 | nan | FALSE | -0.47 | 0.82 | 0.07 | 0.04 | 0.02 |
| TCGA-GU-A764-01 | FALSE | FALSE | 0.82 | 2877.87 | 0 | FALSE | 0.13 | 0.82 | 0.01 | 0.13 | -0.02 |
| TCGA-FD-A62O-01 | TRUE | FALSE | 0.8 | -2536.36 | 0 | FALSE | -0.15 | 0.8 | 0.04 | 0.04 | 0.04 |
| TCGA-ZF-AA5N-01 | TRUE | FALSE | 0.77 | -1465.24 | nan | FALSE | -0.04 | 0.77 | 0.05 | 0.06 | 0 |
| TCGA-GU-A767-01 | TRUE | FALSE | 0.77 | -2304.98 | nan | FALSE | 0.04 | 0.77 | 0.06 | 0.01 | 0.03 |
| TCGA-XF-AAMY-01 | TRUE | FALSE | 0.76 | -1645.99 | 0 | FALSE | 0.06 | 0.76 | -0.01 | 0.12 | 0.01 |
| TCGA-BL-A13I-01 | FALSE | FALSE | 0.74 | 8635.08 | 0 | FALSE | 0.21 | 0.74 | -0.03 | 0.14 | 0.02 |
| TCGA-FD-A6TH-01 | TRUE | FALSE | 0.74 | -179.73 | 0 | FALSE | 0.1 | 0.74 | 0.03 | 0.1 | -0.01 |
| TCGA-XF-AAN3-01 | FALSE | FALSE | 0.73 | 2470 | 0 | FALSE | 0.06 | 0.73 | -0.01 | 0.13 | -0.01 |
| TCGA-K4-A6MB-01 | TRUE | FALSE | 0.72 | -2312.93 | 0 | FALSE | -0.03 | 0.72 | 0 | 0.11 | 0 |
| TCGA-DK-A3IT-01 | FALSE | FALSE | 0.71 | 562.99 | 1 | FALSE | 0.04 | 0.71 | 0 | 0.12 | 0 |
| TCGA-DK-AA6M-01 | FALSE | FALSE | 0.7 | 6098.75 | 0 | FALSE | 0.19 | 0.7 | 0.03 | 0.08 | 0 |
| TCGA-YC-A89H-01 | FALSE | FALSE | 0.69 | 136.2 | nan | FALSE | -0.03 | 0.69 | 0.04 | 0.03 | 0.03 |
| TCGA-FD-A3SM-01 | TRUE | FALSE | 0.68 | -1118.57 | 0 | FALSE | 0.08 | 0.68 | -0.02 | 0.15 | -0.03 |
| TCGA-XF-AAMX-01 | TRUE | FALSE | 0.68 | -1297.61 | 0 | FALSE | 0 | 0.68 | 0.01 | 0.07 | 0.02 |
| TCGA-UY-A8OD-01 | FALSE | FALSE | 0.67 | 585.63 | 0 | FALSE | 0.05 | 0.67 | 0.01 | 0.07 | 0.02 |
| TCGA-ZF-A9R7-01 | FALSE | FALSE | 0.65 | 16479.38 | 0 | TRUE | 0.65 | -1.07 | -0.03 | -0.09 | -0.05 |
| TCGA-FD-A43N-01 | TRUE | FALSE | 0.64 | -1330.76 | 0 | FALSE | 0 | 0.64 | 0.02 | 0.09 | 0 |
| TCGA-XF-AAMG-01 | TRUE | FALSE | 0.62 | -1797.78 | 0 | FALSE | -0.23 | 0.62 | 0.01 | 0.08 | 0.01 |
| TCGA-XF-A8HF-01 | TRUE | FALSE | 0.6 | -1027.79 | 0 | FALSE | -0.03 | 0.6 | 0.01 | 0.05 | 0.02 |
| TCGA-E7-A7DV-01 | FALSE | FALSE | 0.59 | 15356.9 | 0 | FALSE | 0.12 | 0.59 | 0 | 0.12 | -0.02 |
| TCGA-4Z-AA7N-01 | FALSE | FALSE | 0.58 | 8811.36 | 0 | TRUE | 0.58 | 0.59 | -0.05 | 0.15 | -0.01 |
| TCGA-XF-A9ST-01 | TRUE | FALSE | 0.58 | -3237.62 | nan | FALSE | -0.73 | 0.58 | 0.1 | -0.05 | 0.04 |
| TCGA-FD-A43Y-01 | FALSE | FALSE | 0.56 | 7468.65 | 0 | FALSE | 0.15 | 0.56 | 0.01 | 0.09 | -0.01 |
| TCGA-FD-A62N-01 | FALSE | FALSE | 0.56 | 56004.18 | 0 | TRUE | 0.56 | 0.6 | -0.01 | 0.14 | -0.04 |
| TCGA-XF-A9T6-01 | FALSE | FALSE | 0.56 | 329.78 | nan | FALSE | -0.18 | 0.56 | 0.1 | -0.03 | 0 |
| TCGA-FD-A6TB-01 | FALSE | FALSE | 0.56 | 7412.73 | 0 | TRUE | 0.56 | 0.44 | -0.06 | 0.16 | -0.02 |
| TCGA-FD-A5BU-01 | FALSE | FALSE | 0.55 | 3213.11 | 0 | FALSE | 0.11 | 0.55 | 0 | 0.1 | -0.01 |
| TCGA-CF-A9FL-01 | TRUE | FALSE | 0.55 | -2379.96 | nan | FALSE | -0.04 | 0.55 | 0.02 | 0.05 | 0.02 |
| TCGA-DK-A3WY-01 | FALSE | FALSE | 0.55 | 24233.42 | 1 | TRUE | 0.55 | -0.29 | -0.03 | -0.02 | 0.01 |
| TCGA-CF-A8HY-01 | TRUE | FALSE | 0.54 | -3152.54 | nan | FALSE | -0.08 | 0.54 | 0.06 | 0 | 0.01 |
| TCGA-FD-A5BS-01 | FALSE | FALSE | 0.54 | 38589.62 | 0 | TRUE | 0.54 | 0.81 | -0.02 | 0.18 | -0.05 |
| TCGA-K4-A83P-01 | FALSE | FALSE | 0.54 | 8706.16 | 0 | TRUE | 0.54 | 0.89 | -0.05 | 0.21 | -0.02 |
| TCGA-2F-A9KT-01 | TRUE | FALSE | 0.53 | -2644.91 | 0 | FALSE | 0.07 | 0.53 | 0.03 | 0.05 | 0 |
| TCGA-FD-A3NA-01 | FALSE | FALSE | 0.52 | 6328.68 | 0 | FALSE | -0.02 | 0.52 | 0.02 | 0.08 | -0.02 |
| TCGA-FD-A43S-01 | FALSE | FALSE | 0.52 | 3219.62 | nan | TRUE | 0.52 | 0.98 | 0 | 0.14 | 0.01 |
| TCGA-UY-A9PE-01 | TRUE | FALSE | 0.51 | -2343.9 | 1 | FALSE | -0.18 | 0.51 | 0.03 | 0.02 | 0.03 |
| TCGA-FD-A3B4-01 | FALSE | FALSE | 0.51 | 6871.87 | 0 | FALSE | 0.13 | 0.51 | 0.01 | 0.08 | -0.02 |
| TCGA-E7-A6MD-01 | TRUE | FALSE | 0.51 | -263.68 | 0 | FALSE | 0.21 | 0.51 | -0.03 | 0.1 | 0.01 |
| TCGA-DK-A1AD-01 | FALSE | FALSE | 0.51 | 959.64 | 0 | FALSE | -0.23 | 0.51 | 0.02 | 0.04 | 0.02 |
| TCGA-BT-A0S7-01 | TRUE | FALSE | 0.5 | -1844.31 | nan | FALSE | -0.27 | 0.5 | 0.04 | 0.01 | 0.02 |
| TCGA-4Z-AA84-01 | TRUE | FALSE | 0.49 | -1379.56 | nan | FALSE | -0.41 | 0.49 | 0.06 | -0.01 | 0.01 |
| TCGA-GU-A762-01 | FALSE | FALSE | 0.47 | 20918.6 | 0 | TRUE | 0.47 | 0.26 | 0.02 | 0.07 | -0.05 |
| TCGA-KQ-A41N-01 | TRUE | FALSE | 0.47 | -3369.16 | 1 | FALSE | 0.02 | 0.47 | 0.06 | -0.01 | 0.02 |
| TCGA-FD-A3B5-01 | TRUE | FALSE | 0.47 | -1674.46 | 1 | FALSE | 0.03 | 0.47 | 0.02 | 0.05 | -0.01 |
| TCGA-XF-A9SH-01 | TRUE | FALSE | 0.47 | -2146.82 | nan | FALSE | 0.01 | 0.47 | 0.02 | -0.02 | 0.06 |
| TCGA-DK-A3IL-01 | TRUE | FALSE | 0.47 | -2941.55 | 0 | FALSE | 0.21 | 0.47 | 0.02 | 0.03 | 0.03 |
| TCGA-BT-A2LD-01 | FALSE | FALSE | 0.46 | 35.01 | 0 | FALSE | 0.11 | 0.46 | 0.03 | 0.06 | -0.02 |
| TCGA-FD-A5BR-01 | TRUE | FALSE | 0.46 | -185.63 | 1 | FALSE | 0.29 | 0.46 | 0 | 0.05 | 0.02 |
| TCGA-FD-A5BV-01 | TRUE | FALSE | 0.44 | -3119.3 | nan | FALSE | -0.32 | 0.44 | 0.04 | -0.01 | 0.04 |
| TCGA-DK-A2I4-01 | FALSE | FALSE | 0.44 | 37855.66 | 0 | TRUE | 0.44 | 0.29 | -0.03 | 0.15 | -0.07 |
| TCGA-UY-A9PA-01 | FALSE | FALSE | 0.44 | 14615.44 | 0 | TRUE | 0.44 | -0.59 | 0 | -0.01 | -0.09 |
| TCGA-CF-A5UA-01 | TRUE | FALSE | 0.43 | -3012.26 | nan | FALSE | -0.05 | 0.43 | 0.05 | -0.02 | 0.02 |
| TCGA-CF-A47T-01 | TRUE | FALSE | 0.43 | -3450.06 | nan | FALSE | -0.08 | 0.43 | 0.04 | 0 | 0.02 |
| TCGA-GU-A766-01 | FALSE | FALSE | 0.42 | 46829.15 | 0 | TRUE | 0.42 | -0.22 | 0.02 | 0.04 | -0.1 |
| TCGA-DK-A3X1-01 | TRUE | FALSE | 0.41 | -1928.84 | 0 | FALSE | -0.18 | 0.41 | 0.02 | 0.02 | 0.02 |
| TCGA-FD-A5BX-01 | FALSE | FALSE | 0.41 | 1376 | 0 | FALSE | 0.06 | 0.41 | -0.03 | 0.1 | -0.01 |
| TCGA-C4-A0F1-01 | FALSE | FALSE | 0.4 | 2818.44 | nan | FALSE | -0.09 | 0.4 | 0.03 | 0.02 | 0.01 |
| TCGA-ZF-AA56-01 | FALSE | FALSE | 0.4 | 2822.19 | 0 | FALSE | 0.14 | 0.4 | 0.01 | 0.08 | -0.02 |
| TCGA-GU-A763-01 | TRUE | FALSE | 0.39 | -2209.5 | nan | FALSE | -0.09 | 0.39 | 0.05 | -0.02 | 0.02 |
| TCGA-E7-A97Q-01 | TRUE | FALSE | 0.39 | -684.79 | 0 | FALSE | 0.21 | 0.39 | -0.01 | 0.07 | 0 |
| TCGA-GD-A2C5-01 | TRUE | FALSE | 0.39 | -1829.75 | 0 | FALSE | 0 | 0.39 | -0.01 | 0.07 | 0.01 |
| TCGA-UY-A78N-01 | TRUE | FALSE | 0.38 | -2943.46 | nan | FALSE | -0.12 | 0.38 | 0.03 | 0 | 0.02 |
| TCGA-SY-A9G5-01 | FALSE | FALSE | 0.38 | 13653.01 | 0 | TRUE | 0.38 | 1.21 | 0.01 | 0.21 | -0.03 |
| TCGA-GD-A6C6-01 | TRUE | FALSE | 0.37 | -1901.79 | nan | FALSE | -0.01 | 0.37 | 0.06 | -0.01 | 0.01 |
| TCGA-YF-AA3M-01 | TRUE | FALSE | 0.37 | -1700.85 | 0 | FALSE | -0.02 | 0.37 | 0.05 | -0.03 | 0.02 |
| TCGA-BT-A20U-01 | FALSE | FALSE | 0.37 | 1877.12 | nan | FALSE | -0.08 | 0.37 | 0 | 0.06 | 0 |
| TCGA-FD-A43U-01 | FALSE | FALSE | 0.37 | 6687.21 | 0 | TRUE | 0.37 | 1.2 | -0.01 | 0.21 | -0.01 |
| TCGA-DK-A3IK-01 | TRUE | FALSE | 0.36 | -2313.55 | 0 | FALSE | 0.01 | 0.36 | -0.01 | 0.04 | 0.02 |
| TCGA-XF-A9SX-01 | FALSE | FALSE | 0.36 | 12012.01 | 0 | TRUE | 0.36 | 1.29 | 0.03 | 0.19 | -0.03 |
| TCGA-XF-A8HH-01 | TRUE | FALSE | 0.36 | -1204.36 | 0 | FALSE | -0.01 | 0.36 | 0 | 0.05 | 0 |
| TCGA-ZF-A9R0-01 | FALSE | FALSE | 0.36 | 79.65 | 1 | FALSE | 0.24 | 0.36 | 0.01 | 0.03 | 0.01 |
| TCGA-E7-A8O8-01 | TRUE | FALSE | 0.35 | -2234 | nan | FALSE | -0.11 | 0.35 | 0.05 | -0.04 | 0.03 |
| TCGA-XF-A9SM-01 | FALSE | FALSE | 0.35 | 33945.95 | 0 | TRUE | 0.35 | 0.51 | -0.01 | 0.13 | -0.05 |
| TCGA-DK-AA74-01 | FALSE | FALSE | 0.35 | 13007.18 | 0 | TRUE | 0.35 | 1.43 | 0.01 | 0.24 | -0.03 |
| TCGA-E7-A541-01 | FALSE | FALSE | 0.35 | 4498.67 | 0 | TRUE | 0.35 | -0.63 | -0.01 | -0.05 | -0.04 |
| TCGA-DK-A6AW-01 | TRUE | FALSE | 0.34 | -1218.99 | nan | FALSE | 0.08 | 0.34 | 0.05 | 0 | 0 |
| TCGA-FD-A3B8-01 | FALSE | FALSE | 0.34 | 31559.05 | 0 | TRUE | 0.34 | 1.42 | -0.01 | 0.27 | -0.04 |
| TCGA-BT-A2LA-01 | TRUE | FALSE | 0.34 | -2989.88 | 1 | FALSE | -0.2 | 0.34 | 0.06 | -0.04 | 0.02 |
| TCGA-2F-A9KW-01 | TRUE | FALSE | 0.33 | -1090.17 | 0 | FALSE | -0.15 | 0.33 | 0.01 | 0.05 | -0.01 |
| TCGA-BT-A20Q-01 | FALSE | FALSE | 0.33 | 5706.17 | 0 | TRUE | 0.33 | 1.22 | -0.02 | 0.23 | -0.02 |
| TCGA-R3-A69X-01 | FALSE | FALSE | 0.33 | 3520.26 | 0 | TRUE | 0.33 | 0.73 | -0.06 | 0.19 | 0 |
| TCGA-BT-A20O-01 | FALSE | FALSE | 0.33 | 45661.44 | 0 | TRUE | 0.33 | 0 | -0.04 | 0.1 | -0.05 |
| TCGA-CF-A7I0-01 | TRUE | FALSE | 0.33 | -2645.87 | nan | FALSE | 0.11 | 0.33 | 0.01 | 0.01 | 0.03 |
| TCGA-UY-A78O-01 | TRUE | FALSE | 0.32 | -2102.84 | nan | FALSE | -0.07 | 0.32 | 0.04 | -0.02 | 0.02 |
| TCGA-XF-A9SI-01 | FALSE | FALSE | 0.32 | 13484.07 | 0 | TRUE | 0.32 | 0.02 | -0.03 | 0.02 | -0.02 |
| TCGA-DK-A3IU-01 | FALSE | FALSE | 0.32 | 28869.43 | 0 | TRUE | 0.32 | 0.58 | 0.01 | 0.11 | -0.03 |
| TCGA-YC-A9TC-01 | TRUE | FALSE | 0.32 | -2136.77 | nan | FALSE | -0.31 | 0.32 | 0.04 | -0.03 | 0.02 |
| TCGA-KQ-A41O-01 | TRUE | FALSE | 0.32 | -3609.64 | nan | FALSE | -0.17 | 0.32 | 0.07 | -0.06 | 0.04 |
| TCGA-E7-A519-01 | TRUE | FALSE | 0.31 | -1615.76 | nan | FALSE | 0.15 | 0.31 | 0.04 | -0.01 | 0.01 |
| TCGA-FD-A6TK-01 | FALSE | FALSE | 0.3 | 9783.74 | 0 | TRUE | 0.3 | 1.45 | 0.03 | 0.22 | -0.03 |
| TCGA-E7-A3X6-01 | FALSE | FALSE | 0.3 | 18403.59 | 0 | TRUE | 0.3 | -0.2 | 0.01 | 0.02 | -0.05 |
| TCGA-ZF-AA58-01 | FALSE | FALSE | 0.3 | 24695.44 | 0 | TRUE | 0.3 | 0.73 | 0.02 | 0.15 | -0.06 |
| TCGA-FT-A61P-01 | FALSE | FALSE | 0.29 | 25456.65 | 0 | TRUE | 0.29 | 1.09 | 0.04 | 0.18 | -0.05 |
| TCGA-GC-A3RD-01 | FALSE | FALSE | 0.29 | -2026.65 | 0 | FALSE | -0.04 | 0.29 | 0.03 | 0.01 | 0 |
| TCGA-2F-A9KQ-01 | FALSE | FALSE | 0.28 | -3229.65 | nan | FALSE | -0.05 | 0.28 | 0.02 | 0.01 | 0.01 |
| TCGA-UY-A9PB-01 | FALSE | FALSE | 0.28 | 19188.18 | 0 | TRUE | 0.28 | 1.22 | 0.02 | 0.21 | -0.04 |
| TCGA-E7-A7XN-01 | FALSE | FALSE | 0.28 | 41489.37 | nan | TRUE | 0.28 | -0.46 | 0.01 | -0.02 | -0.06 |
| TCGA-K4-A5RH-01 | FALSE | FALSE | 0.28 | 39024.45 | 0 | TRUE | 0.28 | 0.74 | -0.01 | 0.17 | -0.04 |
| TCGA-FD-A5BT-01 | FALSE | FALSE | 0.28 | 10880.12 | 0 | TRUE | 0.28 | 1.64 | 0.03 | 0.24 | -0.02 |
| TCGA-XF-A8HD-01 | FALSE | FALSE | 0.28 | 33775.22 | 0 | TRUE | 0.28 | 0.23 | 0.02 | 0.08 | -0.07 |
| TCGA-UY-A9PH-01 | FALSE | FALSE | 0.27 | 20790.53 | 0 | TRUE | 0.27 | -0.18 | -0.01 | 0 | -0.03 |
| TCGA-S5-AA26-01 | FALSE | FALSE | 0.27 | -3599.47 | nan | FALSE | -0.04 | 0.27 | 0.06 | -0.06 | 0.03 |
| TCGA-FD-A6TI-01 | FALSE | FALSE | 0.27 | 516.65 | nan | FALSE | 0.15 | 0.27 | 0.01 | 0.07 | -0.04 |
| TCGA-CU-A72E-01 | FALSE | FALSE | 0.26 | -1426.98 | nan | FALSE | 0.09 | 0.26 | 0 | 0.05 | -0.01 |
| TCGA-GV-A3JZ-01 | FALSE | FALSE | 0.26 | -588.39 | 0 | FALSE | -0.24 | 0.26 | 0.01 | 0.03 | 0.01 |
| TCGA-FJ-A3Z7-01 | FALSE | FALSE | 0.25 | -2487.24 | 0 | FALSE | -0.13 | 0.25 | 0.01 | 0.05 | -0.03 |
| TCGA-DK-AA71-01 | FALSE | FALSE | 0.25 | -857.9 | nan | FALSE | -0.01 | 0.25 | 0.04 | -0.03 | 0.01 |
| TCGA-DK-A3IM-01 | FALSE | FALSE | 0.25 | -2018.47 | nan | FALSE | -0.21 | 0.25 | 0.04 | -0.02 | 0.01 |
| TCGA-DK-AA75-01 | FALSE | FALSE | 0.25 | -2842.74 | 0 | FALSE | -0.27 | 0.25 | 0.06 | -0.07 | 0.04 |
| TCGA-FD-A5C1-01 | FALSE | FALSE | 0.25 | 31771.22 | 0 | TRUE | 0.25 | 0.4 | 0.02 | 0.11 | -0.06 |
| TCGA-DK-A1AF-01 | FALSE | FALSE | 0.25 | 4303.42 | 0 | TRUE | 0.25 | 1.75 | -0.01 | 0.3 | -0.02 |
| TCGA-BT-A2LB-01 | FALSE | FALSE | 0.24 | 4298.3 | 0 | TRUE | 0.24 | 1.12 | 0 | 0.19 | -0.02 |
| TCGA-K4-A5RJ-01 | FALSE | FALSE | 0.24 | 38774.25 | 0 | TRUE | 0.24 | -0.51 | -0.07 | 0.03 | -0.04 |
| TCGA-E7-A97P-01 | FALSE | FALSE | 0.23 | 33067.23 | nan | TRUE | 0.23 | 0.41 | 0.03 | 0.1 | -0.07 |
| TCGA-CF-A3MH-01 | FALSE | FALSE | 0.23 | -2838.67 | nan | FALSE | 0.08 | 0.23 | 0.04 | -0.02 | 0.01 |
| TCGA-UY-A9PF-01 | FALSE | FALSE | 0.23 | -306.47 | 1 | FALSE | -0.01 | 0.23 | 0 | 0.01 | 0.02 |
| TCGA-FD-A3B6-01 | FALSE | FALSE | 0.22 | 17105.32 | 0 | TRUE | 0.22 | 0.3 | 0.03 | 0.04 | -0.03 |
| TCGA-FD-A3SJ-01 | FALSE | FALSE | 0.22 | -972.25 | 0 | FALSE | -0.15 | 0.22 | -0.01 | 0.02 | 0.01 |
| TCGA-K4-A5RI-01 | FALSE | FALSE | 0.21 | -393.02 | 0 | FALSE | -0.08 | 0.21 | 0 | 0.04 | -0.01 |
| TCGA-4Z-AA86-01 | FALSE | FALSE | 0.21 | 12122.29 | 0 | TRUE | 0.21 | 1.19 | 0.02 | 0.2 | -0.04 |
| TCGA-FD-A3N5-01 | FALSE | FALSE | 0.21 | 8704.37 | nan | FALSE | 0.06 | 0.21 | 0.02 | 0.01 | -0.01 |
| TCGA-UY-A78P-01 | FALSE | FALSE | 0.21 | 17991.37 | 0 | TRUE | 0.21 | 0.01 | 0 | 0.03 | -0.03 |
| TCGA-CF-A1HS-01 | FALSE | FALSE | 0.21 | 1879.91 | 0 | FALSE | -0.05 | 0.21 | 0.02 | 0.01 | 0 |
| TCGA-XF-AAN4-01 | FALSE | FALSE | 0.21 | 8307.53 | 1 | TRUE | 0.21 | 1.13 | -0.01 | 0.19 | 0 |
| TCGA-XF-AAMQ-01 | FALSE | FALSE | 0.21 | 13222.56 | 0 | TRUE | 0.21 | -0.07 | 0.04 | -0.01 | -0.04 |
| TCGA-FD-A3SP-01 | FALSE | FALSE | 0.2 | 8677.88 | 0 | TRUE | 0.2 | 1.95 | 0.01 | 0.31 | -0.02 |
| TCGA-4Z-AA83-01 | FALSE | FALSE | 0.2 | 3166.22 | 0 | FALSE | 0.33 | 0.2 | 0.01 | 0.05 | -0.03 |
| TCGA-GU-A42Q-01 | FALSE | FALSE | 0.2 | -1034.28 | nan | FALSE | -0.08 | 0.2 | 0.01 | 0.02 | -0.01 |
| TCGA-DK-A1AA-01 | FALSE | FALSE | 0.2 | -1716.07 | nan | FALSE | -0.01 | 0.2 | 0.02 | 0 | 0 |
| TCGA-BT-A20T-01 | FALSE | FALSE | 0.2 | 6757.33 | 0 | TRUE | 0.2 | 0.95 | 0.02 | 0.14 | -0.01 |
| TCGA-FD-A3B7-01 | FALSE | FALSE | 0.19 | 10444.71 | 0 | TRUE | 0.19 | 1.52 | 0.02 | 0.23 | -0.02 |
| TCGA-DK-AA6R-01 | FALSE | FALSE | 0.19 | 1734.14 | 1 | FALSE | 0.01 | 0.19 | 0.02 | 0.02 | -0.01 |
| TCGA-FD-A3SS-01 | FALSE | FALSE | 0.18 | -2065.27 | 0 | FALSE | -0.06 | 0.18 | 0 | 0.03 | 0 |
| TCGA-E7-A7DU-01 | FALSE | FALSE | 0.18 | -2801.21 | 0 | FALSE | 0.15 | 0.18 | 0.04 | -0.03 | 0.01 |
| TCGA-5N-A9KI-01 | FALSE | FALSE | 0.18 | 817.71 | 0 | FALSE | 0.12 | 0.18 | 0.02 | 0.05 | -0.04 |
| TCGA-XF-A9SY-01 | FALSE | FALSE | 0.18 | 25809 | 0 | TRUE | 0.18 | 1.37 | 0.05 | 0.21 | -0.04 |
| TCGA-DK-A1AE-01 | FALSE | FALSE | 0.18 | -370.43 | nan | FALSE | -0.33 | 0.18 | 0.02 | 0 | 0.01 |
| TCGA-CF-A47W-01 | FALSE | FALSE | 0.17 | -3121.46 | nan | FALSE | 0.06 | 0.17 | 0.03 | -0.03 | 0.01 |
| TCGA-DK-A6B2-01 | FALSE | FALSE | 0.17 | 5236.32 | 0 | TRUE | 0.17 | 0.89 | -0.02 | 0.19 | -0.02 |
| TCGA-BT-A3PH-01 | FALSE | FALSE | 0.17 | -3286.61 | nan | FALSE | -0.08 | 0.17 | 0 | 0.02 | 0 |
| TCGA-H4-A2HO-01 | FALSE | FALSE | 0.16 | -1339.12 | nan | FALSE | -0.08 | 0.16 | -0.01 | 0.02 | 0.02 |
| TCGA-UY-A78L-01 | FALSE | FALSE | 0.16 | 5417 | 0 | FALSE | -0.11 | 0.16 | 0.01 | 0.06 | -0.04 |
| TCGA-FD-A3B3-01 | FALSE | FALSE | 0.16 | 15912.61 | 0 | TRUE | 0.16 | 0.97 | 0.02 | 0.17 | -0.03 |
| TCGA-DK-A3WX-01 | FALSE | FALSE | 0.16 | 3601.38 | 0 | TRUE | 0.16 | 1.08 | 0.01 | 0.17 | -0.01 |
| TCGA-GC-A3RC-01 | FALSE | FALSE | 0.16 | 10207.27 | 0 | TRUE | 0.16 | 0.23 | 0.01 | 0.03 | -0.01 |
| TCGA-S5-A6DX-01 | FALSE | FALSE | 0.16 | 5099.82 | 1 | TRUE | 0.16 | 0.35 | 0.03 | 0.02 | 0 |
| TCGA-ZF-AA5H-01 | FALSE | FALSE | 0.16 | 9815.89 | 0 | TRUE | 0.16 | 0.52 | 0.04 | 0.09 | -0.04 |
| TCGA-G2-A3IE-01 | FALSE | FALSE | 0.15 | -2774.54 | 0 | FALSE | 0 | 0.15 | 0.02 | -0.01 | 0.01 |
| TCGA-CU-A0YO-01 | FALSE | FALSE | 0.15 | 5987.4 | nan | FALSE | 0.27 | 0.15 | 0 | 0.03 | -0.01 |
| TCGA-XF-A9T5-01 | FALSE | FALSE | 0.15 | 44967.3 | 1 | TRUE | 0.15 | -0.19 | 0.01 | -0.01 | -0.03 |
| TCGA-DK-AA6T-01 | FALSE | FALSE | 0.14 | 18082.61 | nan | TRUE | 0.14 | -0.53 | -0.02 | -0.11 | 0.04 |
| TCGA-GC-A3RB-01 | FALSE | FALSE | 0.14 | -383.19 | 0 | FALSE | -0.27 | 0.14 | 0.06 | -0.04 | 0 |
| TCGA-UY-A78K-01 | FALSE | FALSE | 0.14 | 8858.24 | 0 | TRUE | 0.14 | -0.46 | -0.01 | -0.06 | 0.01 |
| TCGA-XF-A9SU-01 | FALSE | FALSE | 0.13 | -488.61 | nan | TRUE | 0.13 | 1.42 | 0.04 | 0.16 | 0.02 |
| TCGA-2F-A9KO-01 | FALSE | FALSE | 0.13 | 11279.61 | 0 | TRUE | 0.13 | -0.04 | 0.01 | 0.04 | -0.05 |
| TCGA-DK-AA6L-01 | FALSE | FALSE | 0.13 | 3329.03 | 0 | FALSE | 0.25 | 0.13 | 0.01 | 0.02 | -0.01 |
| TCGA-FJ-A3Z9-01 | FALSE | FALSE | 0.13 | -3167.46 | nan | FALSE | -0.1 | 0.13 | 0.03 | -0.03 | 0.02 |
| TCGA-GC-A6I1-01 | FALSE | FALSE | 0.13 | 18165.59 | 0 | TRUE | 0.13 | 0 | 0.01 | 0.02 | -0.03 |
| TCGA-FD-A43X-01 | FALSE | FALSE | 0.12 | -3055.82 | 0 | FALSE | 0.2 | 0.12 | 0.04 | -0.03 | 0 |
| TCGA-E7-A678-01 | FALSE | FALSE | 0.12 | -2270.92 | nan | FALSE | 0.07 | 0.12 | 0.03 | -0.03 | 0.02 |
| TCGA-FD-A62P-01 | FALSE | FALSE | 0.12 | 3065.19 | 0 | TRUE | 0.12 | 0.55 | -0.01 | 0.12 | -0.02 |
| TCGA-XF-A9SJ-01 | FALSE | FALSE | 0.12 | 8682.97 | 0 | TRUE | 0.12 | 0.36 | 0.01 | 0.09 | -0.04 |
| TCGA-GD-A3OP-01 | FALSE | FALSE | 0.11 | 279.94 | 0 | FALSE | 0.11 | 0.11 | -0.03 | 0.05 | 0 |
| TCGA-FD-A3SR-01 | FALSE | FALSE | 0.11 | 4099.8 | 0 | FALSE | 0.19 | 0.11 | -0.04 | 0.11 | -0.04 |
| TCGA-YF-AA3L-01 | FALSE | FALSE | 0.11 | -2209.48 | 0 | FALSE | 0.07 | 0.11 | 0.02 | -0.02 | 0.01 |
| TCGA-CF-A3MI-01 | FALSE | FALSE | 0.11 | -2427 | 0 | FALSE | 0.06 | 0.11 | 0.01 | 0 | 0 |
| TCGA-XF-A8HG-01 | FALSE | FALSE | 0.1 | -3215.58 | nan | FALSE | -0.23 | 0.1 | 0.01 | -0.02 | 0.02 |
| TCGA-K4-A54R-01 | FALSE | FALSE | 0.1 | 10771.24 | nan | TRUE | 0.1 | 1.36 | 0.01 | 0.21 | -0.02 |
| TCGA-G2-AA3C-01 | FALSE | FALSE | 0.1 | 687.58 | 0 | TRUE | 0.1 | 0.8 | -0.01 | 0.13 | 0 |
| TCGA-E7-A4XJ-01 | FALSE | FALSE | 0.1 | -3354.39 | nan | FALSE | -0.08 | 0.1 | 0.02 | -0.02 | 0.01 |
| TCGA-4Z-AA81-01 | FALSE | FALSE | 0.09 | 19168.9 | 0 | TRUE | 0.09 | -0.13 | 0.05 | -0.03 | -0.04 |
| TCGA-4Z-AA7W-01 | FALSE | FALSE | 0.09 | 31841.63 | 0 | TRUE | 0.09 | -0.37 | 0 | -0.01 | -0.05 |
| TCGA-C4-A0F7-01 | FALSE | FALSE | 0.09 | -1683.76 | nan | FALSE | -0.12 | 0.09 | 0.01 | -0.01 | 0 |
| TCGA-FD-A5C0-01 | FALSE | FALSE | 0.09 | -1516.7 | 0 | FALSE | -0.07 | 0.09 | -0.02 | 0.05 | -0.01 |
| TCGA-4Z-AA7R-01 | FALSE | FALSE | 0.09 | -1639.24 | 0 | FALSE | -0.2 | 0.09 | 0 | 0 | 0.01 |
| TCGA-XF-A9T8-01 | FALSE | FALSE | 0.09 | 19523.83 | nan | TRUE | 0.09 | 0.08 | 0.01 | 0.06 | -0.06 |
| TCGA-XF-AAN1-01 | FALSE | FALSE | 0.09 | -1189.88 | nan | FALSE | -0.03 | 0.09 | 0.03 | -0.04 | 0.01 |
| TCGA-K4-A3WU-01 | FALSE | FALSE | 0.09 | 784.76 | 0 | FALSE | 0.21 | 0.09 | -0.04 | 0.05 | 0.01 |
| TCGA-G2-A3IB-01 | FALSE | FALSE | 0.09 | -651.93 | nan | FALSE | 0.04 | 0.09 | 0.01 | 0 | 0 |
| TCGA-DK-AA6Q-01 | FALSE | FALSE | 0.08 | 3676.7 | 0 | TRUE | 0.08 | -0.17 | -0.01 | 0 | -0.02 |
| TCGA-DK-A6B0-01 | FALSE | FALSE | 0.08 | -2472.93 | nan | FALSE | 0.12 | 0.08 | 0.02 | -0.03 | 0.01 |
| TCGA-ZF-AA53-01 | FALSE | FALSE | 0.08 | 9546.42 | 0 | TRUE | 0.08 | 0.77 | 0.01 | 0.14 | -0.03 |
| TCGA-XF-A8HE-01 | FALSE | FALSE | 0.07 | 5842.26 | nan | TRUE | 0.07 | 0.15 | 0.02 | 0.04 | -0.03 |
| TCGA-UY-A8OB-01 | FALSE | FALSE | 0.07 | 18381.25 | 0 | TRUE | 0.07 | 0.4 | 0.05 | 0.03 | -0.02 |
| TCGA-BT-A20P-01 | FALSE | FALSE | 0.07 | -1930.8 | 1 | FALSE | 0.16 | 0.07 | 0.04 | -0.01 | -0.02 |
| TCGA-ZF-A9RD-01 | FALSE | FALSE | 0.07 | 17904.55 | nan | TRUE | 0.07 | 0.05 | 0.02 | 0.01 | -0.03 |
| TCGA-E7-A3Y1-01 | FALSE | FALSE | 0.07 | -2804.49 | nan | FALSE | 0.08 | 0.07 | 0.01 | -0.01 | 0.01 |
| TCGA-CF-A47Y-01 | FALSE | FALSE | 0.07 | -3008.09 | nan | FALSE | 0.12 | 0.07 | 0.02 | -0.03 | 0.01 |
| TCGA-GV-A3QK-01 | FALSE | FALSE | 0.07 | 729.9 | nan | TRUE | 0.07 | -0.36 | 0.01 | -0.06 | -0.01 |
| TCGA-E7-A677-01 | FALSE | FALSE | 0.07 | -984.53 | 0 | FALSE | 0.29 | 0.07 | 0 | 0.05 | -0.03 |
| TCGA-C4-A0EZ-01 | FALSE | FALSE | 0.07 | -2891.71 | 0 | FALSE | -0.27 | 0.07 | 0.05 | -0.06 | 0.02 |
| TCGA-G2-A2EF-01 | FALSE | FALSE | 0.06 | 12063.43 | 0 | TRUE | 0.06 | 0.48 | 0.05 | 0.07 | -0.05 |
| TCGA-4Z-AA7M-01 | FALSE | FALSE | 0.06 | -2896.41 | 0 | FALSE | -0.16 | 0.06 | 0 | 0 | 0.01 |
| TCGA-GU-AATO-01 | FALSE | FALSE | 0.06 | 5787.07 | 0 | TRUE | 0.06 | -0.07 | 0 | 0.02 | -0.03 |
| TCGA-DK-A3WW-01 | FALSE | FALSE | 0.06 | 24882.79 | nan | TRUE | 0.06 | 0.13 | 0.03 | 0.01 | -0.03 |
| TCGA-GC-A3WC-01 | FALSE | FALSE | 0.06 | 23420.3 | 0 | TRUE | 0.06 | -0.46 | -0.03 | -0.01 | -0.03 |
| TCGA-5N-A9KM-01 | FALSE | FALSE | 0.05 | 6699.35 | 0 | TRUE | 0.05 | 0.42 | -0.01 | 0.08 | 0 |
| TCGA-DK-A3X2-01 | FALSE | FALSE | 0.05 | -2724.36 | nan | FALSE | -0.13 | 0.05 | 0.04 | -0.06 | 0.02 |
| TCGA-BT-A0YX-01 | FALSE | FALSE | 0.04 | 2093.7 | nan | TRUE | 0.04 | -0.14 | 0 | 0 | -0.02 |
| TCGA-CF-A9FF-01 | FALSE | FALSE | 0.04 | -930.81 | nan | FALSE | 0.05 | 0.04 | 0.01 | -0.01 | 0.01 |
| TCGA-4Z-AA7O-01 | FALSE | FALSE | 0.04 | 3526.24 | 1 | TRUE | 0.04 | 0.38 | 0.04 | 0.02 | 0 |
| TCGA-CF-A3MF-01 | FALSE | FALSE | 0.04 | -3437.31 | 0 | FALSE | 0.18 | 0.04 | 0.01 | -0.01 | 0.01 |
| TCGA-2F-A9KP-01 | FALSE | FALSE | 0.04 | -2956.41 | nan | FALSE | -0.13 | 0.04 | 0.03 | -0.04 | 0.01 |
| TCGA-XF-AAMZ-01 | FALSE | FALSE | 0.03 | -1784.72 | 0.01 | FALSE | -0.09 | 0.03 | 0.03 | -0.04 | 0.02 |
| TCGA-FD-A43P-01 | FALSE | FALSE | 0.03 | 10516.77 | 0 | TRUE | 0.03 | -0.3 | -0.01 | -0.02 | -0.01 |
| TCGA-KQ-A41R-01 | FALSE | FALSE | 0.03 | -3142.82 | nan | FALSE | -0.05 | 0.03 | 0.03 | -0.05 | 0.02 |
| TCGA-XF-AAN2-01 | FALSE | FALSE | 0.03 | 13037.61 | 1 | TRUE | 0.03 | -0.34 | 0.03 | -0.03 | -0.06 |
| TCGA-DK-A1AG-01 | FALSE | FALSE | 0.03 | -2625.41 | nan | FALSE | 0.17 | 0.03 | 0.04 | -0.05 | 0.01 |
| TCGA-ZF-AA4U-01 | FALSE | FALSE | 0.03 | 945.11 | nan | FALSE | -0.08 | 0.03 | 0.03 | -0.04 | 0.01 |
| TCGA-HQ-A5ND-01 | FALSE | FALSE | 0.03 | 247.84 | nan | FALSE | -0.2 | 0.03 | 0.06 | -0.03 | -0.02 |
| TCGA-K4-A4AC-01 | FALSE | FALSE | 0.02 | 5359.88 | 0 | TRUE | 0.02 | 0.26 | 0.02 | 0.02 | -0.01 |
| TCGA-BT-A42E-01 | FALSE | FALSE | 0.02 | 10702.16 | 1 | TRUE | 0.02 | 0.28 | 0.03 | 0.03 | -0.02 |
| TCGA-BT-A3PJ-01 | FALSE | FALSE | 0.02 | 15648.14 | 0 | TRUE | 0.02 | 0.34 | 0.04 | 0.07 | -0.05 |
| TCGA-G2-A2EJ-01 | FALSE | FALSE | 0.02 | 8082.17 | 0 | TRUE | 0.02 | -0.06 | 0.03 | 0 | -0.05 |
| TCGA-GC-A6I3-01 | FALSE | FALSE | 0.02 | 5083.1 | 0 | TRUE | 0.02 | 0.37 | 0.04 | -0.01 | 0.02 |
| TCGA-GC-A3I6-01 | FALSE | FALSE | 0.01 | 17607.17 | 0 | FALSE | 0.34 | 0.01 | 0 | 0.05 | -0.05 |
| TCGA-GV-A3JV-01 | FALSE | FALSE | 0.01 | 1910.18 | 0 | TRUE | 0.01 | 0.35 | 0.02 | 0.04 | 0 |
| TCGA-E5-A2PC-01 | FALSE | FALSE | 0.01 | 8554.96 | 0 | TRUE | 0.01 | -0.22 | 0.01 | 0 | -0.04 |
| TCGA-LC-A66R-01 | FALSE | FALSE | 0.01 | 8383.88 | 0 | TRUE | 0.01 | 0.1 | 0.04 | 0.02 | -0.04 |
| TCGA-LT-A8JT-01 | FALSE | FALSE | 0.01 | -1474.41 | nan | FALSE | -0.02 | 0.01 | 0.02 | -0.05 | 0.02 |
| TCGA-4Z-AA7Q-01 | FALSE | FALSE | 0.01 | 11878.46 | 0 | TRUE | 0.01 | -0.57 | -0.02 | -0.01 | -0.06 |
| TCGA-GU-AATQ-01 | FALSE | FALSE | 0 | 1292.07 | 0 | FALSE | -0.02 | 0 | 0.01 | 0 | -0.01 |
| TCGA-CF-A5U8-01 | FALSE | TRUE | -0.01 | -2968.93 | nan | FALSE | 0.08 | -0.01 | 0.01 | -0.04 | 0.02 |
| TCGA-BT-A42F-01 | FALSE | TRUE | -0.01 | 9466.8 | nan | TRUE | -0.01 | -0.11 | 0.01 | -0.01 | -0.02 |
| TCGA-DK-A1A7-01 | FALSE | TRUE | -0.01 | -2261.78 | 0 | FALSE | -0.12 | -0.01 | 0.02 | -0.05 | 0.03 |
| TCGA-CF-A47V-01 | FALSE | TRUE | -0.02 | -606.79 | 0 | FALSE | 0.43 | -0.02 | 0.02 | 0.01 | -0.03 |
| TCGA-CU-A0YN-01 | FALSE | TRUE | -0.02 | 813.17 | 1 | TRUE | -0.02 | 0.53 | 0.02 | 0.09 | -0.03 |
| TCGA-CF-A47S-01 | FALSE | TRUE | -0.02 | -461 | nan | FALSE | 0.15 | -0.02 | 0.02 | -0.03 | 0 |
| TCGA-4Z-AA7Y-01 | FALSE | TRUE | -0.02 | -1780.74 | nan | FALSE | -0.08 | -0.02 | 0.02 | 0 | -0.03 |
| TCGA-FD-A3N6-01 | FALSE | TRUE | -0.02 | 10716.12 | nan | TRUE | -0.02 | 0.04 | 0.01 | -0.01 | -0.01 |
| TCGA-FJ-A3ZE-01 | FALSE | TRUE | -0.02 | -2890.72 | nan | FALSE | -0.46 | -0.02 | 0.01 | -0.02 | 0.01 |
| TCGA-ZF-A9RF-01 | FALSE | TRUE | -0.02 | 18831.34 | 1 | TRUE | -0.02 | 0.39 | 0.02 | 0.05 | -0.02 |
| TCGA-ZF-AA51-01 | FALSE | TRUE | -0.03 | 2778.82 | 0 | TRUE | -0.03 | 0.08 | 0 | 0.05 | -0.03 |
| TCGA-ZF-A9R1-01 | FALSE | TRUE | -0.03 | -2077.67 | 0 | FALSE | 0.14 | -0.03 | 0.01 | -0.01 | 0 |
| TCGA-4Z-AA7S-01 | FALSE | TRUE | -0.03 | -3021.24 | nan | FALSE | -0.11 | -0.03 | 0.02 | -0.03 | 0.01 |
| TCGA-BT-A20J-01 | FALSE | TRUE | -0.03 | 10016.42 | 0 | TRUE | -0.03 | -0.06 | -0.01 | -0.01 | 0.01 |
| TCGA-E5-A4U1-01 | FALSE | TRUE | -0.04 | -3563.4 | 1 | FALSE | -0.07 | -0.04 | 0.05 | -0.1 | 0.04 |
| TCGA-DK-AA77-01 | FALSE | TRUE | -0.04 | 10812.86 | nan | TRUE | -0.04 | -0.09 | 0.05 | -0.04 | -0.02 |
| TCGA-K4-A3WV-01 | FALSE | TRUE | -0.04 | -2889.68 | 1 | FALSE | -0.12 | -0.04 | -0.01 | 0.02 | -0.01 |
| TCGA-GV-A3QF-01 | FALSE | TRUE | -0.04 | -3165.26 | 0 | FALSE | -0.15 | -0.04 | 0.01 | -0.02 | 0 |
| TCGA-PQ-A6FI-01 | FALSE | TRUE | -0.04 | 4251.8 | 0 | TRUE | -0.04 | 0.17 | 0.03 | 0 | -0.01 |
| TCGA-DK-AA76-01 | FALSE | TRUE | -0.05 | -3057.11 | 0 | FALSE | -0.18 | -0.05 | 0.03 | -0.05 | 0.02 |
| TCGA-CU-A5W6-01 | FALSE | TRUE | -0.05 | -1023.38 | 1 | FALSE | -0.08 | -0.05 | -0.01 | 0.02 | -0.02 |
| TCGA-CF-A8HX-01 | FALSE | TRUE | -0.05 | -3231.66 | 0 | FALSE | 0.12 | -0.05 | 0.04 | -0.07 | 0.01 |
| TCGA-E7-A5KE-01 | FALSE | TRUE | -0.05 | -3324.48 | 1 | FALSE | -0.26 | -0.05 | 0.01 | -0.03 | 0.01 |
| TCGA-BT-A20V-01 | FALSE | TRUE | -0.06 | -988.53 | 1 | FALSE | -0.11 | -0.06 | 0.03 | -0.04 | 0 |
| TCGA-BT-A20X-01 | FALSE | TRUE | -0.06 | 1924.88 | nan | TRUE | -0.06 | 0.36 | 0.02 | 0.03 | 0 |
| TCGA-E7-A4IJ-01 | FALSE | TRUE | -0.06 | 322.49 | 0 | TRUE | -0.06 | -0.4 | 0 | -0.05 | -0.01 |
| TCGA-GV-A6ZA-01 | FALSE | TRUE | -0.06 | 2798.46 | 0 | TRUE | -0.06 | -0.31 | 0.04 | -0.09 | 0 |
| TCGA-ZF-AA4N-01 | FALSE | TRUE | -0.06 | 970.08 | 0 | TRUE | -0.06 | 0.79 | 0.03 | 0.09 | 0 |
| TCGA-G2-A2EO-01 | FALSE | TRUE | -0.06 | 5077.01 | nan | TRUE | -0.06 | 1.38 | 0.06 | 0.13 | 0.02 |
| TCGA-DK-A1A3-01 | FALSE | TRUE | -0.06 | 4382.95 | 0 | TRUE | -0.06 | 0.5 | 0 | 0.06 | 0.02 |
| TCGA-BL-A0C8-01 | FALSE | TRUE | -0.09 | -1943.13 | 0 | FALSE | -0.16 | -0.09 | -0.03 | 0.01 | 0.01 |
| TCGA-XF-A8HB-01 | FALSE | TRUE | -0.09 | -548.4 | nan | FALSE | -0.24 | -0.09 | 0.05 | -0.08 | 0.02 |
| TCGA-GV-A3JW-01 | FALSE | TRUE | -0.09 | -3341.46 | 1 | FALSE | -0.17 | -0.09 | 0 | -0.04 | 0.02 |
| TCGA-G2-A2ES-01 | FALSE | TRUE | -0.09 | 15258.36 | nan | TRUE | -0.09 | 0.38 | 0.03 | 0.06 | -0.03 |
| TCGA-DK-A3IS-01 | FALSE | TRUE | -0.1 | 4123.64 | 0 | FALSE | -0.05 | -0.1 | 0.05 | -0.04 | -0.03 |
| TCGA-4Z-AA89-01 | FALSE | TRUE | -0.1 | -210.77 | nan | FALSE | 0.04 | -0.1 | 0.03 | -0.05 | -0.01 |
| TCGA-PQ-A6FN-01 | FALSE | TRUE | -0.11 | -1582.15 | nan | TRUE | -0.11 | 0.8 | 0.05 | 0.05 | 0.02 |
| TCGA-DK-A6AV-01 | FALSE | TRUE | -0.11 | -1084.94 | 0 | TRUE | -0.11 | -0.02 | 0.04 | -0.05 | 0.01 |
| TCGA-ZF-A9R5-01 | FALSE | TRUE | -0.12 | -729.09 | nan | FALSE | 0.03 | -0.12 | 0.01 | -0.05 | 0.01 |
| TCGA-XF-AAN5-01 | FALSE | TRUE | -0.12 | 12128.74 | nan | TRUE | -0.12 | 0.34 | 0.04 | 0.01 | -0.01 |
| TCGA-DK-A1A6-01 | FALSE | TRUE | -0.12 | 4678.83 | 0 | TRUE | -0.12 | -0.12 | 0.01 | -0.01 | -0.01 |
| TCGA-G2-AA3F-01 | FALSE | TRUE | -0.12 | -2418.67 | 0 | FALSE | -0.13 | -0.12 | 0 | -0.04 | 0.03 |
| TCGA-BL-A3JM-01 | FALSE | TRUE | -0.12 | -262.01 | 0 | TRUE | -0.12 | 0.13 | 0.02 | 0.01 | 0 |
| TCGA-GC-A3BM-01 | FALSE | TRUE | -0.12 | 1121.53 | 1 | FALSE | 0.05 | -0.12 | 0.02 | -0.04 | 0 |
| TCGA-CU-A3KJ-01 | FALSE | TRUE | -0.13 | 2735.37 | 0 | TRUE | -0.13 | -0.08 | 0 | 0.03 | -0.05 |
| TCGA-BT-A20W-01 | FALSE | TRUE | -0.13 | -1157.4 | 0 | TRUE | -0.13 | -0.03 | 0.02 | -0.05 | 0.02 |
| TCGA-CU-A0YR-01 | FALSE | TRUE | -0.14 | 6058.01 | 0 | TRUE | -0.14 | 0.17 | -0.01 | 0.05 | -0.01 |
| TCGA-ZF-A9R2-01 | FALSE | TRUE | -0.14 | -3000.92 | 1 | FALSE | -0.09 | -0.14 | 0.03 | -0.06 | 0.01 |
| TCGA-GC-A4ZW-01 | FALSE | TRUE | -0.15 | -1823.97 | 0 | TRUE | -0.15 | -0.6 | 0.01 | -0.09 | -0.02 |
| TCGA-MV-A51V-01 | FALSE | TRUE | -0.15 | -2734.43 | 0 | FALSE | 0.05 | -0.15 | 0 | -0.01 | -0.01 |
| TCGA-G2-AA3B-01 | FALSE | TRUE | -0.15 | 4792.81 | 0 | FALSE | -0.29 | -0.15 | 0.03 | -0.07 | 0.02 |
| TCGA-ZF-AA4V-01 | FALSE | TRUE | -0.17 | 5108.85 | 1 | TRUE | -0.17 | 0.59 | 0.04 | 0.08 | -0.03 |
| TCGA-CF-A27C-01 | FALSE | TRUE | -0.17 | -1983.54 | nan | FALSE | -0.03 | -0.17 | 0.02 | -0.07 | 0.02 |
| TCGA-CF-A3MG-01 | FALSE | TRUE | -0.17 | -3188.46 | nan | FALSE | -0.05 | -0.17 | 0.01 | -0.04 | 0 |
| TCGA-DK-AA6W-01 | FALSE | TRUE | -0.17 | 203.85 | 0 | FALSE | -0.37 | -0.17 | 0.02 | -0.07 | 0.03 |
| TCGA-G2-A2EK-01 | FALSE | TRUE | -0.19 | -1148.59 | 0 | FALSE | 0.02 | -0.19 | -0.03 | -0.03 | 0.03 |
| TCGA-E7-A85H-01 | FALSE | TRUE | -0.2 | 1266.73 | 0 | TRUE | -0.2 | -0.01 | 0.02 | -0.04 | 0.02 |
| TCGA-DK-A6B1-01 | FALSE | TRUE | -0.22 | 4144.05 | 1 | FALSE | -0.14 | -0.22 | -0.02 | -0.01 | -0.01 |
| TCGA-GU-A42P-01 | FALSE | TRUE | -0.22 | -3212.13 | 0 | FALSE | -0.24 | -0.22 | 0 | -0.06 | 0.02 |
| TCGA-ZF-A9R3-01 | FALSE | TRUE | -0.23 | 1120.14 | 0 | FALSE | 0.25 | -0.23 | -0.02 | 0.02 | -0.03 |
| TCGA-G2-A2EL-01 | FALSE | TRUE | -0.23 | -860.76 | nan | FALSE | -0.36 | -0.23 | 0.04 | -0.09 | 0.01 |
| TCGA-CF-A47X-01 | FALSE | TRUE | -0.24 | -2615.99 | nan | FALSE | -0.13 | -0.24 | 0.03 | -0.08 | 0.02 |
| TCGA-ZF-A9RN-01 | FALSE | TRUE | -0.24 | 18937.47 | 0 | TRUE | -0.24 | -0.16 | 0.02 | -0.06 | 0.01 |
| TCGA-4Z-AA80-01 | FALSE | TRUE | -0.26 | -252.52 | 0 | FALSE | -0.45 | -0.26 | 0.02 | -0.07 | 0.01 |
| TCGA-ZF-A9R4-01 | FALSE | TRUE | -0.27 | 6408.89 | 1 | TRUE | -0.27 | -0.19 | 0.02 | -0.06 | 0.01 |
| TCGA-GU-A42R-01 | FALSE | TRUE | -0.27 | -2892.33 | 0 | FALSE | 0.05 | -0.27 | 0 | -0.04 | 0 |
| TCGA-CF-A9FM-01 | FALSE | TRUE | -0.29 | -1700.43 | nan | FALSE | 0.25 | -0.29 | -0.01 | -0.02 | -0.01 |
| TCGA-C4-A0F0-01 | FALSE | TRUE | -0.29 | 2165.81 | nan | TRUE | -0.29 | 0.81 | 0.06 | 0.04 | 0.02 |
| TCGA-CU-A3YL-01 | FALSE | TRUE | -0.29 | -2015.88 | 0 | FALSE | 0.47 | -0.29 | 0 | -0.06 | 0.01 |
| TCGA-XF-A8HC-01 | FALSE | TRUE | -0.29 | -3242.03 | nan | FALSE | -0.19 | -0.29 | 0 | -0.06 | 0.01 |
| TCGA-XF-A9T0-01 | FALSE | TRUE | -0.29 | 789.58 | nan | TRUE | -0.29 | 0.81 | 0.04 | 0.04 | 0.04 |
| TCGA-DK-AA6P-01 | FALSE | TRUE | -0.3 | -2985.37 | 0 | FALSE | -0.21 | -0.3 | -0.01 | -0.05 | 0.01 |
| TCGA-XF-AAN0-01 | FALSE | TRUE | -0.3 | 1507.65 | 1 | FALSE | 0.17 | -0.3 | -0.02 | -0.02 | -0.01 |
| TCGA-E7-A7PW-01 | FALSE | TRUE | -0.3 | -2351.27 | 0 | FALSE | -0.11 | -0.3 | 0.01 | -0.07 | 0.02 |
| TCGA-HQ-A2OF-01 | FALSE | TRUE | -0.31 | -2633.63 | 0 | FALSE | -0.43 | -0.31 | 0.02 | -0.09 | 0.02 |
| TCGA-KQ-A41Q-01 | FALSE | TRUE | -0.32 | -3084.78 | 0 | FALSE | -0.35 | -0.32 | 0.01 | -0.06 | -0.01 |
| TCGA-ZF-AA4T-01 | FALSE | TRUE | -0.32 | 36.15 | 0 | FALSE | -0.21 | -0.32 | 0.01 | -0.06 | 0 |
| TCGA-K4-A6FZ-01 | FALSE | TRUE | -0.32 | 5651.59 | 1 | FALSE | 0.23 | -0.32 | 0.02 | -0.01 | -0.06 |
| TCGA-FJ-A3ZF-01 | FALSE | TRUE | -0.33 | -2712.84 | 0 | FALSE | -0.36 | -0.33 | 0.01 | -0.1 | 0.04 |
| TCGA-GV-A3QI-01 | FALSE | TRUE | -0.35 | -2839.5 | nan | FALSE | -0.29 | -0.35 | -0.01 | -0.06 | 0.02 |
| TCGA-ZF-A9RE-01 | FALSE | TRUE | -0.35 | -134.43 | nan | FALSE | -0.11 | -0.35 | 0.01 | -0.04 | -0.03 |
| TCGA-4Z-AA87-01 | FALSE | TRUE | -0.36 | -22.62 | 0 | TRUE | -0.36 | 0.34 | 0.05 | -0.04 | 0.03 |
| TCGA-GV-A40G-01 | FALSE | TRUE | -0.38 | -1766.22 | 0 | FALSE | -0.19 | -0.38 | 0.01 | -0.06 | 0 |
| TCGA-ZF-A9RL-01 | FALSE | TRUE | -0.38 | -2850.93 | 0 | FALSE | -0.23 | -0.38 | -0.01 | -0.09 | 0.04 |
| TCGA-DK-A6B5-01 | FALSE | TRUE | -0.38 | 4439.03 | 0 | FALSE | 0.16 | -0.38 | -0.03 | 0 | -0.03 |
| TCGA-CF-A1HR-01 | FALSE | TRUE | -0.39 | 551.48 | 0 | FALSE | 0.38 | -0.39 | -0.04 | -0.01 | -0.02 |
| TCGA-DK-AA6U-01 | FALSE | TRUE | -0.4 | -1968.31 | 0 | FALSE | -0.17 | -0.4 | 0.01 | -0.09 | 0.03 |
| TCGA-XF-AAML-01 | FALSE | TRUE | -0.44 | 24349.15 | 0 | FALSE | -0.14 | -0.44 | 0.01 | -0.07 | -0.01 |
| TCGA-E7-A6MF-01 | FALSE | TRUE | -0.45 | -1755.27 | nan | FALSE | 0.17 | -0.45 | -0.02 | -0.07 | 0.02 |
| TCGA-XF-A8HI-01 | FALSE | TRUE | -0.45 | -1904.44 | nan | FALSE | 0.09 | -0.45 | 0.01 | -0.07 | -0.01 |
| TCGA-CU-A3QU-01 | FALSE | TRUE | -0.46 | 5444.87 | 1 | FALSE | -0.24 | -0.46 | -0.01 | -0.05 | -0.01 |
| TCGA-GV-A3QH-01 | FALSE | TRUE | -0.46 | -328.9 | 0 | FALSE | 0.02 | -0.46 | 0 | -0.05 | -0.02 |
| TCGA-H4-A2HQ-01 | FALSE | TRUE | -0.47 | 231.34 | 0 | FALSE | -0.21 | -0.47 | 0.01 | -0.07 | -0.01 |
| TCGA-DK-A1AC-01 | FALSE | TRUE | -0.48 | 2685.24 | 1 | TRUE | -0.48 | 0.11 | 0.01 | 0.02 | -0.02 |
| TCGA-DK-AA6X-01 | FALSE | TRUE | -0.49 | 6990.92 | 0 | FALSE | 0.12 | -0.49 | -0.05 | -0.01 | -0.01 |
| TCGA-E7-A5KF-01 | FALSE | TRUE | -0.49 | -1044.01 | nan | FALSE | 0 | -0.49 | 0.01 | -0.08 | 0 |
| TCGA-BT-A42C-01 | FALSE | TRUE | -0.52 | -2263.79 | 0 | FALSE | -0.24 | -0.52 | -0.03 | -0.05 | 0 |
| TCGA-DK-A6B6-01 | FALSE | TRUE | -0.53 | 10018.14 | nan | FALSE | 0.11 | -0.53 | -0.01 | -0.07 | 0 |
| TCGA-HQ-A2OE-01 | FALSE | TRUE | -0.55 | -1939.68 | 0 | FALSE | -0.45 | -0.55 | 0.01 | -0.09 | 0 |
| TCGA-FD-A6TE-01 | FALSE | TRUE | -0.55 | 8365.93 | nan | FALSE | -0.27 | -0.55 | 0.01 | -0.06 | -0.04 |
| TCGA-E5-A4TZ-01 | FALSE | TRUE | -0.58 | -2113.44 | 0 | FALSE | -0.01 | -0.58 | -0.01 | -0.08 | 0 |
| TCGA-2F-A9KR-01 | FALSE | TRUE | -0.58 | 64.37 | nan | FALSE | 0.04 | -0.58 | 0 | -0.05 | -0.03 |
| TCGA-E7-A6ME-01 | FALSE | TRUE | -0.6 | 8624.14 | 0 | FALSE | 0.06 | -0.6 | -0.01 | -0.09 | 0.01 |
| TCGA-G2-A3VY-01 | FALSE | TRUE | -0.64 | -963.76 | 0 | FALSE | -0.24 | -0.64 | -0.03 | -0.09 | 0.02 |
| TCGA-G2-AA3D-01 | FALSE | TRUE | -0.66 | -2771.65 | 1 | FALSE | 0.26 | -0.66 | 0 | -0.09 | -0.01 |
| TCGA-ZF-A9RM-01 | FALSE | TRUE | -0.78 | -3347.36 | 0 | FALSE | -0.22 | -0.78 | -0.02 | -0.1 | 0 |
| TCGA-E7-A8O7-01 | FALSE | TRUE | -0.79 | 1512.01 | 1 | FALSE | 0.33 | -0.79 | -0.03 | -0.06 | -0.03 |
| TCGA-LT-A5Z6-01 | FALSE | TRUE | -0.88 | -1813.94 | nan | FALSE | 0.03 | -0.88 | -0.03 | -0.12 | 0.01 |
| TCGA-GV-A3JX-01 | FALSE | TRUE | -1.08 | 7142.28 | 0 | FALSE | 0.15 | -1.08 | -0.03 | -0.07 | -0.06 |
| TCGA-ZF-AA4X-01 | FALSE | TRUE | -1.19 | 1577.98 | 1 | FALSE | -0.08 | -1.19 | -0.02 | -0.16 | 0 |
| TCGA-DK-A3IV-01 | FALSE | TRUE | -1.4 | 5122.29 | 0 | FALSE | 0.33 | -1.4 | -0.05 | -0.11 | -0.05 |
