# Supplementary material for: Identification of autophagy‐related genes signature predicts chemotherapeutic and immunotherapeutic efficiency in bladder cancer (BLCA)
Source: J Cell Mol Med. 2021 May 7;25(12):5417–33. doi: 10.1111/jcmm.16552 (PMC8184684; doi:10.1111/jcmm.16552)
Supplement: Supplementary file 22 — Supplementary Material [file JCMM-25-5417-s007.docx]

**Additional File Materials**

Supplementary Table 1. Brief information of GEO cohorts in the study.

Supplementary Table 2. Summary of detailed clinical information of TCGA-BLCA cohort.

Supplementary Table 3. The abundance of estimated TME immune cell infiltration in TCGA-BLCA cohort.

Supplementary Table 4. The detailed information of immunotherapy response based on TIDE algorithm in TCGA-BLCA cohort.

Supplementary Figure 1. A flow diagram and design of the study.

Supplementary Figure 2. Difference in the enrichment of autophagy related signalling pathways between ATGRS high-risk and low-risk groups in TCGA-BLCA cohort.

Supplementary Figure 3. Comparison of the prognostic prediction efficiency between ATGRS and wang’s model. (A) Kaplan-Meier survival curves showed the difference in OS advantage between ATGRS high-risk and low-risk groups in TCGA-BLCA cohort (Log-rank test, p = 0.0025), GSE13507 cohort (Log-rank test, p = 0.0076), and GSE48075 cohort (Log-rank test, p = 0.32) based on wang’s model. (B) Kaplan-Meier survival curves showed the difference in DFS advantage between ATGRS high-risk and low-risk groups in TCGA-BLCA cohort (Log-rank test, p = 0.015), GSE13507 cohort (Log-rank test, p = 0.027), GSE48075 cohort (Log-rank test, p = 0.25) and GSE32894 (Log-rank test, p = 0.1) based on wang’s model. (C) Bar plot showed the comparison of AUC of OS and DFS prediction at 1 year, 3 year, and 5 year between ATGRS and wang’s model through time-dependent ROC analysis.

Supplementary Figure 4. Differences in ATGRS risk-score between different molecular subtypes in TCGA-BLCA cohort. The upper and lower ends of the boxes represented interquartile range of values. The lines in the boxes represented median value. Student t tests and one-way Anova tests were used to compare the statistical difference between UNC (A), MDA (B), TCGA (C), CC (D), Lund1 (E), Lund2 (F) and TCGAcluster (G) molecular classification systems..

Supplementary Figure 5. Kaplan-Meier survival stratification analyses in TCGA-BLCA cohort. (A) Female; (B) Male; (C) Age≤65 years; (D) Age >65 years; (E) Papillary; (F) Non‐papillary; (G) Pathology T0‐T2; (H) Pathology T3‐T4; (I) Pathology N0; (J) Pathology N+; (K) Pathology M0; (L) Pathology M1+Mx; (M) Stage I/II; (N) Stage III/IV; (O) Number of positive lymph nodes by HE-; (P) Number of positive lymph nodes by HE+; (Q) Lymphovascular invasion-; (R) Lymphovascular invasion+.

Supplementary Figure 6. ATGRS is associated with TME immune cells infiltration. (A) Difference in the infiltration of TME immune cells between high and low risk ATGRS groups. The upper and lower ends of the boxes represented interquartile range of values. The lines in the boxes represented median value and the black dots showed outliers. The statistical difference was tested by the student t test and the immune cells with no statistical difference were shown in figure S6.

Supplementary Figure 7. Pancancer validation of ATGRS. Forest plot summary of the univariate cox analysis of ATGRS in 32 different cancer types. The blue diamond squares on the transverse lines represent the HR, and the black transverse lines represent the 95% CI. The p value and 95% CI for each clinical feature are displayed in detail.

Supplementary Figure 8. Prediction utility of ATGRS in 32 cancer types from TCGA database. Kaplan-Meier survival curves showed the difference in OS advantage between ATGRS high-risk and low-risk groups. (A) TCGA Adrenocortical Cancer (ACC); (B) TCGA Acute Myeloid Leukemia (LAML); (C) TCGA Breast Cancer (BRCA); (D) TCGA Cervical Cancer (CESC); (E) TCGA Bile Duct Cancer (CHOL); (F) TCGA Colon Cancer (COAD); (G) TCGA Large B-cell Lymphoma (DLBC); (H) TCGA Esophageal Cancer (ESCA); (I) TCGA Glioblastoma (GBM); (J) TCGA Head and Neck Cancer (HNSC); (K) TCGA Kidney Chromophobe (KICH); (L) TCGA Kidney Clear Cell Carcinoma (KIRC); (M) TCGA Kidney Papillary Cell Carcinoma (KIRP); (N) TCGA Lower Grade Glioma (LGG); (O) TCGA Liver Cancer (LIHC); (P) TCGA Lung Adenocarcinoma (LUAD); (Q) TCGA Lung Squamous Cell Carcinoma (LUSC); (R) TCGA Mesothelioma (MESO); (S) TCGA Ovarian Cancer (OV); (T) TCGA Pancreatic Cancer (PAAD); (U) TCGA Pheochromocytoma & Paraganglioma (PCPG); (V) TCGA Prostate Cancer (PRAD); (W) TCGA Rectal Cancer (READ); (X) TCGA Sarcoma (SARC); (Y) TCGA Melanoma (SKCM); (Z) TCGA Stomach Cancer (STAD); (AA) TCGA Testicular Cancer (TGCT); (AB) TCGA Thyroid Cancer (THCA); (AC) TCGA Thymoma (THYM); (AD) TCGA Endometrioid Cancer (UCEC); (AE) TCGA Uterine Carcinosarcoma (UCS); (AF) TCGA Ocular melanomas (UVM).

Supplementary Figure 9. Abnormal protein expression of APOL1 between normal bladder and bladder cancer tissues based on human protein atlas. (A) IHC staining level in bladder cancer tissues; (B) IHC intensity level in bladder cancer tissues; (C) IHC quantity level in bladder cancer tissues; (D) IHC staining level in normal bladder tissues; (E) IHC intensity level in normal bladder tissues; (F) IHC quantity level in normal bladder tissues; (G) Number of bladder cancer/normal bladder samples in different IHC staining level; (H) Number of bladder cancer/normal bladder samples in different IHC intensity level; (I) Number of bladder cancer/normal bladder samples in different IHC quantity level; (J) Representative images of protein expression between normal bladder and bladder cancer tissues.

Supplementary Figure 10. Abnormal protein expression of ATF6 between normal bladder and bladder cancer tissues based on human protein atlas. (A) IHC staining level in bladder cancer tissues; (B) IHC intensity level in bladder cancer tissues; (C) IHC quantity level in bladder cancer tissues; (D) IHC staining level in normal bladder tissues; (E) IHC intensity level in normal bladder tissues; (F) IHC quantity level in normal bladder tissues; (G) Number of bladder cancer/normal bladder samples in different IHC staining level; (H) Number of bladder cancer/normal bladder samples in different IHC intensity level; (I) Number of bladder cancer/normal bladder samples in different IHC quantity level; (J) Representative images of protein expression between normal bladder and bladder cancer tissues.

Supplementary Figure 11. Abnormal protein expression of ATP6V0A1 between normal bladder and bladder cancer tissues based on human protein atlas. (A) IHC staining level in bladder cancer tissues; (B) IHC intensity level in bladder cancer tissues; (C) IHC quantity level in bladder cancer tissues; (D) IHC staining level in normal bladder tissues; (E) IHC intensity level in normal bladder tissues; (F) IHC quantity level in normal bladder tissues; (G) Number of bladder cancer/normal bladder samples in different IHC staining level; (H) Number of bladder cancer/normal bladder samples in different IHC intensity level; (I) Number of bladder cancer/normal bladder samples in different IHC quantity level; (J) Representative images of protein expression between normal bladder and bladder cancer tissues.

Supplementary Figure 12. Abnormal protein expression of EGFR between normal bladder and bladder cancer tissues based on human protein atlas. (A) IHC staining level in bladder cancer tissues; (B) IHC intensity level in bladder cancer tissues; (C) IHC quantity level in bladder cancer tissues; (D) IHC staining level in normal bladder tissues; (E) IHC intensity level in normal bladder tissues; (F) IHC quantity level in normal bladder tissues; (G) Number of bladder cancer/normal bladder samples in different IHC staining level; (H) Number of bladder cancer/normal bladder samples in different IHC intensity level; (I) Number of bladder cancer/normal bladder samples in different IHC quantity level; (J) Representative images of protein expression between normal bladder and bladder cancer tissues.

Supplementary Figure 13. Abnormal protein expression of MYC between normal bladder and bladder cancer tissues based on human protein atlas. (A) IHC staining level in bladder cancer tissues; (B) IHC intensity level in bladder cancer tissues; (C) IHC quantity level in bladder cancer tissues; (D) IHC staining level in normal bladder tissues; (E) IHC intensity level in normal bladder tissues; (F) IHC quantity level in normal bladder tissues; (G) Number of bladder cancer/normal bladder samples in different IHC staining level; (H) Number of bladder cancer/normal bladder samples in different IHC intensity level; (I) Number of bladder cancer/normal bladder samples in different IHC quantity level; (J) Representative images of protein expression between normal bladder and bladder cancer tissues.

Supplementary Figure 14. Abnormal protein expression of P4HB between normal bladder and bladder cancer tissues based on human protein atlas. (A) IHC staining level in bladder cancer tissues; (B) IHC intensity level in bladder cancer tissues; (C) IHC quantity level in bladder cancer tissues; (D) IHC staining level in normal bladder tissues; (E) IHC intensity level in normal bladder tissues; (F) IHC quantity level in normal bladder tissues; (G) Number of bladder cancer/normal bladder samples in different IHC staining level; (H) Number of bladder cancer/normal bladder samples in different IHC intensity level; (I) Number of bladder cancer/normal bladder samples in different IHC quantity level; (J) Representative images of protein expression between normal bladder and bladder cancer tissues.

Supplementary Figure 15. Abnormal protein expression of SPNS1 between normal bladder and bladder cancer tissues based on human protein atlas. (A) IHC staining level in bladder cancer tissues; (B) IHC intensity level in bladder cancer tissues; (C) IHC quantity level in bladder cancer tissues; (D) IHC staining level in normal bladder tissues; (E) IHC intensity level in normal bladder tissues; (F) IHC quantity level in normal bladder tissues; (G) Number of bladder cancer/normal bladder samples in different IHC staining level; (H) Number of bladder cancer/normal bladder samples in different IHC intensity level; (I) Number of bladder cancer/normal bladder samples in different IHC quantity level; (J) Representative images of protein expression between normal bladder and bladder cancer tissues.

Supplementary Figure 16. Abnormal protein expression of TP53INP1 between normal bladder and bladder cancer tissues based on human protein atlas. (A) IHC staining level in bladder cancer tissues; (B) IHC intensity level in bladder cancer tissues; (C) IHC quantity level in bladder cancer tissues; (D) IHC staining level in normal bladder tissues; (E) IHC intensity level in normal bladder tissues; (F) IHC quantity level in normal bladder tissues; (G) Number of bladder cancer/normal bladder samples in different IHC staining level; (H) Number of bladder cancer/normal bladder samples in different IHC intensity level; (I) Number of bladder cancer/normal bladder samples in different IHC quantity level; (J) Representative images of protein expression between normal bladder and bladder cancer tissues.

Supplementary Figure 17. Abnormal protein expression of ZC3H12A between normal bladder and bladder cancer tissues based on human protein atlas. (A) IHC staining level in bladder cancer tissues; (B) IHC intensity level in bladder cancer tissues; (C) IHC quantity level in bladder cancer tissues; (D) IHC staining level in normal bladder tissues; (E) IHC intensity level in normal bladder tissues; (F) IHC quantity level in normal bladder tissues; (G) Number of bladder cancer/normal bladder samples in different IHC staining level; (H) Number of bladder cancer/normal bladder samples in different IHC intensity level; (I) Number of bladder cancer/normal bladder samples in different IHC quantity level; (J) Representative images of protein expression between normal bladder and bladder cancer tissues.
